# Design, Synthesis and Bioactivity Evaluation of Heterocycle-Containing Mono- and Bisphosphonic Acid Compounds

**DOI:** 10.3390/molecules28227509

**Published:** 2023-11-09

**Authors:** Xin Wu, Zili Yang, Mengwei Bu, Jiang Duan, Aidong Zhang

**Affiliations:** National Key Laboratory of Green Pesticide, College of Chemistry, Central China Normal University, Wuhan 430079, China; wuxin960106@163.com (X.W.); m17720488373@163.com (Z.Y.); mengweibu01@163.com (M.B.)

**Keywords:** monophosphonic acid, bisphosphonic acid, heterocycle, DXR, herbicidal activity

## Abstract

Fosmidomycin (FOS) is a naturally occurring compound active against the 1-deoxy-D-xylulose 5-phosphate reductoisomerase (DXR) enzyme in the 2-*C*-methyl-D-erythritol 4-phosphate (MEP) pathway, and using it as a template for lead structure design is an effective strategy to develop new active compounds. In this work, by replacing the hydroxamate unit of FOS with pyrazole, isoxazole and the related heterocycles that also have metal ion binding affinity, while retaining the monophosphonic acid in FOS or replacing it with a bisphosphonic acid group, heterocycle-containing mono- and bisphosphonic acid compounds as FOS analogs were designed. The key steps involved in the facile synthesis of these FOS analogs included the Michael addition of diethyl vinylphosphonate or tetraethyl vinylidenebisphosphonate to β-dicarbonyl compounds and the subsequent cyclic condensation with hydrazine or hydroxylamine. Two additional isoxazolinone-bearing FOS analogs were synthesized via the Michaelis–Becker reaction with diethyl phosphite as a key step. The bioactivity evaluation on model plants demonstrated that several compounds have better herbicidal activities compared to FOS, with the most active compound showing a 3.7-fold inhibitory activity on *Arabidopsis thaliana*, while on the roots and stalks of *Brassica napus* L. and *Echinochloa crus-galli* in a pre-emergence inhibitory activity test, the activities of this compound were found to be 3.2- and 14.3-fold and 5.4- and 9.4-fold, respectively, and in a post-emergency activity test on *Amaranthus retroflexus* and *Echinochloa crus-galli*, 2.2- and 2.0-fold inhibition activities were displayed. Despite the significant herbicidal activity, this compound exhibited a DXR inhibitory activity lower than that of FOS but comparable to that of other non-hydroxamate DXR inhibitors, and the dimethylallyl pyrophosphate rescue assay gave no statistical significance, suggesting that a different target might be involved in the inhibiting process. This work demonstrates that using bioisosteric replacement can be considered as a valuable strategy to discover new FOS analogs that may have high herbicidal activities.

## 1. Introduction

**Figure 1:**
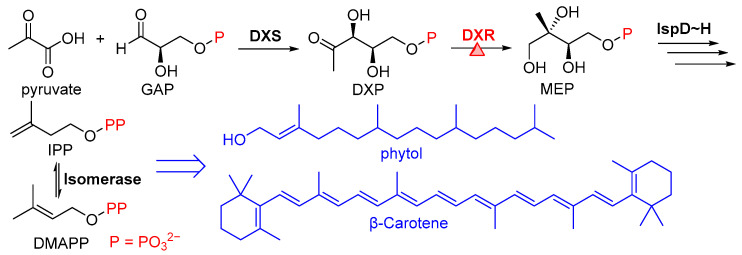
The MEP pathway for the synthesis of the isoprenoid precursors, IPP and DMAPP, that lead to two essential isoprenoid products, phytol and β-carotene, as examples in plants, with the function of DXR enzyme in converting DXP to MEP highlighted.

The 2-*C*-methyl-D-erythritol 4-phosphate (MEP) pathway, which is widespread in bacteria and plants but absent in mammals [1], has gained significant attention in recent years since seven key enzymes are involved in this pathway (Figure 1), which can serve as new targets to develop novel herbicides and antibacterial agents [2,3]. The products of the MEP pathway, isopentenyl diphosphate (IPP) and dimethylallyl diphosphate (DMAPP), are crucial precursors for the synthesis of various isoprenoids [4]. For example, plants utilize IPP and DMAPP produced via the MEP pathway to synthesize phytol, β-carotene and other substances that are essential to the photosynthesis process and physiological regulation (Figure 1) [5]. The second enzyme in this pathway, 1-deoxy-D-xylulose 5-phosphate reductoisomerase, shortly named DXR and also known as IspC, which catalyzes the isomerization and reduction of 1-deoxy-D-xylulose 5-phosphate (DXP) to produce MEP [6], is the most encouraging target for the development of novel antibacterial and antimalarial drugs because two naturally occurring products, fosmidomycin (FOS) and FR900098 (FR), have been found active in targeting this enzyme, and the inhibition mechanism has been well documented [7,8]. Although DXR has been considered a promising target for herbicides [9,10], the research focusing on the herbicide discovery based on DXR is rather limited, making the development of DXR inhibitors as herbicidal lead compounds a work of great significance.

**Figure 2:**
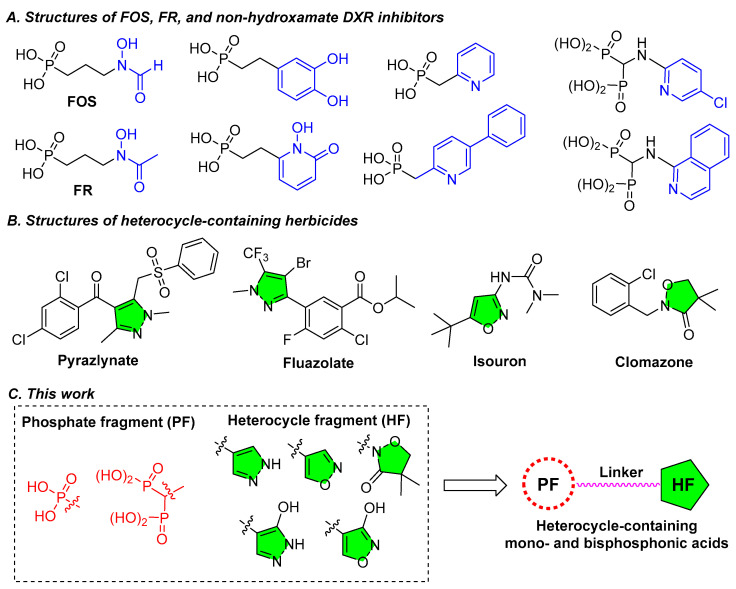
Structures of two naturally occurring DXR inhibitors, FOS and FR, and reported non-hydroxamate DXR inhibitors (**A**), exemplified nitrogen-containing heterocycles in commercial herbicides (**B**), and the design strategy for heterocycle-containing mono- and bisphosphonic acids in this work (**C**).

The two natural products, FOS and FR, originally isolated from *Streptomyces* species [11,12], are structurally composed of three parts, namely phosphonic acid, hydroxamate and the trimethylene linker, as shown in Figure 2A. FOS and FR are the substrate competitive inhibitors of DXR and have been well characterized, showing a wide range of bioactivities including antibacterial and antimalarial effects [13]. The FOS and FR themselves, however, have some drawbacks that need to be overcome, such as low lipophilicity, poor pharmacokinetics and low bioavailability, which impede their applications as clinic drugs [14]. Nevertheless, they have been extensively employed as templates for developing new related drugs, and a variety of structural modifications on FOS have been carried out [15]. Among the most potent DXR inhibitors thus developed are those structurally similar to FOS, all containing the hydroxamate as a binding group to the metal ion in the DXR active site [13,14,15]. Unfortunately, the hydroxamate group not only exhibits metabolic instability but also has strong side effects on various metal enzymes [16,17,18]; even the approved hydroxamate-containing drugs, such as vorinostat and panobinostat, tend to have low bioavailability [18]. Using non-hydroxamate groups to replace the hydroxamate group has become an effective method to improve the bioavailability of the related drugs [19,20].

Under this context, several works on the use of non-hydroxamate groups to replace the hydroxamate group of FOS and FR to develop DXR inhibitors have been reported [21,22,23,24], and their structures are given in Figure 2A. Structurally, these non-hydroxamate DXR inhibitors also consist of three fragments: mono- or bisphosphonic acid, linker and a non-hydroxamate group, but the interaction patterns of these compounds with the active site of DXR are different from FOS. For example, the catechol- and *N*-hydroxypyridone-containing FOS analogs were supposed to bind to the metal ion of the DXR active site but with moderate *Mt*DXR inhibitory activities (IC_50_ = 41 and 53 μM, respectively, versus 0.08 μM for FOS) [21]. The crystal X-ray analyses indicated that the pyridine and quinoline-containing FOS analogs are unable to bind with the metal ion, but can induce conformational changes in the active site of DXR to create a lipophilic pocket accommodating the pyridine or quinoline group that leads to inhibitory activity against *Ec*DXR, with an IC_50_ value as low as 0.84 μM [22]. Furthermore, some examples demonstrated that the bisphosphonate group can bind with the metal ion, while the pyridine or isoquinoline group occupies the hydrophobic cavity in the active site of DXR, resulting in IC_50_ values of 4 and 7 μM against *Ec*DXR [23]. Although these non-hydroxamate inhibitors are inferior to FOS in DXR inhibitory activity, some of them still have similar or higher anti-infective activities compared to FOS, possibly due to the improved bioavailability or the inhibition of other targets [21,22,23,24].

On the other hand, heterocycle-containing organophosphorus compounds are widespread in drugs, active molecules, functional materials and pesticides, and heterocycles, as important structural moieties, have been extensively used in the design of agrochemicals due to their structural diversity and bioisosteric replacement ability [25]. For example, commercial herbicides such as pyrazlynate and fluazolate contain a pyrazole ring, isouron features the presence of an isoxazole ring, and especially the herbicide, clomazone, which contains an isoxazolinone and is activated by metabolism in weeds, targets the DXS enzyme, the first enzyme in the MEP pathway [26] (Figure 2B). In addition, nitrogen-containing heterocycles such as pyrazole, pyrazolone and isoxazole can form monodentate coordination with divalent metal ions and have been widely employed as active substructural moieties in the design of metalloenzyme inhibitors [27]. Considering the advantages of heterocycles in herbicide compound design and their ability to bind metal ions, the replacement of the hydroxamate of FOS with a suitable heterocycle would provide an opportunity to develop DXR inhibitors with improved herbicidal activities while reducing the issue regarding a low bioavailability of the hydroxamate group.

Given the drawbacks of the hydroxamate group in DXR inhibitors, the advances of non-hydroxamate DXR inhibitors and the bioisosteric role of nitrogen-containing heterocycles, in this work, heterocycle-containing mono- and bisphosphonic acid compounds were designed, and their substructural complements and general structures are shown in Figure 2C. The phosphonic acid in FOS is either retained or substituted with a bisphosphonic acid group, and the hydroxamate is replaced with a heterocycle such as pyrazole, isoxazole or isoxazolinone. To synthesize these compounds, several key steps, such as the Michael addition of diethyl vinylphosphonate or tetraethyl vinylidenebisphosphonate to β-dicarbonyl compounds, the cyclic condensation with hydrazine or hydroxylamine and the Michaelis–Becker reaction with diethyl phosphite, were employed. These compounds were then tested for their activities in inhibiting model plants and the DXR enzyme. Other techniques such as a DMAPP rescue assay and molecular docking were also used to explore the possible action mechanism of active compounds.

## 2. Results and Discussion

### 2.1. Chemistry

**Scheme 1:**
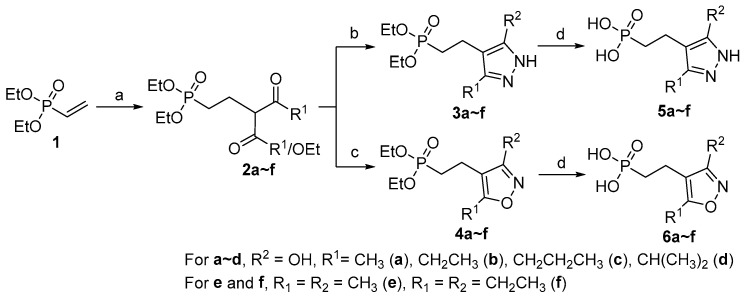
Synthesis of the monophosphonic acid series **5** and **6**. Reagents and conditions: (a) RCOCH_2_COOEt or RCOCH_2_COR, K_2_CO_3_, TEBAC, CH_3_CN, 80 °C; (b) N_2_H_4_·H_2_O, EtOH, 75 °C; (c) NH_2_OH·HCl, K_2_CO_3_, EtOH, 75 °C; (d) (i) TMSBr, CH_2_Cl_2_, 0 °C to r.t.; (ii) THF/H_2_O, r.t.

Synthesis routes. The cyclic condensation reaction of β-dicarbonyl compounds with hydrazine and hydroxylamine is one efficient method of synthesizing pyrazole and isoxazole derivatives [28]. Based on this reaction as a key step, in this work, a three-step synthesis route to the monophosphonic acid compounds **5** and **6** was designed, as shown in Scheme 1. First, diethyl vinylphosphonate (**1**), as a Michael acceptor, reacted with β-ketoesters or β-diketones in the presence of K_2_CO_3_ and benzyltriethylammonium chloride (TEBAC) to yield the intermediate **2**. In the second step, **2** was used to react with hydrazine or hydroxylamine, respectively, giving the corresponding cyclization intermediates **3** and **4**. Finally, the ethyl protecting groups were removed with TMSBr to yield the target products **5** and **6** in 70~82% overall yields.

**Scheme 2:**
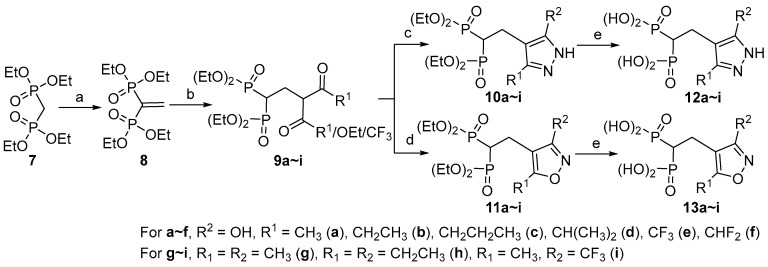
Synthesis of the bisphosphonic acid series **12** and **13**. Reagents and conditions: (a) (i) (CHO)_n_, Et_2_NH, MeOH, 65 °C; (ii) TsOH, toluene, 110 °C; (b) RCOCH_2_COOEt or RCOCH_2_COR, LiHMDS, THF, 0 °C to r.t.; (c) N_2_H_4_·H_2_O, EtOH, 75 °C; (d) NH_2_OH·HCl, K_2_CO_3_, EtOH, 75 °C; (e) (i) TMSBr, CH_2_Cl_2_, 0 °C to r.t.; (ii) THF/H_2_O, r.t.

A similar synthesis route was used to access the bisphosphonic acid series **12** and **13**, as shown in Scheme 2. First, a Michael acceptor tetraethyl vinylidenebisphosphonate (VBP) was synthesized through the reaction of tetraethyl methylenebisphosphonate (**7**) with paraformaldehyde in methanol, followed by dehydration with the catalysis of TsOH [29]. Then, tetraethyl VBP (**8**) reacted with β-ketoesters or β-diketones to give the intermediate **9** using lithium bis(trimethylsilyl)amide (LiHMDS) as a base. Cyclic condensation with hydrazine or hydroxylamine followed by deprotection gave the target compounds **12** and **13** in 75~88% overall yields.

**Scheme 3:**
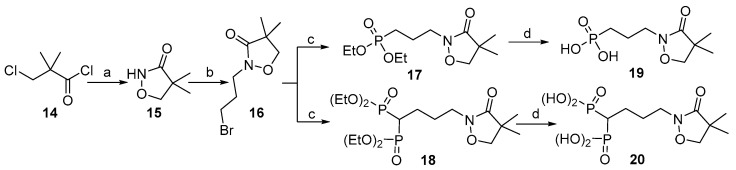
Synthesis of the target compounds **19** and **20**. (a) (i) NH_2_OH·HCl, NaOH, H_2_O, 0 °C to r.t.; (ii) NaOH, Na_2_CO_3_, H_2_O, 45 °C; (b) Br(CH_2_)_3_Br, NaH, DMF, 0 °C to r.t.; (c) HPO(OEt)_2_ or **7**, Cs_2_CO_3_, TBAI, DMF, r.t.; (d) (i) TMSBr, CH_2_Cl_2_, 0 °C to r.t.; (ii) THF/H_2_O, r.t.

The synthesis route for isoxazolinone-containing compounds **19** and **20** is different from the previous ones, and the Michaelis–Becker reaction was used as a key step, as shown in Scheme 3. In this route, with 3-chloropivaloyl chloride (**14**) as the starting material, and through the amidation with hydroxylamine, followed by cyclization [30], the intermediate 4,4-dimethylisoxazolidin-3-one (**15**) was obtained. Nucleophilic substitution on **15** with 1,3-dibromopropane in the presence of NaH yielded the brominated intermediate **16**. Next, the Michaelis–Becker reaction of **16** with diethyl phosphite (**1**) and methylenebisphosphonate (**7**) was performed to afford the intermediates **17** and **18**, respectively, in the presence of Cs_2_CO_3_ and tetrabutylammonium iodide (TBAI). The subsequent deprotection of the ethyl protecting groups using TMSBr gave the target compounds **19** and **20** in yields of 62% and 55%, respectively.

Optimization of reaction conditions. In the synthesis of target compounds, the facile connection of the phosphonate group with a heterocycle through a C-P bond is a key step, especially when a suitable linker length has to be considered in terms of the structural feature of the naturally occurring DXR-active products, FOS and FR. The most common methods for a simple C-P bond formation include the Michaelis–Arbuzov reaction and the Michaelis–Becker reaction [31,32], but both of the reactions require harsh reaction conditions, and the yields are usually low. Another important strategy to synthesize phosphonate-containing compounds involves the use of vinylphosphonate as a Michael acceptor to react with a Michael donor such as ketone, β-ketoester and Grignard reagent [33,34,35]. This type of synthesis tends to be facile and mild in reaction conditions, but the subsequent utilization of the functional groups of the Michael donors for heterocycle construction is seldom reported. In addition, the reports on the reactions of vinylphosphonate and vinylidenebisphosphonate as Michael acceptors are rather few, which is perhaps due to the relatively low reactivity in this type of Michael addition compared to the analogous vinylcarboxylate Michael acceptors. In our case, this type of Michael addition was exploited with the reactions of vinylphosphonate and vinylidenebisphosphonate with β-dicarbonyl compounds as Michael donors, which could cyclize with hydrazine and hydroxylamine to form heterocycles, as shown in Scheme 1 and Scheme 2. Hence, the optimization of the reaction conditions for the Michael addition between vinylphosphonates (**1**) and β-dicarbonyls appeared to be crucial for the target compound synthesis.

The reaction of vinylphosphonate **1** with acetoacetate as a model reaction was first optimized, and the results are shown in Table 1. When strong bases were used, such as *t*-BuOK, NaH and NaOEt, and the reaction was performed in THF or EtOH at room temperature for 6 h, the yields of the Michael addition were determined to be 60, 45 and 40%, respectively, for the isolated product **2a** (entries 1~3), and heating (entry 4) and expending the reaction time in the case of *t*-BuOK as base gave no increase in the yield. In addition, organic bases such as 1,8-diazabicyclo(5.4.0)undec-7-ene (DBU, entry 5) and 1,5-Diazabicyclo[4.3.0]non-5-ene (DBN, entry 6) gave yields of only 50% and 53%, respectively. Since these conditions gave no ideal yields, we had to seek alternative methods. It was reported that for a typical Michael addition reaction, a moderately strong base such as Cs_2_CO_3_ or K_2_CO_3_ could be beneficial with the assistance of a phase-transfer catalyst (PTC) [36]. Indeed, when TBAI or TEBAC was used as PTC and Cs_2_CO_3_ or K_2_CO_3_ was used as a base, the yield was significantly increased (entries 7~10), with the best yield of 90% for the reaction performed with TEBAC as PTC and K_2_CO_3_ as the base (entry 10). For other β-ketoesters and β-diketones, the reaction proceeded similarly and gave the corresponding intermediate **2** in yields of 87~92%. On the other hand, vinylidenebisphosphonate as the Michael acceptor tended to react faster with β-ketoesters and β-diketones using LiHMDS as a base under the reaction conditions, as indicated in Scheme 2. This higher reactivity of vinylidenebisphosphonate than vinylphosphonate is due to its higher electrophilicity caused by the double electron-withdrawing effect of the two phosphonate groups on the vinyl unit. The data of the yields of these target compounds are given in the Supplementary Materials.

### 2.2. Arabidopsis Growth Inhibitory Activity

An early report described that FOS as a DXR enzyme inhibitor can disrupt the biosynthesis of essential isoprenoids for chlorophyll and carotenoids, leading to bleaching and developmental arrest in *Arabidopsis thaliana* (Arabidopsis) [37]. Thus, when using FOS as the reference compound, the activities of the synthesized compounds against the growth of Arabidopsis were screened at an initial concentration of 100 mg/L, and the data are provided in the Supplementary Materials. For compounds with inhibition rates higher than 50% at the initial concentration of 100 mg/L, their median effective concentration (EC_50_) values were determined. In the total 32 compounds, 10 compounds were found to have inhibition rates higher than 50% against Arabidopsis at the initial concentration, with their EC_50_ values ranging from 7.8 to 88.3 mg/L, as shown in Table 2. Among them, the series **13** compounds, including **13a**, **13d**, **13e** and **13f**, performed better in inhibitory activity than FOS, and compound **13e** has the highest activity with an EC_50_ value down to 7.8 mg/L and a 3.7-fold activity of FOS, which has an EC_50_ value of 27.5 mg/L.

**Figure 3:**
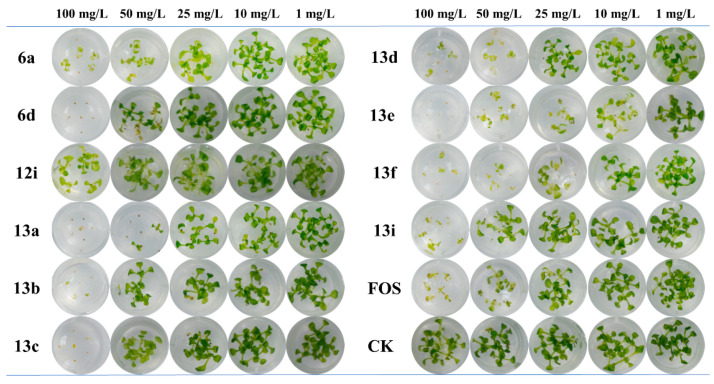
Inhibition and bleaching effects of the 10 active compounds on Arabidopsis.

The phenotype of Arabidopsis treated with FOS and the 10 compounds at concentrations of 100, 50, 25, 10 and 1 mg/L were further tested via monitoring with the digital camera, and the results are shown in Figure 3. It can be seen that FOS and all of the test compounds exhibit concentration-dependent growth inhibitions and bleaching on Arabidopsis, suggesting that the effect may arise from the inhibition on a certain pathway in the plant chloroplast, similar to FOS. At a high applied concentration of 100 mg/L, most compounds could even completely inhibit the germination of Arabidopsis. Notably, two compounds, **13e** and **13f**, had superior performances in Arabidopsis growth inhibition and bleaching effect to the control FOS at all tested concentrations.

### 2.3. Pre-Emergence Herbicidal Activity

Two model plants, dicot *Brassica napus* L. (*BN*) and monocot *Echinochloa crus-galli* (*EC*), were tested for the pre-emergence herbicidal activities of all the synthesized compounds using a standard Petri dish method [38]. The EC_50_ values collected on the inhibition of roots and stalks for the 10 active compounds, plus the 2 isoxazolinone-containing compounds, **19** and **20**, are shown in Table 3 for discussion, while the data for the other compounds are given in the Supplementary Materials. All 12 compounds in Table 3 display pre-emergence herbicidal activities, with the EC_50_ values being comparable or superior to that of FOS; compound **13e** has EC_50_ values of 10.7 and 2.3 mg/L, and compound **13f** has EC_50_ values of 17.9 and 5.8 mg/L on the *BN* root and stalk, respectively, while FOS only has the corresponding EC_50_ values of 34.7 and 32.9 mg/L. Although the two isoxazolinone-containing compounds **19** and **20** have no obvious effects on Arabidopsis, they behaved better in the inhibition of these two model plants, with the compound **20** being more powerful than FOS in the inhibitory EC_50_ value of the *BN* stalk, 8.2 vs. 32.9 mg/L.

**Figure 4:**
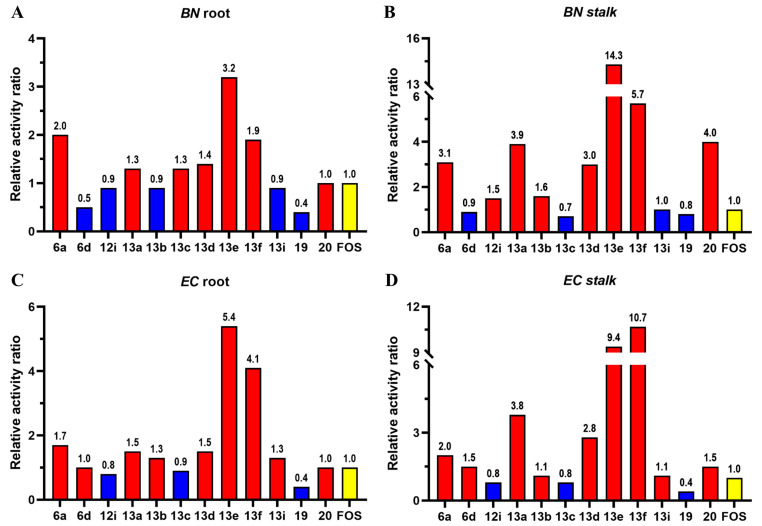
Relative activities of the 10 active compounds, plus compounds **19** and **20**, on the root and stalk of *BN* and *EC*. The red/blue bars represent relative activities higher/lower than that (yellow) of FOS, respectively.

To give a better illustration of the activity differences of the 12 compounds along with FOS, four column graphs, separately representing the four sets of relative activity data on the roots and stalks of *BN* and *EC* relative to that of FOS, are given in Figure 4. It can be seen from Figure 4A,B that compounds **6a**, **13a**, **13d~f** and **20** are more active on the roots and stalks of *BN* than FOS, which are more evident in the inhibition of the *BN* stalk, with **13e** and **13f** having 14.3- and 5.7-fold relative activities, respectively, relative to those of FOS. For the inhibition on *EC*, as shown in Figure 4C,D, most of the compounds exhibited stronger inhibitory activities than FOS. Among them, compounds **13e** and **13f** also have the strongest inhibitory effects on the roots and stalks of *EC*, with relative activities of 5.4- and 10.7-fold, respectively, with respect to those of FOS. It is also noted that both **19** and **20** have similar activities on the inhibition of the roots of *BN* and *EC* to FOS, but compound **20** has a much stronger inhibitory effect on the stalks of *BN* and *EC* than FOS, with 4.0- and 1.5-fold relative activities, respectively, compared to FOS.

### 2.4. Post-Emergence Herbicidal Activity

In terms of the results from the inhibition assays on Arabidopsis and on the pre-emergence model plants, *BN* and *EC*, six compounds, **6a**, **13a**, **13d~f** and **20**, were selected for the post-emergence herbicidal activity evaluation on representative weeds, *Amaranthus retroflexus* (*AR*) and *Echinochloa crus-galli* (*EC*). The spray dose was 300 g ai/ha, and the herbicidal effects were evaluated via visual observation [38] and fresh weighing [39], and the results are provided in Table 4. All of the test compounds, including FOS, have post-emergence inhibitory activities on the weeds. In general, all compounds behaved better on *AR* than on *EC*. Among them, **13e** showed the best herbicidal activity with the visual evaluation levels of +++ for *AR* and ++ for *EC*, which are superior to FOS, which has the levels of + for both weeds. The inhibition rates of **13e** on *AR* and *EC* determined via fresh weighing are 70.3% and 53.5%, respectively, which are 1.2- and 1.0-fold more active than FOS. In addition, compound **13f** also demonstrated similar post-emergence herbicidal activity to **13e**, and it was much better than FOS in the inhibition activity against *AR* and *EC*.

### 2.5. DXR Inhibitory Activity

After evaluating the herbicidal activity, the synthesized compounds were also tested for their inhibitory activities against recombinant *E. coli* DXR (*Ec*DXR) according to the method in the literature [40], and apreliminary screening was performed at a compound concentration of 100 μM. For compounds with an inhibition rate (InR) higher than 50%, their IC_50_ values were determined by using a series of successively decreasing concentrations. As shown in Table 5, most of the target compounds showed weak or no activity against DXR at a concentration of 100 μM, except the two 3-hydroxyisoxazole-containing bisphosphonate compounds, **13a** and **13e**, which have inhibitory rates of 60.2% and 54.9%, respectively, with IC_50_ values of 77.9 and 106.7 μM. These IC_50_ values are much larger than the IC_50_ value of FOS, 0.38 μM, a value determined in this work that is comparable to the value in the literature [40], demonstrating that **13a** and **13e** may be weak inhibitors of DXR. However, due to their high plant inhibitory effects and their low DXR activities, these compounds may have other targets involved in the inhibition process. Another possibility that cannot be excluded is that the deviations might arise from some unknown factors, such as the difference in homology between the plant-origin DXR and *Ec*DXR, and the different molecular structure parameters involved in the complex processes of absorption, distribution, metabolism, and excretion during the action.

### 2.6. DMAPP Rescue and Molecule Docking

**Figure 5:**
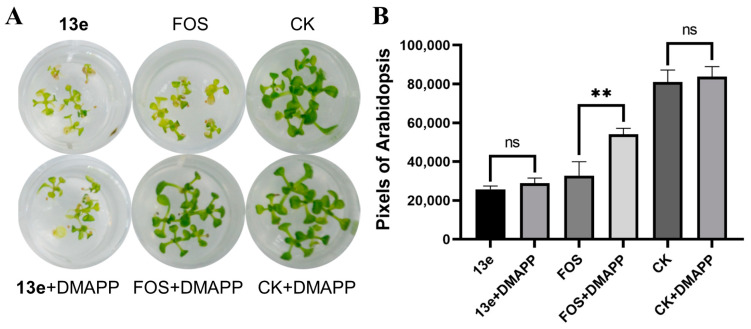
Rescue of bleaching and developmental arrest in Arabidopsis by adding exogenous DMAPP. (**A**) Images of **13e**- and FOS-treated Arabidopsis before and after rescue with DMAPP, along with the control check (CK). The concentrations of **13e** and FOS were 20 mg/L and 40 mg/L, respectively, and the DMAPP concentration was 150 mg/L. (**B**) Green channel pixel values of Arabidopsis images after different treatments. ** *p* < 0.01; ns: no significance.

DMAPP rescue. To explore whether the active compounds are acting on the DXR enzyme in the MEP pathway, a DMAPP rescue assay was conducted using the most active compound, **13e**, and the control, FOS. The principle of DMAPP rescue is that if the growth inhibition of Arabidopsis is caused by the inhibition of the DXR enzyme, adding exogenous DMAPP as an intermediate downstream of DXR should restore the growth of Arabidopsis. As shown in Figure 5, the developmental arrest and bleaching of Arabidopsis via FOS treatment were rescued with the addition of DMAPP, with the green channel pixel values of the Arabidopsis images increasing from 32,789 to 54,164, representing a 1.65-fold rescue with evident statistical significance. On the contrary, the addition of DMAPP to the compound **13e** treatment group did not show a significant increase in pixel values, indicating that **13e** may act on an herbicidal target outside of the MEP pathway.

**Figure 6:**
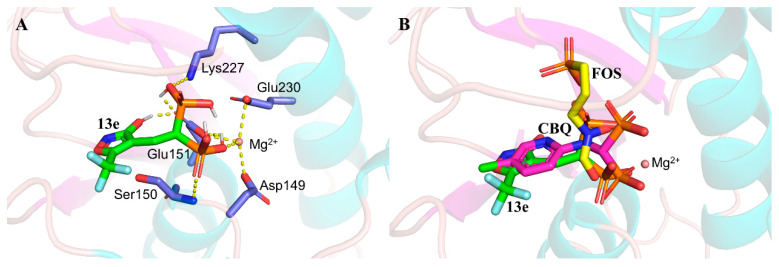
The docked conformation of **13e** with DXR enzyme and the interactions with the surrounding residues and (**A**) the conformational superimposition of **13e** with CBQ and FOS in the DXR active site (**B**). Key residues are shown as slate sticks, the hydrogen bonds and coordinate bonds are highlighted in yellow dashed lines and the Mg^2+^ ion is presented as a wheat sphere.

Molecule docking. To further assess the possibility of the mode of action of the active compounds on the target enzyme, DXR, the molecular docking of compound **13e** was performed using the Autodock Vina 1.2.3 software. Given the structural similarity between the reported pyridine-containing bisphosphonate (CBQ, shown in Figure 2A) and **13e**, the complex crystal structure of CBQ with *Ec*DXR (PDB ID: 1T1R) was chosen as a template for docking [23]. The interactions between compound **13e** and the surrounding residues of the DXR active site are depicted in Figure 6A. The bisphosphonate group of **13e** forms hydrogen bonding with Ser150, Glu151 and Lys227 within the bond length range of <3.0 Å, while the 3-hydroxyisoxazole also interacts with Glu151 to form an additional hydrogen bond. In addition, **13e** demonstrates a binding mode to Mg^2+^ in the DXR active site, similar to that of CBQ, through the coordination of one hydroxyl oxygen of the bisphosphonate group. In Figure 6B, the binding conformations of **13e** and CBQ could be superimposed well, with the bisphosphonate group being located in the Mg^2+^ binding site, and the heterocycle part occupying the hydrophobic cavity, both of which are different from FOS, which uses its hydroxamate group to bind with the metal enzyme. It is worthy to mention that **13e** and CBQ have comparable relative activities with respect to FOS in DXR inhibition, but with much inferior IC_50_ values as compared to those of FOS [23,40]. This tendency to give low inhibition effects for **13e** and CBQ may arise from the fact that their binding conformations are reversed compared to that of FOS, which uses the hydroxamate moiety to coordinate with the metal ion and uses the monophosphonate to bind with the surrounding residues at the active site of the DXR enzyme.

### 2.7. Discussion on Structure–Activity Relationships

In this work, a bioactivity evaluation of synthesized compounds was performed on Arabidopsis, pre-emergency model plants, *Brassica napus* L. and *Echinochloa crus-galli*, post-emergency plants, *Amaranthus retroflexus* and *Echinochloa crus-galli*, and the recombinant DXR enzyme. Several compounds demonstrated better herbicidal activities compared to FOS, although they might have a different target involved in the inhibiting process. The inhibition phenotype analyses revealed that the molecular structure of the synthesized compounds has a significant influence on the inhibition of different plants, and in Arabidopsis, the structure–activity relationship is of representativeness.

In terms of the molecular structure and the Arabidopsis inhibitory activity, several trends could be derived. One is from the influence of the number of phosphate fragments, and bisphosphonates tended to have higher herbicidal activities on Arabidopsis than the monophosphonates, as illustrated in the EC_50_ ratios for the two bis-/monophosphonate pairs, **13a**/**6a** and **13d/6d**, which were 21.6/28.6 mg/L and 23.1/45.7 mg/L, respectively. The second trend comes from the influence of the heterocycle unit. For example, the 3-hydroxyisoxazole-containing monophosphonates **6a** and **6d** displayed moderate inhibitory activities with EC_50_ values of 28.6 and 45.7 mg/L, while other heterocycle-containing monophosphonates gave low or even no inhibition effects. The 3-hydroxyisoxazole-containing bisphosphonates **13a~f** performed even better, showing moderate to good activity with EC_50_ values from 7.8 to 48.4 mg/L. The third trend is from the influence of fluorine-containing substituent, and -CF3 (for **13e**) and -CHF_2_ (for **13f**) on 3-hydroxyisoxazole have the highest activities, with EC_50_ values as low as 7.8 and 8.7 mg/L, respectively, outweighing all other compounds.

Finally, compounds **19** and **20**, which contain the isoxazolinone fragment like the active component of the commercial herbicide clomazone, only exhibited a weak inhibitory effect on Arabidopsis. Clomazone targets the first enzyme DXS in the MEP pathway as a type of propesticide, which is activated by metabolic oxidation in the plant to form ketoclomazone [26]. It is speculated that compounds **19** and **20** are unable to undergo this kind of metabolism in the plant, thereby giving no activation to display an inhibitory role.

The structure–activity relationship summarized on the Arabidopsis inhibition is also applicable to that of the inhibition on other model plants. For example, 3-hydroxyisoxazole-containing **6a** and **13a** demonstrated good inhibitory activity not only on Arabidopsis but also on other tested model plants; introducing -CF_3_ and -CHF_2_ onto the 3-hydroxyisoxazole further improved the herbicidal activity on all plants, especially in the case of compound **13e**, which has one -CF_3_ group on 3-hydroxyisoxazole and displayed the best activity among all the synthesized compounds.

## 3. Materials and Methods

### 3.1. Instruments and Reagents

^1^H NMR, ^13^C NMR and ^31^P NMR spectra were recorded on a Varian Mercury-Plus 400 MHz spectrometer. HRMS were acquired using an Agilent 6224 TOF LC/MS instrument. All reagents and solvents used were of analytical or chemical purity. FOS was synthesized with reference to the method reported by our lab [41].

### 3.2. Synthesis

The detailed synthesis procedures and characterization data of target compounds are given in the Supplementary Materials.

### 3.3. Biological Assays

#### 3.3.1. Arabidopsis Growth Inhibition Assay

The Arabidopsis inhibitory activity was evaluated using 24-well plates [42], and the specific cultivation conditions are given in the Supplementary Materials. After 10 days of cultivation, the plates were photographed with a digital camera, and the Adobe Photoshop 2020 software was used to determine the green channel pixel value of the Arabidopsis image in each well. The inhibition rate was calculated by comparing the pixel values of the compound treatment group to that of the blank group. The initial test concentration was 100 mg/L, and for compounds with inhibition rates higher than 50%, concentrations of 50, 25, 10 and 1 mg/L were further tested to calculate their EC_50_ values.

#### 3.3.2. Pre-Emergence Herbicidal Inhibition Assay

The pre-emergence activity of the compounds against monocot *Echinochloa crus-galli* (*EC*) and dicot *Brassica napus* L. (*BN*) was evaluated using the standard Petri dish test [38]. The compounds were dissolved in DMF, emulsified with Tween-80 and diluted with water to form stock solutions, each of which was further diluted to a gradient of 100, 50, 25, 10 and 1 mg/L for test. The test solutions were added to Petri dishes lined with a filter paper, on which 10 seeds of *EC* and *BN* were placed. After cultured in an intelligent climate chamber with a humidity of 65% at 26 °C for 7 days, the corresponding EC_50_ values were calculated.

#### 3.3.3. Post-Emergence Herbicidal Inhibition Assay

The post-emergence activity of compounds against *EC* and *Amaranthus retroflexus* (*AR*) was determined at a dose of 300 g ai/ha. The model plants initially grow as seedlings, and a few days prior to treatment, five seedlings with similar growth conditions for the plant were transplanted into one pot containing nutrient soil. The test solutions were prepared by dissolving the compounds in water containing DMF and Tween-80 and sprayed when the plant grew to the 1~2 leaf stage. The herbicidal activity was evaluated via visual observation [38] and fresh weighing [39] after two weeks of treatment.

#### 3.3.4. DXR Enzyme Inhibition Assay

The cloning and expression of *Ec*DXR were performed using a previously reported method [40], and the enzyme inhibition activity was evaluated by monitoring the oxidation of NADPH in the enzyme catalysis process using a microplate reader. The detailed protocol is given in Supplementary Materials.

#### 3.3.5. DMAPP Rescue

The DMAPP rescue on Arabidopsis was conducted using the afore-mentioned culture conditions. The Arabidopsis seeds were divided into three groups labeled as FOS, **13e** and blank control, and the test concentrations of FOS and **13e** were 40 and 20 mg/L, respectively. To one of the two species in each group, an additional 150 μg of DMAPP (Aladdin) was added. After 10 days of cultivation, the green channel pixel values of Arabidopsis in each well were measured. A two-tailed paired Student’s *t*-test was used to compare the two species within the same group, and *p* < 0.05 indicates a statistically significant difference.

### 3.4. Molecular Docking

The co-crystal structure of *Ec*DXR with bisphosphonate compound (PDB ID: 1T1S) was used as the docking template, and the molecular docking of **13e** to the *Ec*DXR active site was performed using Autodock vina 1.2.3 software. The docking employed the semi-flexible docking mode of the Vina force field, with the coordinate values of Mg^2+^ as the docking center. According to the docking affinity data and by visually inspecting the docking conformations using the PyMOL 2.5.6 software, the best binding conformation of **13e** was determined and superimposed with that of CBQ and FOS in the *Ec*DXR active site for comparative analysis.

## 4. Conclusions

In this work, heterocycle-containing mono- and bisphosphonic acid compounds as FOS analogs were designed by replacing the hydroxamate unit of FOS with various heterocycles while retaining the monophosphonic acid in FOS or replacing it with a bisphosphonic acid group. These compounds were facilely synthesized with the key steps including the Michael addition of diethyl vinylphosphonate or tetraethyl vinylidenebisphosphonate with β-dicarbonyls, and the subsequent cyclic condensation with hydrazine or hydroxylamine. Two additional isoxazolinone-bearing FOS analogs were also synthesized via the Michaelis–Becker reaction as a key step. With the optimization of the key steps, the target compounds were obtained in high yields. The bioactivity evaluation on Arabidopsis and the pre- and post-emergence herbicidal tests on the model plants revealed that some of the compounds have activities that are higher or comparable to that of FOS, and one compound, namely **13e**, was identified to have the best activity, for example, on Arabidopsis, with a 3.7-fold inhibitory activity enhancement compared to the control FOS. The molecule docking suggested that **13e** could interact with the active site of DXR in a different binding manner from FOS, while the DMAPP rescue assay failed to indicate that the DXR enzyme is the target of **13e**, implying that a different target might be involved in the inhibiting process. With the finding of valuable compounds with activities higher than FOS and with the established facile synthesis method, though, this work demonstrated a valuable strategy of bioisosteric replacement to discover new FOS analogs with high herbicidal activities.

## Figures and Tables

**Table 1:** Optimization of the reaction conditions for Michael addition ^a^.

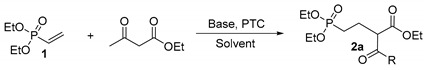					
**Entry**	**Base**	**PTC**	**Solvent**	**Temp (°C)**	**Yield (%) ^b^**
1	*t*-BuOK	--	THF	r.t.	60
2	NaH	--	THF	r.t.	45
3	NaOEt	--	EtOH	r.t.	40
4	*t*-BuOK	--	THF	66	62
5	DBU	--	THF	r.t.	50
6	DBN	--	THF	r.t.	53
7	Cs_2_CO_3_	TBAI	CH_3_CN	80	80
8	Cs_2_CO_3_	TEBAC	CH_3_CN	80	85
9	K_2_CO_3_	TBAI	CH_3_CN	80	83
10	K_2_CO_3_	TEBAC	CH_3_CN	80	90

^a^ Reaction conditions: **1** (1 mmol), ethyl acetoacetate (1 mmol), base (1 mmol), PTC (0.1 mmol), solvent (5 mL), 6 h. ^b^ Yield of isolated product.

**Table 2:** Median effective concentrations of target compounds against Arabidopsis growth.

Comp	EC_50_ (mg/L) ^a^	Comp	EC_50_ (mg/L)	Comp	EC_50_ (mg/L)
**5a**	>100	**6f**	>100	**13b**	37.7 ± 2.8
**5b**	>100	**12a**	>100	**13c**	48.4 ± 5.3
**5c**	>100	**12b**	>100	**13d**	23.1 ± 1.9
**5d**	>100	**12c**	>100	**13e**	7.8 ± 1.2
**5e**	>100	**12d**	>100	**13f**	8.7 ± 1.3
**5f**	>100	**12e**	>100	**13g**	>100
**6a**	28.6 ± 3.9	**12f**	>100	**13h**	>100
**6b**	>100	**12g**	>100	**13i**	40.7 ± 2.9
**6c**	>100	**12h**	>100	**19**	>100
**6d**	45.7 ± 4.8	**12i**	88.3 ± 4.3	**20**	>100
**6e**	>100	**13a**	21.6 ± 3.8	**FOS**	27.5 ± 3.1

^a^ EC_50_ values of inhibition on Arabidopsis are presented as mean ± SD.

**Table 3:** Pre-emergence herbicidal activities of the 10 active compounds plus the compounds **19** and **20** on the roots and stalks of *BN* and *EC* ^a^.

Comp	EC_50_ (mg/L)			
	*BN*		*EC*	
	Root	Stalk	Root	Stalk
**6a**	17.3	10.5	24.0	18.9
**6d**	67.5	38.2	40.1	25.7
**12i**	36.6	21.7	53.1	47.7
**13a**	26.9	8.5	27.1	10.2
**13b**	38.8	20.2	29.9	35.3
**13c**	27.7	46.9	43.9	45.8
**13d**	25.2	11.0	27.2	13.5
**13e**	10.7	2.3	7.4	4.1
**13f**	17.9	5.8	9.7	3.6
**13i**	40.5	33.5	32.1	34.6
**19**	79.3	39.2	>100	88.0
**20**	34.6	8.2	40.0	25.2
**FOS**	34.7	32.9	40.2	38.4

^a^ *BN*, *Brassica napus* L.; *EC, Echinochloa crus-galli*.

**Table 4:** Post-emergence herbicidal activities of the 6 selected compounds against *AR* and *EC* ^a^.

Comp	Visual Evaluation ^b^		Loss of Weight (%)	
	*AR*	*EC*	*AR*	*EC*
**6a**	+	+	35.6	30.8
**13a**	++	+	52.3	35.8
**13d**	++	+	45.7	34.9
**13e**	+++	++	70.3	53.5
**13f**	+++	++	61.8	48.1
**20**	+	+	23.6	22.9
FOS	+	+	31.5	27.4

^a^ *AR*, *Amaranthus retroflexus*; *EC*, *Echinochloa crus-galli*. ^b^ Activity grade (percentage of inhibition): “++++“: ≥80%; “+++”: ≥60%; “++”: ≥40%; “+”: ≥20%; “−”: <20%.

**Table 5:** Inhibition activities of all the synthesized compounds on DXR enzyme.

Comp	InR (%) ^a^	Comp	InR (%)	Comp	InR (%)
**5a**	11.9	**6f**	11.7	**13b**	27.4
**5b**	12.9	**12a**	13.7	**13c**	28.4
**5c**	6.1	**12b**	21.9	**13d**	14.0
**5d**	17.7	**12c**	32.3	**13e**	54.9
**5e**	23.0	**12d**	12.4	**13f**	24.4
**5f**	32.4	**12e**	22.8	**13g**	7.3
**6a**	15.8	**12f**	6.1	**13h**	10.3
**6b**	19.5	**12g**	11.6	**13i**	30.3
**6c**	7.5	**12h**	2.3	**19**	22.5
**6d**	17.3	**12i**	27.1	**20**	29.5
**6e**	12.1	**13a**	60.2	FOS	98.7

^a^ InR value was determined by measuring the DXR activity at a compound concentration of 100 μM.

## Data Availability

Data are contained within the article and Supplementary Materials.

## Supplementary Materials

The following supporting information can be downloaded at: https://www.mdpi.com/article/10.3390/molecules28227509/s1. Detailed information of the synthesis procedures and the structural characterization data for target compounds, the inhibition test methods for Arabidopsis and the DXR enzyme and the complete results of the inhibition activities on Arabidopsis and pre-emergency model plants.
