# Supplementary material for: Design, Synthesis and Bioactivity Evaluation of Heterocycle-Containing Mono- and Bisphosphonic Acid Compounds
Source: Molecules. 2023 Nov 9;28(22):7509. doi: 10.3390/molecules28227509 (PMC10673511; doi:10.3390/molecules28227509)
Supplement: Supplementary file 1 [file molecules-28-07509-s001.zip › molecules-2659271-supplementary.pdf]

Supporting Information

Design, Synthesis and Bioactivity Evaluation of Heterocycle-Containing Mono- and Bisphosphonic Acid Compounds

Table of Contents

1. Inhibition rates of target compounds against Arabidopsis…………………………………....S2
2. EC_{50} values of target compounds against model plants *BN* and *EC*…..………………………….S3
3. Synthesis procedures……………………………………………………………………….S4
4. Biological assays…………………………………………………………………………...S7
5. Chemical structures, physical properties, and the spectral data of the target compounds……...S8
6. Spectra of the target compounds…………………………………………………………..S14

1. Inhibition rates of target compounds against Arabidopsis

Table S1. Inhibition rates of target compounds against Arabidopsis

Comp	InR (%)[a]	Comp	InR (%)	Comp	InR (%)
**5a**	24.0	**6f**	13.4	**13b**	100
**5b**	26.5	**12a**	47.2	**13c**	100
**5c**	43.6	**12b**	29.6	**13d**	93.2
**5d**	48.3	**12c**	49.7	**13e**	100
**5e**	15.8	**12d**	44.8	**13f**	95.4
**5f**	37.7	**12e**	31.5	**13g**	39.7
**6a**	90.4	**12f**	47.0	**13h**	43.2
**6b**	46.5	**12g**	40.4	**13i**	90.1
**6c**	42.0	**12h**	41.2	**19**	43.2
**6d**	100	**12i**	55.8	**20**	47.9
**6e**	12.3	**13a**	100	**FOS**	86.8

[a] Inhibition rates were determined at a compound concentration of 100 mg/L.

2. EC_{50} values of target compounds against model plants *BN* and *EC*

Table S2. EC_{50} values of target compounds against model plants *BN* and *EC*[a]

Comp	EC_{50} (mg/L)			
	BN		*EC*	
	root	stalk	root	stalk
**5a**	>100	32.8	54.1	68.5
**5b**	>100	>100	86.7	92.2
**5c**	>100	48.2	76.3	10.6
**5d**	>100	96.9	32.1	>100
**5e**	63.0	>100	>100	59.6
**5f**	>100	>100	71.6	31.4
**6a**	17.3	10.5	24.0	18.9
**6b**	70.1	65.8	24.7	36.1
**6c**	99.7	33.7	65.4	41.1
**6d**	67.5	38.2	40.1	25.7
**6e**	>100	68.4	>100	54.1
**6f**	>100	>100	>100	41.7
**12a**	51.3	87.1	>100	47.7
**12b**	61.6	24.5	77.5	55.3
**12c**	55.1	33.1	68.3	36.4
**12d**	21.2	41.5	>100	39.1
**12e**	>100	45.7	46.8	>100
**12f**	15.6	22.3	36.5	12.6
**12g**	>100	32.9	38.8	31.4
**12h**	40.1	22.3	44.2	31.1
**12i**	36.6	21.7	53.1	47.7
**13a**	26.9	8.5	27.1	10.2
**13b**	38.8	20.2	29.9	35.3
**13c**	27.7	46.9	43.9	45.8
**13d**	25.2	11.0	27.2	13.5
**13e**	10.7	2.3	7.4	4.1
**13f**	17.9	5.8	9.7	3.6
**13g**	60.4	41.5	79.8	32.3
**13h**	39.2	50.8	48.0	45.7
**13i**	40.5	33.5	32.1	34.6
**19**	79.3	39.2	>100	88.0
**20**	34.6	8.2	40.0	25.2
**FOS**	34.7	32.9	40.2	38.4

[a] *BN*, *Brassica napus L.*; *EC*, *Echinochloa crus-galli.*

3. Synthesis procedures

*3.1. Synthesis of compounds **2**.* Diethyl vinylphosphonate **1** (10 mmol, 1.64 g), β-dicarbonyls (10 mmol), K_2CO_3 (10 mmol, 1.38 g), and TEBAC (1 mmol, 228 mg) were dissolved in CH_3CN (50 mL) and stirred under reflux for 6 h. After cooling to room temperature, saturated NaCl aqueous solution (30 mL) was added and the solution was extracted with CH_2Cl_2. The organic phase was dried over Na_2SO_4, filtered, concentrated, and passed through a chromatographic column (petroleum ether: ethyl acetate = 1:1, v/v) to give compounds **2a~f** in 82~95% yields.

*3.2. Synthesis of compounds **3** and **10**.* To a solution of **2** (5 mmol) in EtOH (25 mL), hydrazine hydrate (6 mmol, 300 mg) was added dropwise, and the reaction was stirred under reflux for 3 h. After cooling to room temperature, the solvent was removed using rotary evaporator, and the residue was purified by column chromatography (CH_2Cl_2: MeOH = 50:1, v/v) to afford compounds **3a~f** in 90~96% yields. For the synthesis of compounds **10a~i**, intermediate **9** was used, affording the cyclization products in 92~95% yields.

*3.3. Synthesis of compounds **4** and **11**.* Hydroxylamine hydrochloride (6 mmol, 420 mg) and K_2CO_3 (6 mmol, 0.83 g) were added to a solution of **2** (5 mmol) in EtOH (25 mL), and the reaction was stirred under reflux for 3 h. After cooling to room temperature, the solvent was removed by rotary evaporator, and the residue was purified by column chromatography (CH_2Cl_2: MeOH = 50:1, v/v) to afford compounds **4a~f** in 85~92% yields. For the synthesis of compounds **11a~i**, intermediate **9** was used, affording the cyclization products in 88~95% yields.

*3.4. Synthesis of compound **8**.* Tetraethyl methylenediphosphonate **7** (14 mmol, 4.0 g), paraformaldehyde (70 mmol, 2.1 g), and diethylamine (14 mmol, 1.02 g) were dissolved in methanol (50 mL) and stirred under reflux for 24 h. The reaction mixture was cooled to room temperature, concentrated, and the residue was dissolved in toluene (80 mL). A catalytic amount of TsOH (20 mg) was added, and the mixture was stirred at 110°C for another 24 h. After cooling to room temperature, saturated NaCl aqueous solution (30 mL) was added and the solution was extracted with toluene. The combined organic phase was dried over Na_2SO_4 and concentrated to provide compound **8** (95% yield), which could be used in the following reaction without column chromatography.

*3.5. Synthesis of compounds **9**.* LiHDMS (1 mmol, 1 mL) was added dropwise to a solution of β-dicarbonyls (10 mmol) in THF (50 mL) at 0°C, and tetraethyl VBP **8** (10 mmol, 3.0 g) was added

after 20 min. The reaction mixture was stirred at room temperature for 6 h, and saturated NH_4Cl aqueous solution (20 mL) was added to quench the reaction, followed by extraction with CH_2Cl_2. The organic phase was dried over Na_2SO_4, filtered, concentrated, and passed through a chromatographic column (CH_2Cl_2: MeOH = 50:1, v/v) to give compounds **9a~i** in 87~96% yields.

*3.6. Synthesis of compound **15**.* To a solution of hydroxylamine hydrochloride (0.15 mol, 10.4 g) in H_2O (60 mL) at 0°C, 3-chloropivaloyl chloride **14** (0.12 mol, 15.2 g) were added dropwise and 30% NaOH solution was added simultaneously to adjust the solution to pH = 7.2. After stirred at room temperature for 5 h, the suspension was filtered and the residue was dissolved in H_2O (50 mL), and 30% NaOH solution was added dropwise to adjust the solution to pH = 9.2 with magnetic stirring. After 12 h, ethyl acetate (20 mL) was added to the reaction mixture, and the aqueous phase was adjusted to pH 1~2 with 1M HCl before extraction. Then, the solution was extracted with ethyl acetate, and the organic phase was combined, dried over Na_2SO_4, and concentrated to provide compound **15** in 86% yield, which could be used in the following reaction without column chromatography.

*3.7. Synthesis of compound **16**.* NaH (12 mmol, 0.48 g) was added to a solution of **15** (10 mmol, 0.87 g) in anhydrous DMF (50mL) at 0°C with magnetic stirring. After 1 h, 1,3-dibromopropane (10 mmol, 2 g) was added, and the reaction was continued for 12 h at room temperature. H_2O (30mL) was added and the mixture was extracted with ethyl acetate. The organic phase was dried over Na_2SO_4, filtered, concentrated, and passed through a chromatographic column (petroleum ether: ethyl acetate = 10:1, v/v) to give compound **26** in 90% yield.

*3.8. Synthesis of compounds **17** and **18**.* To a suspension of Cs_2CO_3 (10 mmol, 3.2 g) and TBAI (5 mmol, 1.85 g) in 60mL DMF was added dropwise diethyl phosphite (5 mmol, 0.69 g) and the solution was magnetically stirred for 1 h at room temperature. Then **16** (5 mmol, 1.04 g) in DMF (3 mL) was added dropwise to the solution and the reaction was maintained for 24 h. H_2O (30 mL) was added and the solution was extracted with ethyl acetate. The organic phase was dried over Na_2SO4, filtered, concentrated, and passed through a chromatographic column (petroleum ether: ethyl acetate = 2:1, v/v) to afford compound **17** in 78% yield. Compound **18** were obtained similarly in yield of 86%.

*3.9. Synthesis of target compounds **5~6**, **12~13**, **19**, and **20**.* To a solution of monophosphonates (1 mmol) in anhydrous CH_2Cl_2, TMSBr (5 mmol, 765 mg) was added dropwise at 0°C, and the

reaction mixture was stirred at room temperature for 24 h. Then, the solvent was removed by rotary evaporator and a mixed solvent (10 mL, THF: H_2O = 9:1, v/v) was added with magnetic stirring. After 1 h, CH_2Cl_2 (5 mL) and H_2O (5 mL) was added and the solution was extracted with CH_2Cl_2. The aqueous phase was concentrated and dried in vacuum to afford the monophosphonate acid target compounds **5~6** and **19** in 92~98% yields. For the synthesis of the bisphosphonate acid target compounds **12~13** and **20**, 10 equivalents of TMSBr was used, affording the target products in 88~95% yields.

4. Biological assays

4.1. Arabidopsis inhibition assay. First, arabidopsis seeds were soaked in 75% ethanol for 30 s and washed three times in sterile water, followed by aspiration onto filter paper for drying in air. Then, 0.9 mL of warm growth medium (0.62 g 1/2 MS medium, 3 mg $FeSO_4$-$7H_2O$, 2.5 g sucrose, 0.125 g 2-morpholineethanesulfonic acid, and 2.3 g agar per 250 mL of growth medium at pH = 5.8) and 0.1 mL of the appropriate concentration of compound were added to each well. After solidification of the growth medium, the seeds were placed on the medium surface with 6 seeds per well. The plates were sealed with parafilm and stored in a refrigerator at 4 °C for 2 days, after which they were transferred to an intelligent climate chamber (RXZ-380C, Ningbo, China) under growth conditions of 25 °C, 75 % humidity, 16 h of light and 8 h of darkness. After 10 days of cultivation, the plates were photographed with a digital camera, and Adobe Photoshop software was used to determine the green channel pixel value of arabidopsis image in each well. The inhibition efficiency (IE) was calculated by comparing the pixel values of the compound treatment group to that of the blank group. The initial test concentration was 100 mg/L, and for compounds with inhibition rates higher than 50%, the concentrations of 50, 25, 10 and 1 mg/L were further tested, and the corresponding EC_{50} values were determined.

4.2. DXR enzyme inhibition assay. The cloning and expression of *Ec*DXR was performed as previously reported, and the enzyme inhibition activity was assessed by monitoring the oxidation of NADPH using the microplate reader. Briefly, the total volume of the assay solution was 105 μL, containing 100 mM Tris-HCl (pH = 7.5), 2 mM $MgCl_2$, 0.2 mM NADPH, 0.2 mM DXP, and 1 μL compound solution at an appropriate concentration. The reaction was initiated by adding 0.0003 U of recombinant *Ec*DXR and the inhibition rate of the compound was calculated by monitoring the absorbance change at 340 nm at 37 °C by the Multiskan FC Microplate Reader. An initial screening was performed at a compound concentration of 100 μM, and if the inhibition rate was greater than 50%, the inhibition rates of the compound over a range of concentrations were determined and the corresponding IC_{50} value calculated.

5. Chemical structures, physical properties, and the spectral data of the target compounds

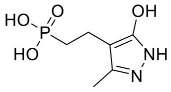

(2-(5-hydroxy-3-methyl-1H-pyrazol-4-yl)ethyl)phosphonic acid (**5a**)

Yield: 80%, white solid, m.p. 220−221 ℃. ^{1}H NMR (400 MHz, DMSO-d_6) δ 8.53 (br, 2H), 2.44–2.38 (m, 2H), 2.05 (s, 3H), 1.67 (q, J = 12.8 Hz, 2H). ^{13}C NMR (100 MHz, DMSO-d_6) δ 159.6, 138.0, 101.2, 28.7 (d, J = 137.6 Hz), 15.6, 10.0. ^{31}P NMR (162 MHz, DMSO-d_6) δ 26.20. HRMS m/z calcd for $C_6H_{11}N_2O_4P$ [M - H]$^-$ 205.0384, found 205.0387.

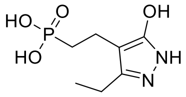

(2-(3-ethyl-5-hydroxy-1H-pyrazol-4-yl)ethyl)phosphonic acid (**5b**)

Yield: 75%, white solid, m.p. 217−218 ℃. ^{1}H NMR (400 MHz, DMSO-d_6) δ 10.41 (br, 2H), 2.47–2.39 (m, 4H), 1.70–1.62 (m, 2H), 1.11 (t, J = 7.6 Hz, 3H). ^{13}C NMR (100 MHz, DMSO-d_6) δ 159.6, 143.2, 100.4, 28.9 (d, J = 132.8 Hz), 17.9, 15.4, 13.6. ^{31}P NMR (162 MHz, DMSO-d_6) δ 26.24. HRMS m/z calcd for $C_7H_{13}N_2O_4P$ [M - H]$^-$ 219.0540, found 219.0541.

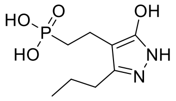

(2-(5-hydroxy-3-propyl-1H-pyrazol-4-yl)ethyl)phosphonic acid (**5c**)

Yield: 73%, white solid, m.p. 228−229 ℃. ^{1}H NMR (400 MHz, DMSO-d_6) δ 8.84 (br, 2H), 2.41–2.37 (m, 4H), 1.72–1.59 (m, 2H), 1.52–1.48 (m, 2H), 0.84 (t, J = 7.2 Hz, 3H). ^{13}C NMR (100 MHz, DMSO-d_6) δ 159.5, 142.0, 101.0, 28.8 (d, J = 134.2 Hz), 26.4, 22.0, 15.5, 13.7. ^{31}P NMR (162 MHz, DMSO-d_6) δ 26.13. HRMS m/z calcd for $C_8H_{15}N_2O_4P$ [M - H]$^-$ 233.0697, found 233.0695.

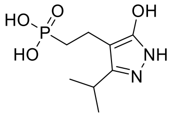

(2-(5-hydroxy-3-isopropyl-1H-pyrazol-4-yl)ethyl)phosphonic acid (**5d**)

Yield: 75%, white solid, m.p. 210−211 ℃. ^{1}H NMR (400 MHz, DMSO-d_6) δ 10.42 (br, 2H), 2.93–2.82 (m, 1H), 2.58–2.38 (m, 2H), 1.74–1.59 (m, 2H), 1.16 (s, 6H). ^{13}C NMR (100 MHz, DMSO-d_6) δ 159.6, 147.1, 99.6, 29.0 (d, J = 132.8 Hz), 24.9, 21.9, 15.5. ^{31}P NMR (162 MHz, DMSO-d_6) δ 26.26. HRMS m/z calcd for $C_8H_{15}N_2O_4P$ [M - H]$^-$ 233.0697, found 233.0696.

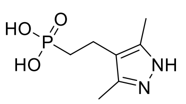

(2-(3,5-dimethyl-1H-pyrazol-4-yl)ethyl)phosphonic acid (**5e**)

Yield: 82%, white solid, m.p. >250 ℃. ^{1}H NMR (400 MHz, D_2O) δ 2.49–2.43 (m, 2H), 2.06 (s, 6H), 1.60–1.52 (m, 2H). ^{13}C NMR (100 MHz, D_2O) δ 143.1, 115.7 (d, J = 19.6 Hz), 29.0 (d, J = 129.8 Hz), 16.6 (d, J = 4.2 Hz), 9.42. ^{31}P NMR (162 MHz, D_2O) δ 24.20. HRMS m/z calcd for $C_7H_{13}N_2O_3P$ [M - H]$^-$ 203.0591, found 203.0590.

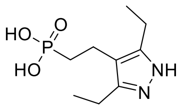

(2-(3,5-diethyl-1H-pyrazol-4-yl)ethyl)phosphonic acid (**5f**)

Yield: 78%, white solid, m.p. >250 ℃. ^{1}H NMR (400 MHz, D_2O) δ 2.61 (q, *J* = 7.6 Hz, 4H), 2.57–2.55 (m, 2H), 1.83–1.75 (m, 2H), 1.08 (t, *J* = 7.6 Hz, 3H). ^{13}C NMR (100 MHz, D_2O) δ 148.5, 115.7 (d, *J* = 16.6 Hz), 26.7 (d, *J* = 134.0 Hz), 17.2, 14.9 (d, *J* = 3.8 Hz), 11.5. ^{31}P NMR (162 MHz, D_2O) δ 28.19. HRMS m/z calcd for $C_9H_{17}N_2O_3P$ [M - H]$^-$ 231.0904, found 231.0901.

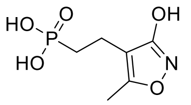

(2-(3-hydroxy-5-methylisoxazol-4-yl)ethyl)phosphonic acid (**6a**)

Yield: 75%, colorless oil. ^{1}H NMR (400 MHz, CD_3OD) δ 2.49–2.45 (m, 2H), 2.17 (s, 3H), 1.97–1.91 (m, 2H). ^{13}C NMR (100 MHz, CD_3OD) δ 174.9, 162.7, 97.5 (d, *J* = 16.2 Hz), 26.1 (d, *J* = 137.0 Hz), 15.8, 10.2. ^{31}P NMR (162 MHz, CD_3OD) δ 29.66. HRMS m/z calcd for $C_6H_{10}NO_5P$ [M - H]$^-$ 206.0224, found 206.0226.

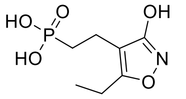

(2-(5-ethyl-3-hydroxyisoxazol-4-yl)ethyl)phosphonic acid (**6b**)

Yield: 73%, colorless oil. ^{1}H NMR (400 MHz, CD_3OD) δ 2.59 (q, *J* = 7.6 Hz, 2H), 2.51–2.44 (m, 2H), 1.99–1.90 (m, 2H), 1.20 (t, *J* = 7.6 Hz, 3H). ^{13}C NMR (100 MHz, CD_3OD) δ 173.8, 166.0, 95.2 (d, *J* = 17.2 Hz), 25.2 (d, *J* = 136.4 Hz), 18.2, 14.7, 10.7. ^{31}P NMR (162 MHz, CD_3OD) δ 29.71. HRMS m/z calcd for $C_7H_{12}NO_5P$ [M - H]$^-$ 220.0380, found 220.0382.

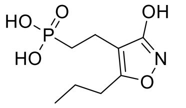

(2-(3-hydroxy-5-propylisoxazol-4-yl)ethyl)phosphonic acid (**6c**)

Yield: 70%, colorless oil. ^{1}H NMR (400 MHz, D_2O) δ 2.45 (t, *J* = 8.0 Hz, 2H), 2.37–2.33 (m, 2H), 1.86–1.80 (m, 2H), 1.48 (q, *J* = 7.6 Hz, 2H), 0.79 (t, *J* = 7.6 Hz, 3H). ^{13}C NMR (100 MHz, D_2O) δ 174.9, 165.6, 95.9 (d, *J* = 17.0 Hz), 26.2, 25.0 (d, *J* = 134.8 Hz), 20.0, 14.2, 12.6. ^{31}P NMR (162 MHz, D_2O) δ 29.76. HRMS m/z calcd for $C_8H_{14}NO_5P$ [M - H]$^-$ 234.0537, found 234.0540.

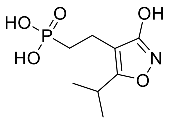

(2-(3-hydroxy-5-isopropylisoxazol-4-yl)ethyl)phosphonic acid (**6d**)

Yield: 77%, colorless oil. ^{1}H NMR (400 MHz, D_2O) δ 2.99–2.92 (m, 1H), 2.37–2.30 (m, 2H), 1.85–1.77 (m, 2H), 1.06 (d, *J* = 7.2 Hz, 6H). ^{13}C NMR (100 MHz, D_2O) δ 175.0, 170.3, 94.7 (d, *J* = 16.8 Hz), 25.7, 24.5, 19.4, 14.3 (d, *J* = 3.8 Hz). ^{31}P NMR (162 MHz, D_2O) δ 29.76. HRMS m/z calcd for $C_8H_{14}NO_5P$ [M - H]$^-$ 234.0537, found 234.0538.

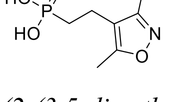

(2-(3,5-dimethylisoxazol-4-yl)ethyl)phosphonic acid (**6e**)

Yield: 77%, white solid, m.p. >250 ℃. ^{1}H NMR (400 MHz, CD_3OD) δ 2.65–2.60 (m, 2H), 2.30 (s, 3H), 2.18 (s, 3H), 1.91–1.83 (m, 2H). ^{13}C NMR (100 MHz, CD_3OD) δ 165.6, 159.5, 113.0 (d, *J* = 15.6 Hz), 26.4 (d, *J* = 137.0 Hz), 15.1 (d, *J* = 4.2 Hz), 9.5, 8.7. ^{31}P NMR (162 MHz, CD_3OD) δ

28.62. HRMS m/z calcd for $C_7H_{12}NO_4P$ [M - H]$^-$ 204.0431, found 204.0429.

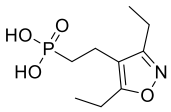

(2-(3,5-diethylisoxazol-4-yl)ethyl)phosphonic acid (**6f**)

Yield: 75%, white solid, m.p. >250 ℃. ^{1}H NMR (400 MHz, CD_3OD) δ 2.71 (q, *J* = 7.6 Hz, 2H), 2.62 (q, *J* = 7.6 Hz, 4H), 1.90–1.81 (m, 2H), 1.22 (q, *J* = 8.0 Hz, 6H). ^{13}C NMR (100 MHz, CD_3OD) δ 171.2, 165.2, 112.8 (d, *J* = 17.2 Hz), 28.4 (d, *J* = 136.6 Hz), 19.6, 19.2, 16.1 (d, *J* = 4.0 Hz), 12.2, 12.2. ^{31}P NMR (162 MHz, CD_3OD) δ 28.19. HRMS m/z calcd for $C_9H_{16}NO_4P$ [M - H]$^-$ 232.0744, found 232.0745.

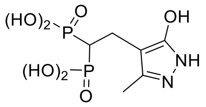

(2-(5-hydroxy-3-methyl-1H-pyrazol-4-yl)ethane-1,1-diyl)bis(phosphonic acid) (**12a**)

Yield: 82%, white solid, m.p. >250 ℃. ^{1}H NMR (400 MHz, D_2O) δ 2.78–2.69 (m, 2H), 2.20–2.04 (m, 1H), 2.12 (s, 3H). ^{13}C NMR (100 MHz, D_2O) δ 162.2, 144.8, 101.9, 39.4 (t, *J* = 116.2 Hz), 18.1, 9.9. ^{31}P NMR (162 MHz, D_2O) δ 19.67. HRMS m/z calcd for $C_6H_{12}N_2O_7P_2$ [M - H]$^-$ 285.0047, found 285.0044.

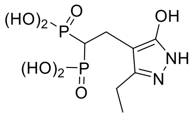

(2-(3-ethyl-5-hydroxy-1H-pyrazol-4-yl)ethane-1,1-diyl)bis(phosphonic acid) (**12b**)

Yield: 80%, white solid, m.p. >250 ℃. ^{1}H NMR (400 MHz, D_2O) δ 2.86–2.76 (m, 2H), 2.61 (q, *J* = 7.6 Hz, 2H), 2.20–2.07 (m, 1H), 1.12 (t, *J* = 7.6 Hz, 3H). ^{13}C NMR (100 MHz, D_2O) δ 158.3, 150.0, 100.9, 39.5 (t, *J* = 118.2 Hz), 17.9, 15.8, 11.4. ^{31}P NMR (162 MHz, D_2O) δ 19.67. HRMS m/z calcd for $C_7H_{14}N_2O_7P_2$ [M - H]$^-$ 299.0203, found 299.0201.

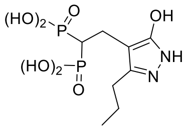

(2-(5-hydroxy-3-propyl-1H-pyrazol-4-yl)ethane-1,1-diyl)bis(phosphonic acid) (**12c**)

Yield: 78%, white solid, m.p. >250 ℃. ^{1}H NMR (400 MHz, D_2O) δ 2.88–2.78 (m, 2H), 2.58 (t, *J* = 7.6 Hz, 2H), 2.20–2.06 (m, 1H), 1.55 (q, *J* = 7.6 Hz, 2H), 0.82 (t, *J* = 7.2 Hz, 3H). ^{13}C NMR (100 MHz, D_2O) δ 157.1, 148.7, 101.5, 39.5 (t, *J* = 118.6 Hz), 26.0, 20.9, 18.0, 12.7. ^{31}P NMR (162 MHz, D_2O) δ 19.72. HRMS m/z calcd for $C_8H_{16}N_2O_7P_2$ [M - H]$^-$ 313.0360, found 313.0362.

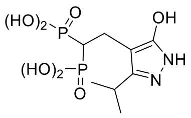

(2-(5-hydroxy-3-isopropyl-1H-pyrazol-4-yl)ethane-1,1-diyl)bis(phosphonic acid) (**12d**)

Yield: 78%, white solid, m.p. >250 ℃. ^{1}H NMR (400 MHz, D_2O) δ 3.14–3.10 (m, 1H), 2.87–2.78 (m, 2H), 2.18–2.10 (m, 1H), 1.14 (d, *J* = 6.6 Hz, 6H). ^{13}C NMR (100 MHz, D_2O) δ 157.5, 153.9, 100.5, 39.8 (t, *J* = 118.6 Hz), 24.9, 18.0, 15.8. ^{31}P NMR (162 MHz, D_2O) δ 19.73. HRMS m/z calcd for $C_8H_{16}N_2O_7P_2$ [M - H]$^-$ 313.0360, found 313.0361.

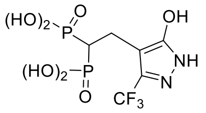

(2-(5-hydroxy-3-(trifluoromethyl)-1H-pyrazol-4-yl)ethane-1,1-diyl)bis(phosphonic acid) (**12e**)

Yield: 80%, white solid, m.p. 225−226 ℃. ^{1}H NMR (400 MHz, D_2O) δ 2.98–2.89 (m, 2H), 2.20–2.07 (m, 1H). ^{13}C NMR (100 MHz, D_2O) δ 155.1, 138.5 (q, *J* = 36.6 Hz), 121.3 (q, *J* = 268.6 Hz), 99.5, 40.2 (t, *J* = 116.2 Hz), 18.2. ^{31}P NMR (162 MHz, D_2O) δ 19.98. ^{19}F NMR (376 MHz, D_2O) δ -61.74. HRMS m/z calcd for $C_6H_9F_3N_2O_7P_2$ [M - H]$^-$ 339.9837, found 339.9840.

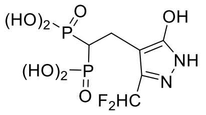

(2-(3-(difluoromethyl)-5-hydroxy-1H-pyrazol-4-yl)ethane-1,1-diyl)bis(phosphonic acid) (**12f**)

Yield: 81%, white solid, m.p. 228−229 ℃. ^{1}H NMR (400 MHz, D_2O) δ 6.81 (q, *J* = 52.6 Hz, 1H), 2.91–2.81 (m, 2H), 2.49–2.32 (m, 1H). ^{13}C NMR (100 MHz, D_2O) 156.3, 138.5 (t, *J* = 26.4 Hz), 108.4 (t, *J* = 237.2 Hz), 102.0, 37.6 (t, *J* = 125.6 Hz), 17.2. ^{31}P NMR (162 MHz, D_2O) δ 20.33. ^{19}F NMR (376 MHz, D_2O) δ -117.89. HRMS m/z calcd for $C_6H_{10}F_2N_2O_7P_2$ [M - H]$^-$ 320.9859, found 320.9861.

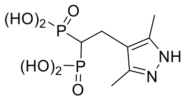

(2-(3,5-dimethyl-1H-pyrazol-4-yl)ethane-1,1-diyl)bis(phosphonic acid) (**12g**)

Yield: 88%, white solid, m.p. >250 ℃. ^{1}H NMR (400 MHz, D_2O) δ 2.83–2.76 (m, 2H), 2.17 (s, 6H), 1.98–1.91 (m, 1H). ^{13}C NMR (100 MHz, D_2O) δ 144.25, 116.23, 40.56 (t, *J* = 114.6 Hz), 20.11, 10.48. ^{31}P NMR (162 MHz, D_2O) δ 19.75. HRMS m/z calcd for $C_7H_{14}N_2O_6P_2$ [M - H]$^-$ 283.0254, found 283.0255.

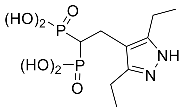

(2-(3,5-diethyl-1H-pyrazol-4-yl)ethane-1,1-diyl)bis(phosphonic acid) (**12h**)

Yield: 85%, white solid, m.p. >250 ℃. ^{1}H NMR (400 MHz, D_2O) δ 2.90–2.81 (m, 2H), 2.70 (q, *J* = 7.6 Hz, 4H), 2.00–1.86 (m, 1H), 1.15 (t, *J* = 7.6 Hz, 6H). ^{13}C NMR (100 MHz, D_2O) δ 149.9, 115.9, 41.7 (t, J = 117.6 Hz), 21.2, 18.6, 12.8. ^{31}P NMR (162 MHz, D_2O) δ 20.48. HRMS m/z calcd for $C_9H_{18}N_2O_6P_2$ [M - H]$^-$ 311.0567, found 311.0566.

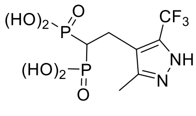

(2-(3-methyl-5-(trifluoromethyl)-1H-pyrazol-4-yl)ethane-1,1-diyl)bis(phosphonic acid) (**12i**)

Yield: 83%, white solid, m.p. >250 ℃. ^{1}H NMR (400 MHz, D_2O) δ 2.99–2.90 (m, 2H), 2.24 (s, 3H), 2.17–2.08 (m, 1H). ^{13}C NMR (100 MHz, D_2O) δ 141.0, 139.5 (q, *J* = 35.8 Hz), 121.9 (q, *J* = 269.0 Hz), 115.4, 40.0 (t, *J* = 114.6 Hz), 18.5, 9.0. ^{31}P NMR (162 MHz, D_2O) δ 18.71. ^{19}F NMR (376 MHz, D_2O) δ -60.48. HRMS m/z calcd for $C_7H_{11}F_3N_2O_6P_2$ [M - H]$^-$ 336.9972, found 336.9975.

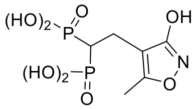

(2-(3-hydroxy-5-methylisoxazol-4-yl)ethane-1,1-diyl)bis(phosphonic acid) (**13a**)

Yield: 79%, colorless oil. ^{1}H NMR (400 MHz, D_2O) δ 2.67–2.59 (m, 2H), 2.54–2.45 (m, 1H), 2.05 (s, 3H). ^{13}C NMR (100 MHz, D_2O) δ 174.7, 163.3, 95.2, 35.7 (t, *J* = 126.2 Hz), 17.7, 9.9. ^{31}P NMR (162 MHz, D_2O) δ 20.72. HRMS m/z calcd for $C_6H_{11}NO_8P_2$ $[M - H]^-$ 285.9887, found 285.9885.

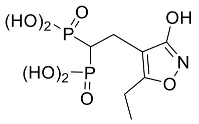

(2-(5-ethyl-3-hydroxyisoxazol-4-yl)ethane-1,1-diyl)bis(phosphonic acid) (**13b**)

Yield: 76%, colorless oil. ^{1}H NMR (400 MHz, D_2O) δ 2.67–2.59 (m, 3H), 2.49 (q, *J* = 7.6 Hz, 2H), 0.99 (t, *J* = 7.6 Hz, 3H). ^{13}C NMR (100 MHz, D_2O) δ 174.6, 167.3, 93.9, 35.5 (t, *J* = 127.0 Hz), 18.3, 15.5, 10.2. ^{31}P NMR (162 MHz, D_2O) δ 20.74. HRMS m/z calcd for $C_7H_{13}NO_8P_2$ $[M - H]^-$ 300.0044, found 300.0044.

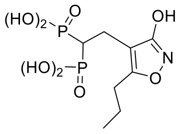

(2-(3-hydroxy-5-propylisoxazol-4-yl)ethane-1,1-diyl)bis(phosphonic acid) (**13c**)

Yield: 75%, colorless oil. ^{1}H NMR (400 MHz, D_2O) δ 2.62–2.54 (m, 3H), 2.37 (t, *J* = 7.6 Hz, 2H), 1.38 (q, *J* = 7.6 Hz, 2H), 0.69 (t, *J* = 7.4 Hz, 3H). ^{13}C NMR (100 MHz, D_2O) δ 174.5, 165.9, 94.2, 35.5 (t, *J* = 126.6 Hz), 26.4, 19.8, 12.7. ^{31}P NMR (162 MHz, D_2O) δ 23.22. HRMS m/z calcd for $C_8H_{15}NO_8P_2$ $[M - H]^-$ 314.0200, found 314.0200.

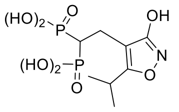

(2-(3-hydroxy-5-isopropylisoxazol-4-yl)ethane-1,1-diyl)bis(phosphonic acid) (**13d**)

Yield: 77%, colorless oil. ^{1}H NMR (400 MHz, D_2O) δ 2.93 (t, *J* = 7.2 Hz, 1H), 2.66–2.39 (m, 3H), 0.95 (d, *J* = 7.2 Hz, 6H). ^{13}C NMR (100 MHz, D_2O) δ 174.5, 170.6, 92.8, 35.5 (t, *J* = 127.2 Hz), 25.5, 19.3, 17.5. ^{31}P NMR (162 MHz, D_2O) δ 20.72. HRMS m/z calcd for $C_8H_{15}NO_8P_2$ $[M - H]^-$ 314.0200, found 314.0202.

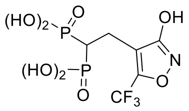

(2-(3-hydroxy-5-(trifluoromethyl)isoxazol-4-yl)ethane-1,1-diyl)bis(phosphonic acid) (**13e**)

Yield: 78%, colorless oil. ^{1}H NMR (400 MHz, D_2O) δ 2.82–2.72 (m, 2H), 2.50–2.35 (m, 1H). ^{13}C NMR (100 MHz, D_2O) δ 176.3, 156.7 (q, *J* = 36.2 Hz), 122.0 (q, *J* = 271.8 Hz), 90.9, 39.7 (t, *J* = 126.0 Hz), 19.5. ^{31}P NMR (162 MHz, D_2O) δ 20.45. ^{19}F NMR (376 MHz, D_2O) δ -64.16. HRMS m/z calcd for $C_6H_8F_3NO_8P_2$ $[M - H]^-$ 339.9604, found 339.9601.

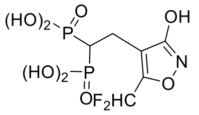

(2-(5-(1,1-difluoroethyl)-3-hydroxyisoxazol-4-yl)ethane-1,1-diyl)bis(phosphonic acid) (**13f**)

Yield: 77%, colorless oil. ^{1}H NMR (400 MHz, D_2O) δ 6.87 (t, *J* = 52.8 Hz, 1H), 2.90–2.82 (m, 2H), 2.60–2.51 (m, 1H). ^{13}C NMR (100 MHz, D_2O) δ 173.3, 156.4 (t, *J* = 30.6 Hz), 108.9 (t, *J* = 238.6 Hz), 93.5, 36.7 (t, *J* = 126.0 Hz), 17.3. ^{31}P NMR (162 MHz, D_2O) δ 20.17. ^{19}F NMR (376 MHz,

D_2O) δ -120.86. HRMS m/z calcd for $C_6H_9F_2NO_8P_2$ [M - H]$^-$ 321.9699, found 321.9695.

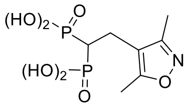

(2-(3,5-dimethylisoxazol-4-yl)ethane-1,1-diyl)bis(phosphonic acid) (**13g**)

Yield: 80%, white solid, m.p. >250 ℃. ^{1}H NMR (400 MHz, D_2O) δ 2.90–2.84 (m, 2H), 2.43–2.33 (m, 1H), 2.27 (d, J = 2.2 Hz, 3H), 2.17 (d, J = 2.2 Hz, 3H). ^{13}C NMR (100 MHz, D_2O) δ 167.0, 160.8, 111.5, 37.0 (t, J = 126.4 Hz), 17.7, 10.2, 9.1. ^{31}P NMR (162 MHz, D_2O) δ 20.43. HRMS m/z calcd for $C_7H_{13}NO_7P_2$ [M - H]$^-$ 284.0094, found 284.0095.

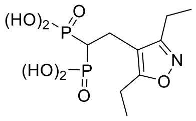

(2-(3,5-diethylisoxazol-4-yl)ethane-1,1-diyl)bis(phosphonic acid) (**13h**)

Yield: 78%, white solid, m.p. >250 ℃. ^{1}H NMR (400 MHz, D_2O) δ 2.81–2.72 (m, 2H), 2.54 (q, J = 7.6 Hz, 2H), 2.47 (q, J = 7.6 Hz, 2H), 2.33–2.21 (m, 1H), 1.04–0.98 (m, 6H). ^{13}C NMR (100 MHz, D_2O) δ 171.4, 165.6, 110.2, 37.5 (t, J = 127.2 Hz), 18.6, 18.1, 17.6, 10.7, 10.7. ^{31}P NMR (162 MHz, D_2O) δ 20.47. HRMS m/z calcd for $C_9H_{17}NO_7P_2$ [M - H]$^-$ 312.0407, found 312.0407.

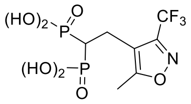

(2-(5-methyl-3-(trifluoromethyl)isoxazol-4-yl)ethane-1,1-diyl)bis(phosphonic acid) (**13i**)

Yield: 77%, white solid, m.p. 240−241 ℃. ^{1}H NMR (400 MHz, D_2O) δ 2.94–2.85 (m, 2H), 2.22 (s, 3H), 2.15–2.04 (m, 1H). ^{13}C NMR (100 MHz, D_2O) δ 171.3, 162.4, 152.7 (q, J = 40.6 Hz), 118.5 (q, J = 260.6 Hz), 39.0 (t, J = 117.8 Hz), 17.8, 9.6. ^{31}P NMR (162 MHz, D_2O) δ 17.85. ^{19}F NMR (376 MHz, D_2O) δ -62.98. HRMS m/z calcd for $C_7H_{10}F_3NO_7P_2$ [M - H]$^-$ 337.9812, found 337.9811.

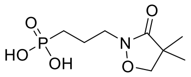

(3-(4,4-dimethyl-3-oxoisoxazolidin-2-yl)propyl)phosphonic acid (**19**)

Yield: 62%, white solid, m.p. >250 ℃. ^{1}H NMR (400 MHz, D_2O) δ 4.18 (s, 2H), 3.61 (t, J = 6.6 Hz, 2H), 1.86–1.80 (m, 2H), 1.49–1.40 (m, 2H), 1.22 (s, 6H). ^{13}C NMR (100 MHz, D_2O) δ 174.3, 79.2, 45.8 (d, J = 19.6 Hz), 43.5, 25.8 (d, J = 132.4 Hz), 21.5 (d, J = 3.2 Hz), 20.6. ^{31}P NMR (162 MHz, D_2O) δ 22.50. HRMS m/z calcd for $C_8H_{16}NO_5P$ [M - H]$^-$ 236.0693, found 236.0690.

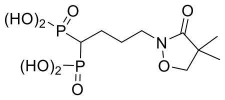

(4-(4,4-dimethyl-3-oxoisoxazolidin-2-yl)butane-1,1-diyl)bis(phosphonic acid) (**20**)

Yield: 55%, white solid, m.p. >250 ℃. ^{1}H NMR (400 MHz, D_2O) δ 4.16 (s, 2H), 3.59 (t, J = 6.2 Hz, 2H), 2.00–1.79 (m, 5H), 1.20 (s, 6H). ^{13}C NMR (100 MHz, D_2O) δ 174.3, 79.2, 44.7, 43.5, 38.5 (t, J = 117.6 Hz), 26.5, 22.9, 20.6. ^{31}P NMR (162 MHz, D_2O) δ 20.00. HRMS m/z calcd for $C_9H_{19}NO_8P_2$ [M - H]$^-$ 330.0513, found 330.0511.

6. Spectra of the target compounds

(2-(5-hydroxy-3-methyl-1H-pyrazol-4-yl)ethyl)phosphonic acid (**5a**)

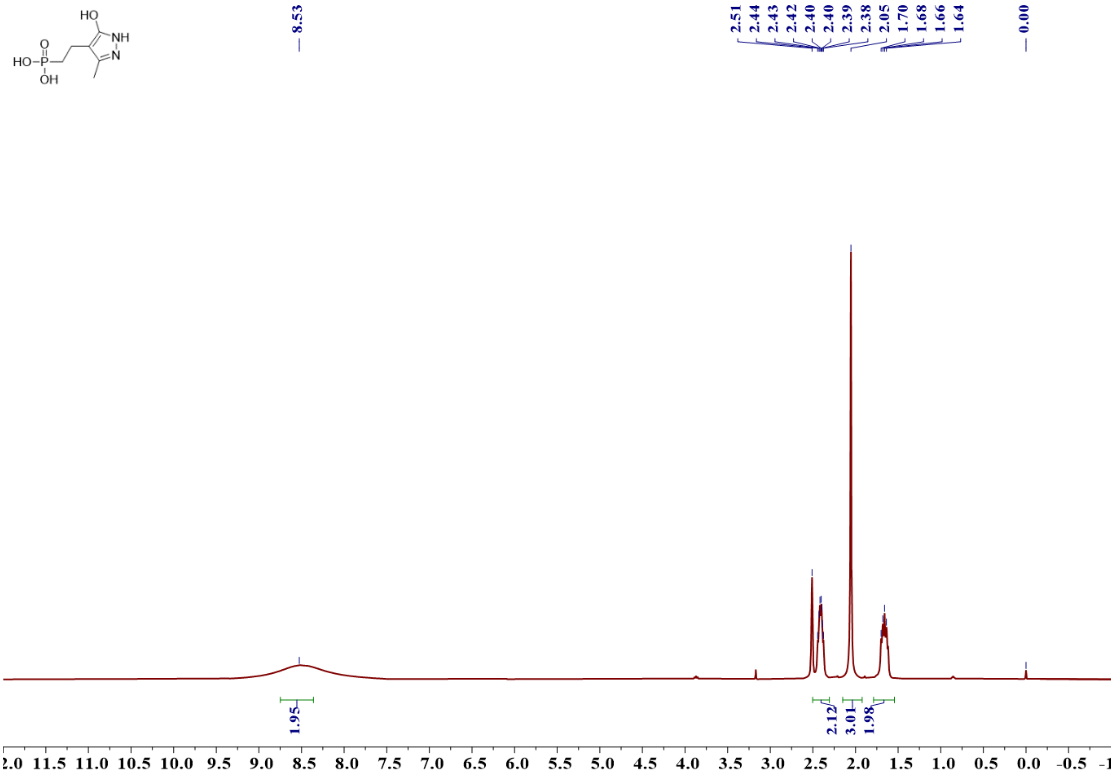

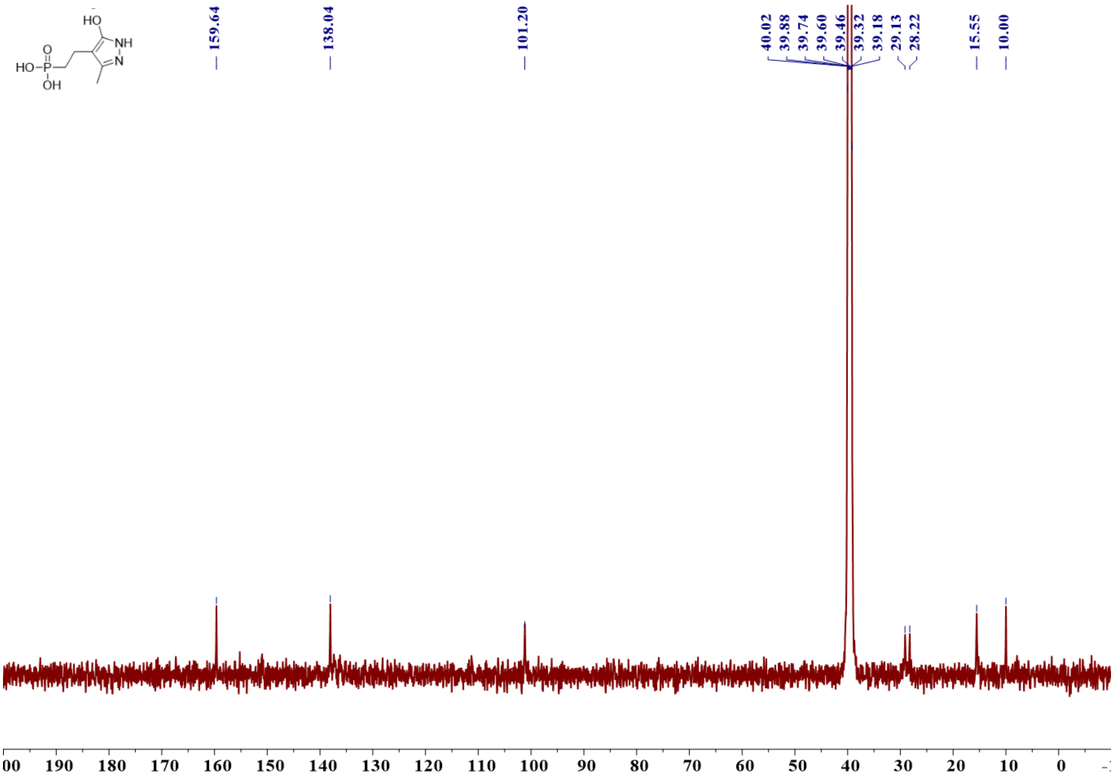

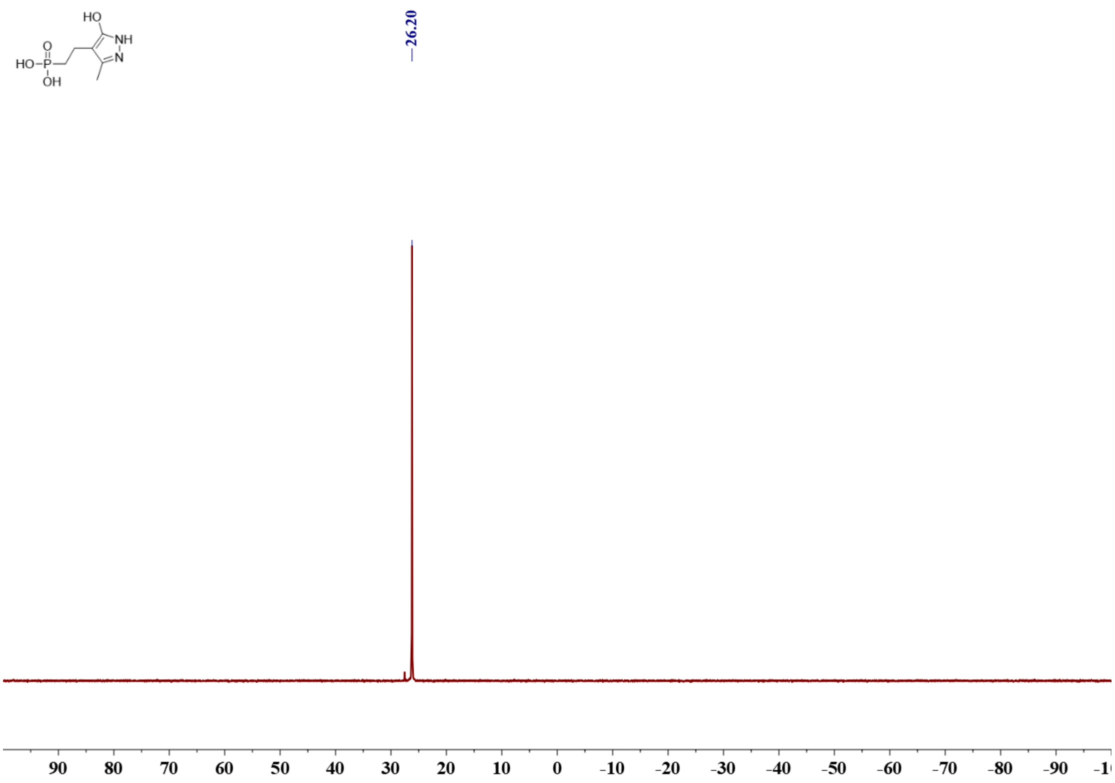
HO
NH
N
O
HO
P
OH
26.20
90
80
70
60
50
40
30
20
10
0
-10
-20
-30
-40
-50
-60
-70
-80
-90

(2-(3-ethyl-5-hydroxy-1H-pyrazol-4-yl)ethyl)phosphonic acid (**5b**)

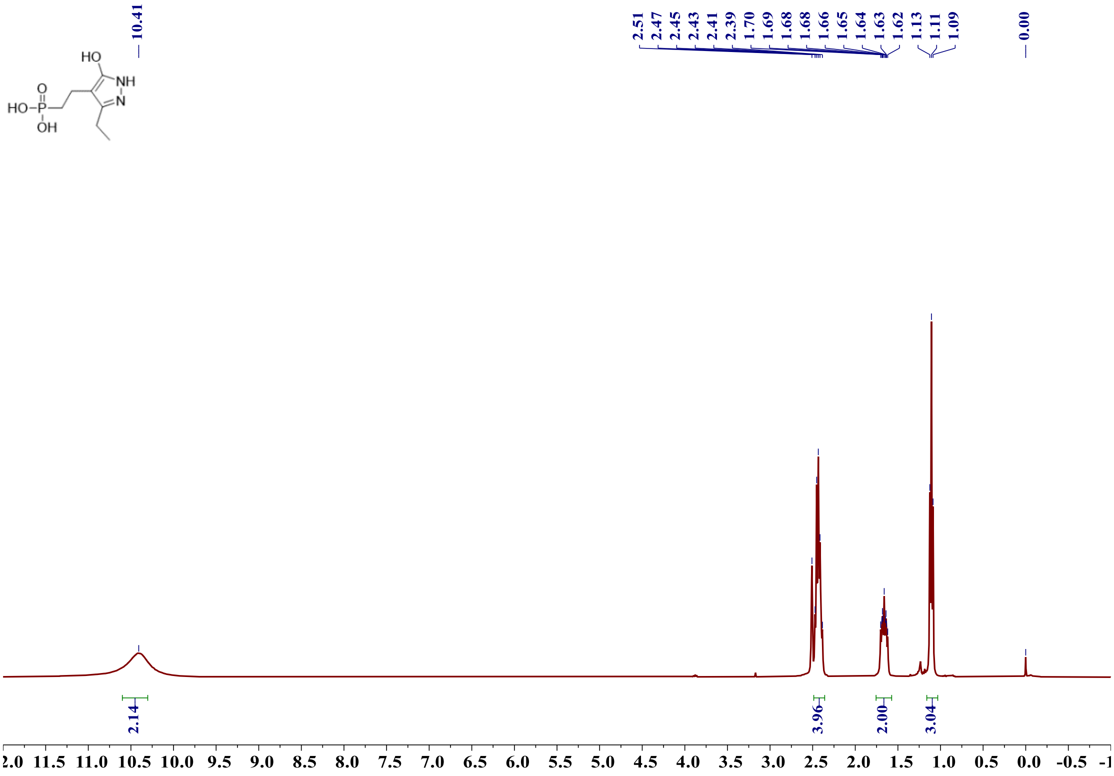

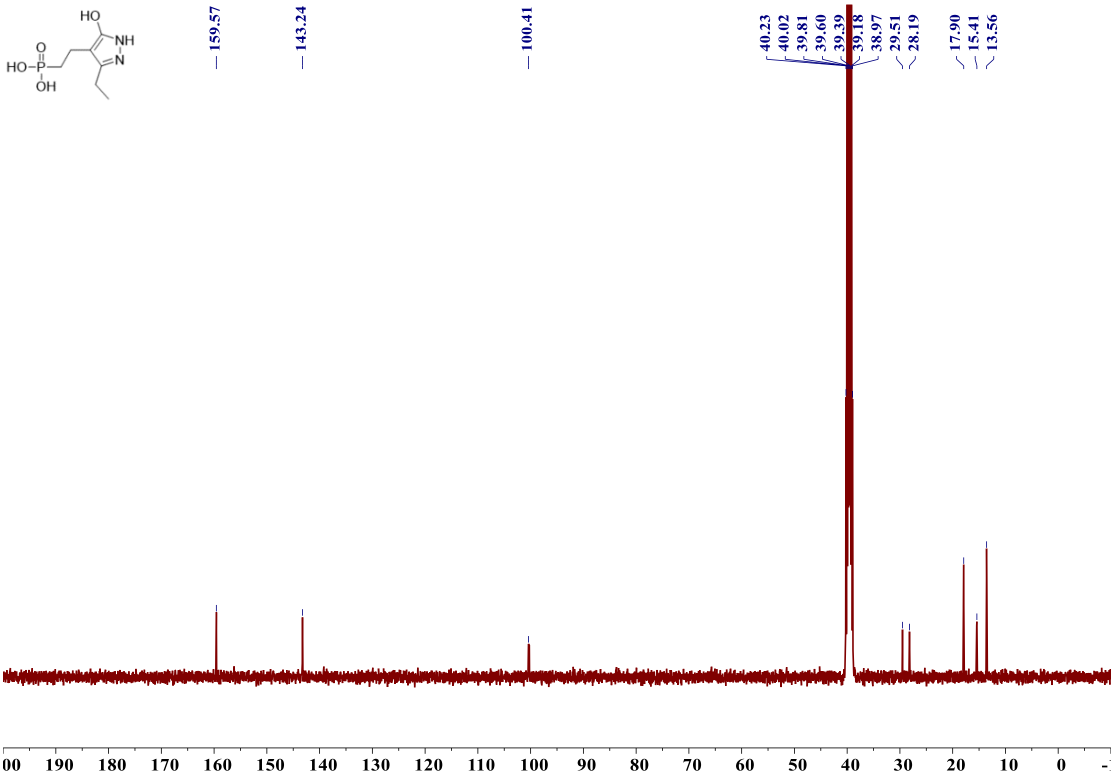

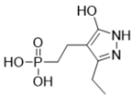

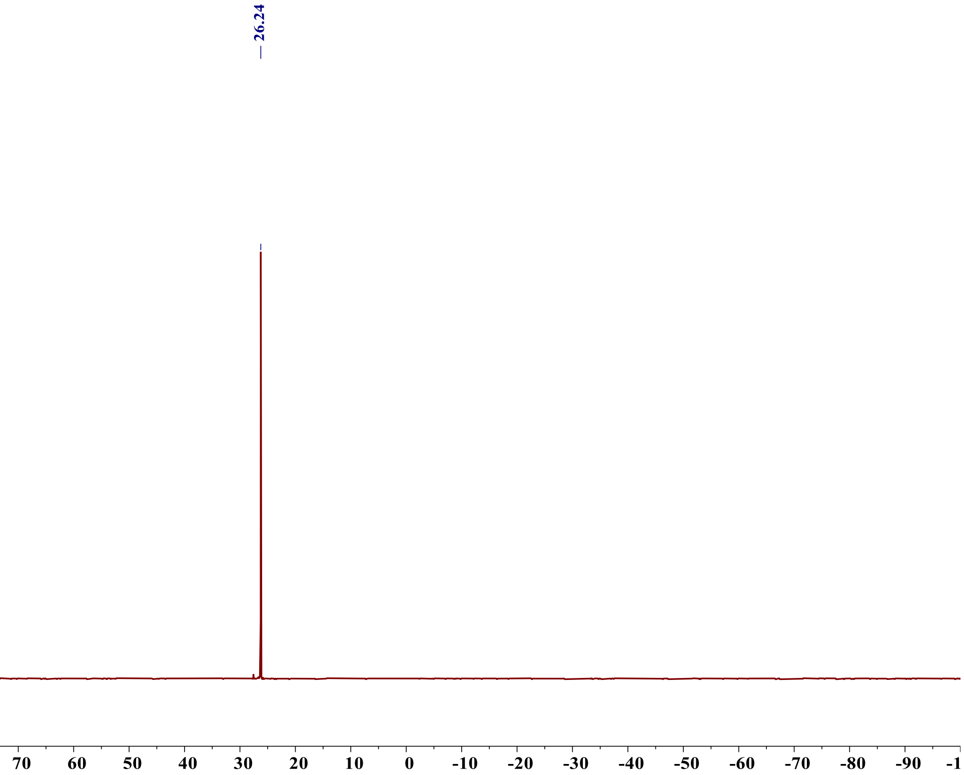
26.24
90
80
70
60
50
40
30
20
10
0
-10
-20
-30
-40
-50
-60
-70
-80
-90
-1

(2-(5-hydroxy-3-propyl-1H-pyrazol-4-yl)ethyl)phosphonic acid (**5c**)

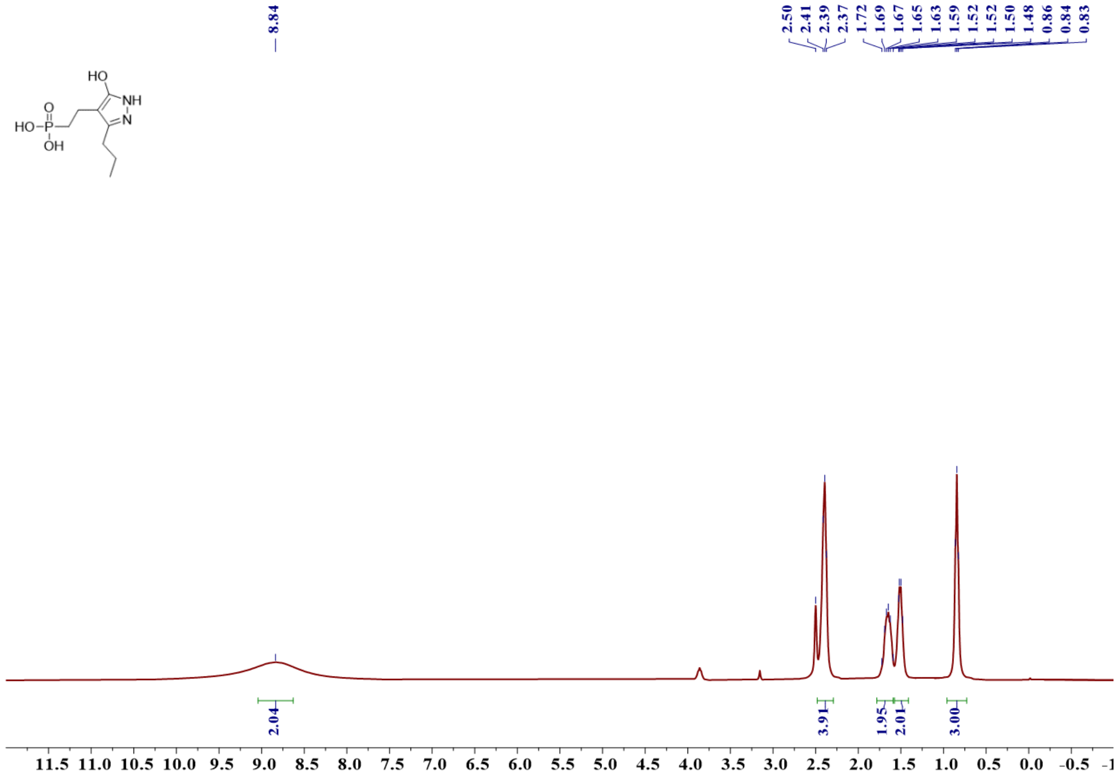

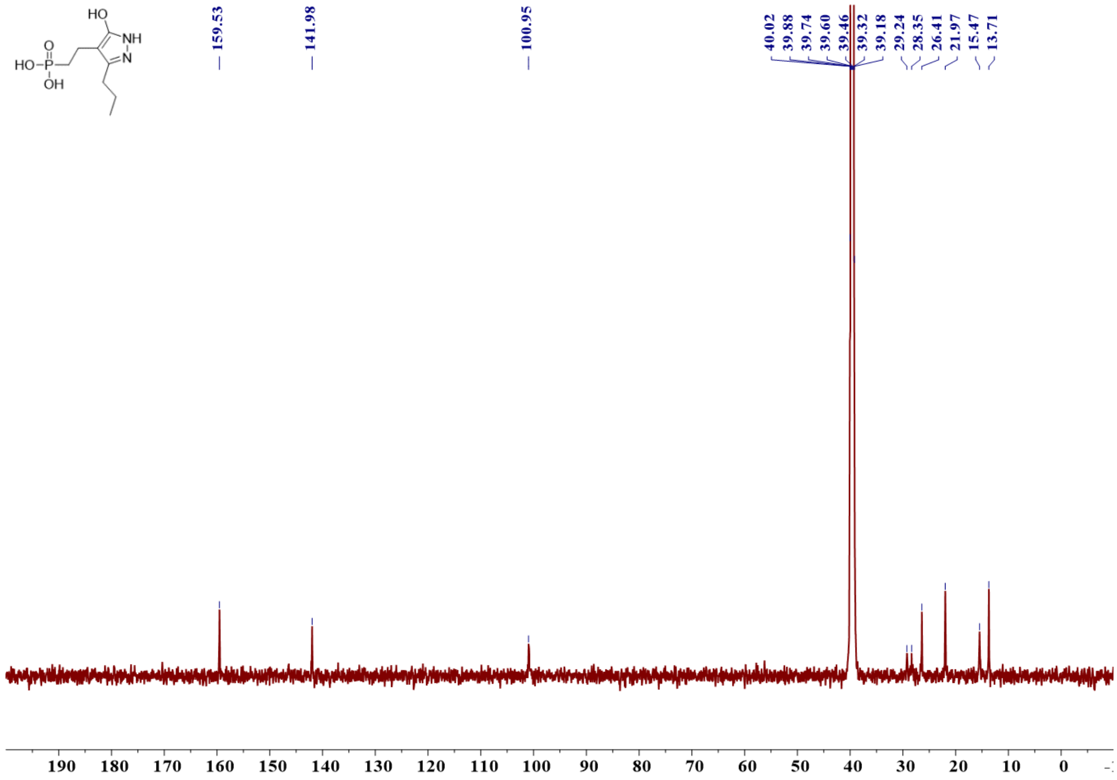

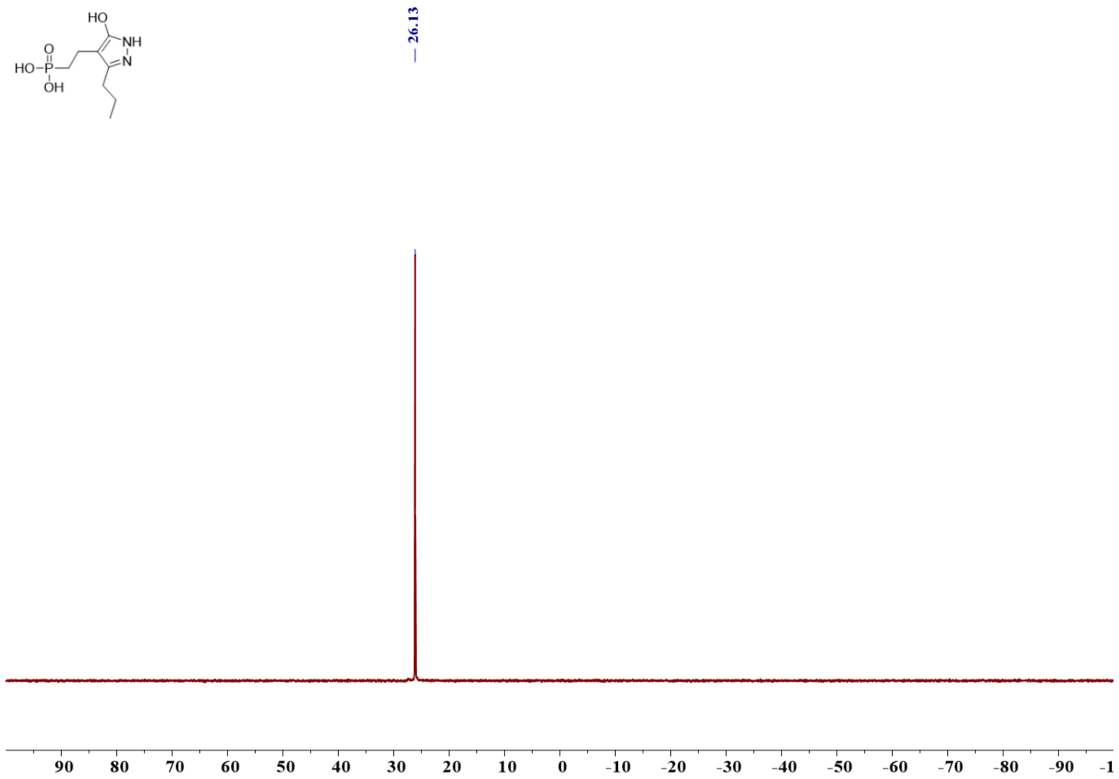
HO
NH
N
O
HO-P
OH
26.13
90
80
70
60
50
40
30
20
10
0
-10
-20
-30
-40
-50
-60
-70
-80
-90
-1

(2-(5-hydroxy-3-isopropyl-1H-pyrazol-4-yl)ethyl)phosphonic acid (**5d**)

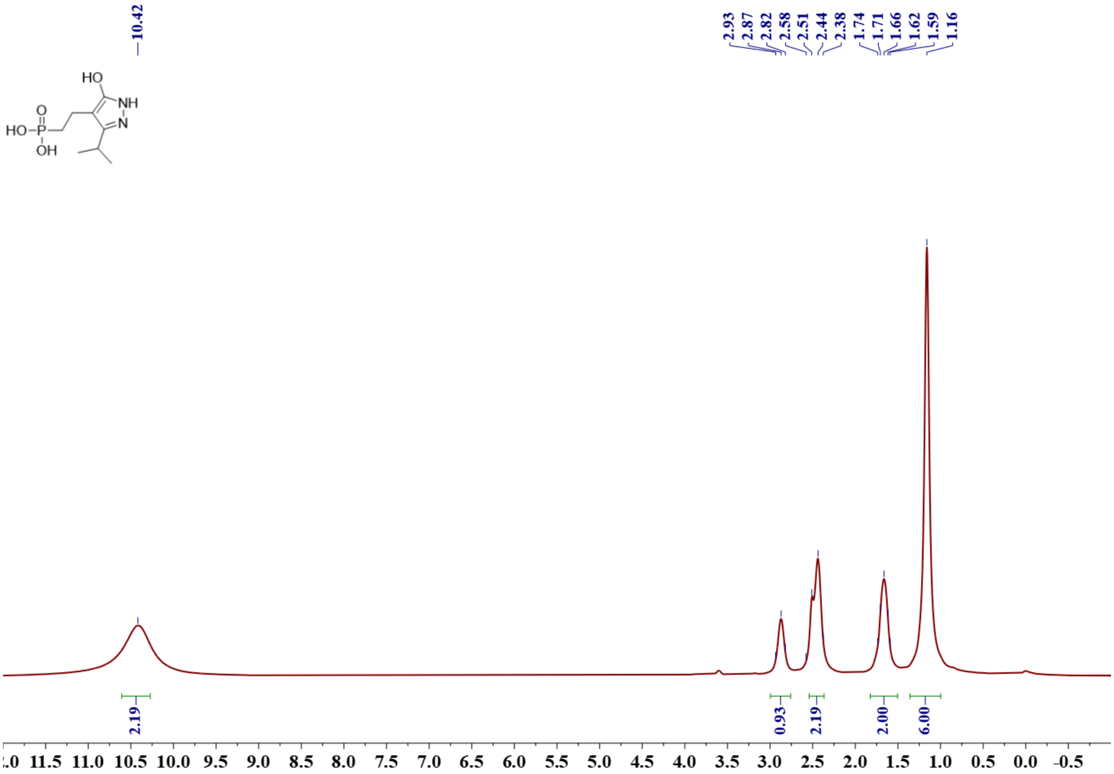

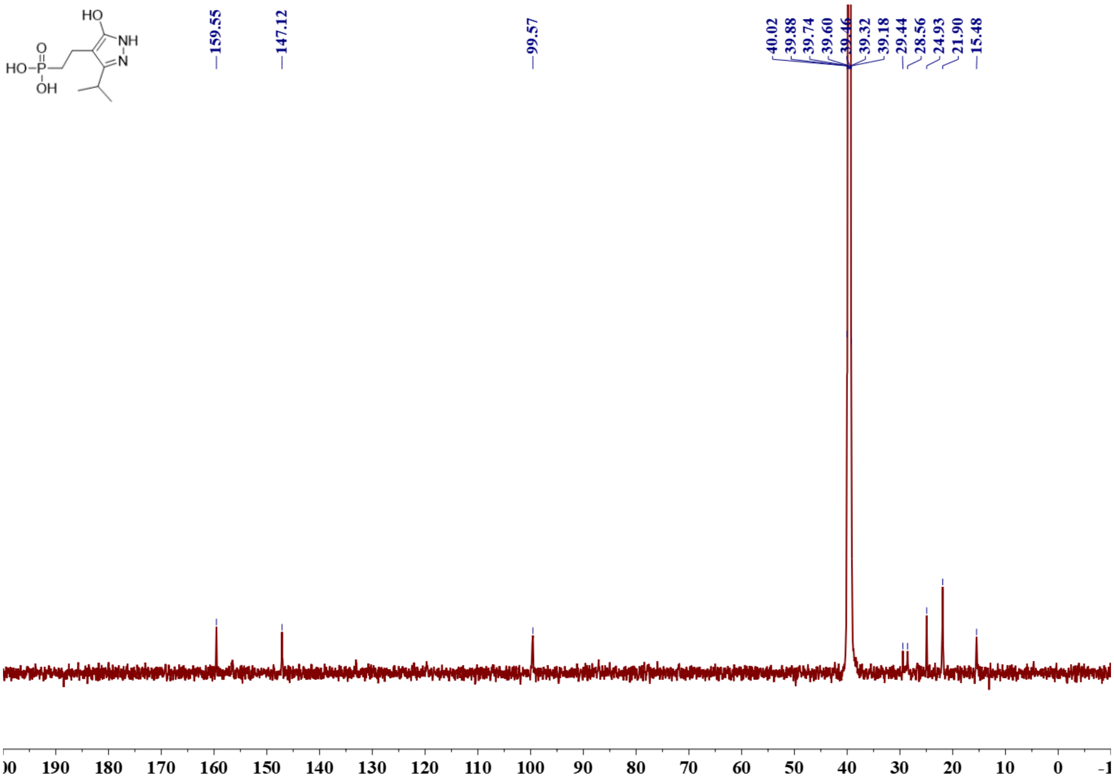

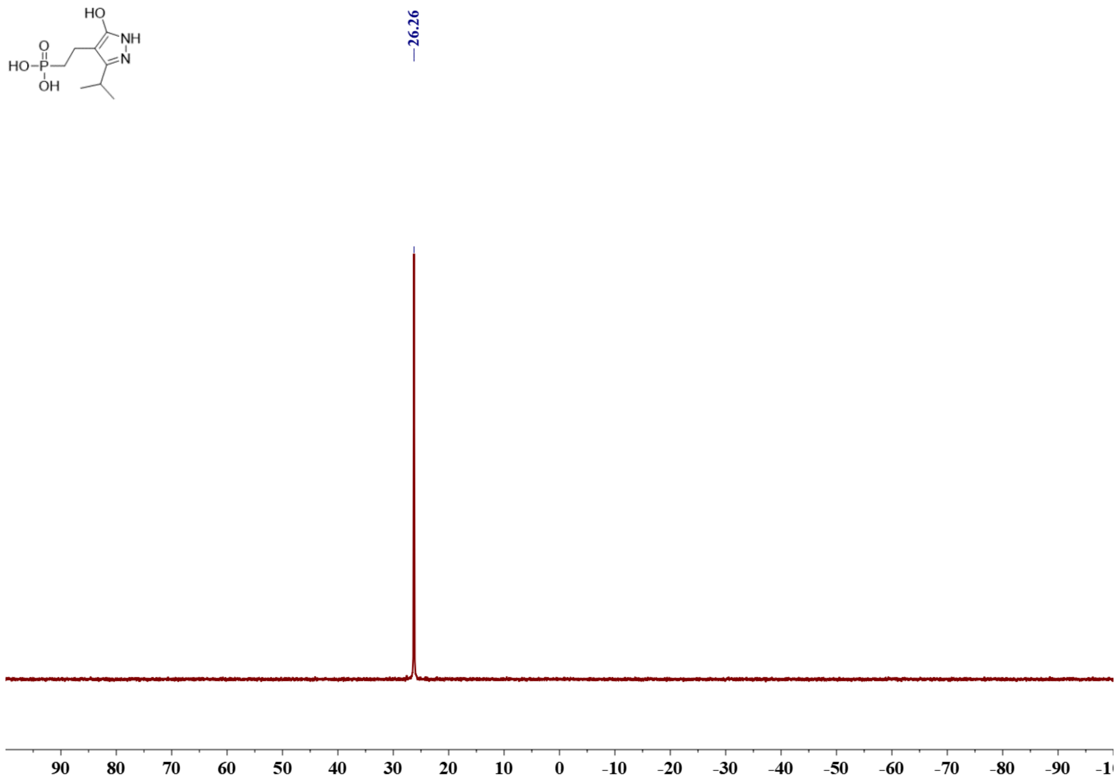
HO
NH
N
O
HO-P
OH
26.26
90
80
70
60
50
40
30
20
10
0
-10
-20
-30
-40
-50
-60
-70
-80
-90

(2-(3,5-dimethyl-4H-pyrazol-4-yl)ethyl)phosphonic acid (**5e**)

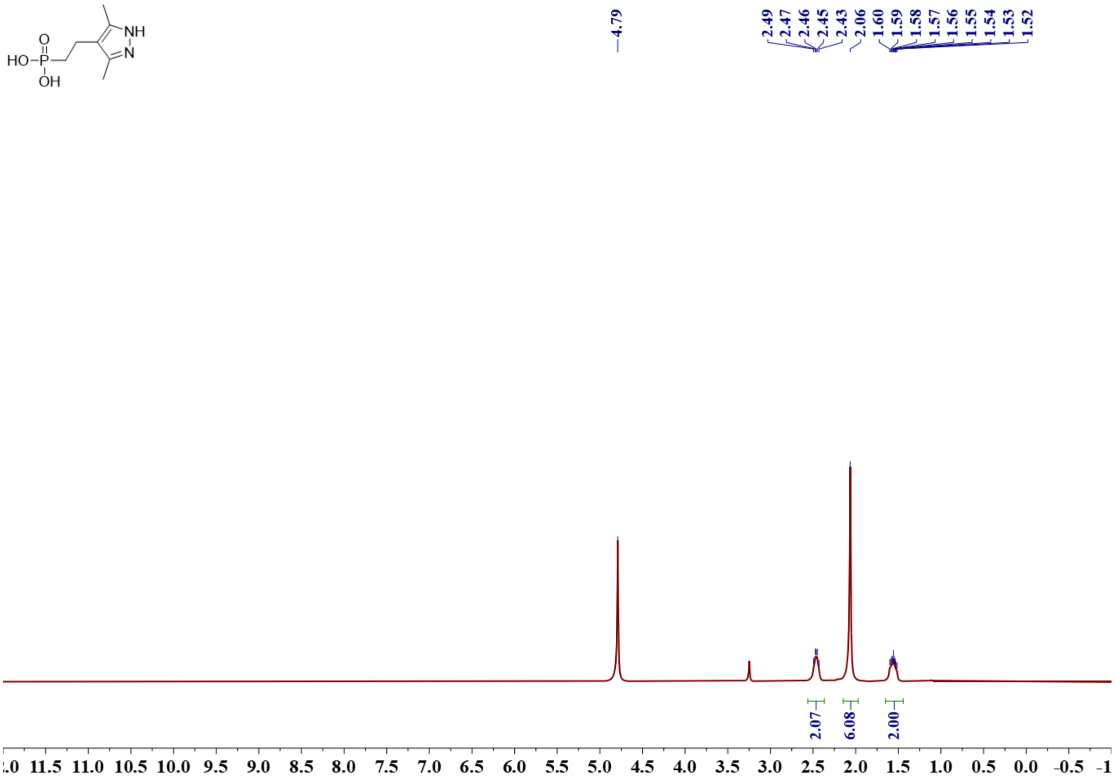

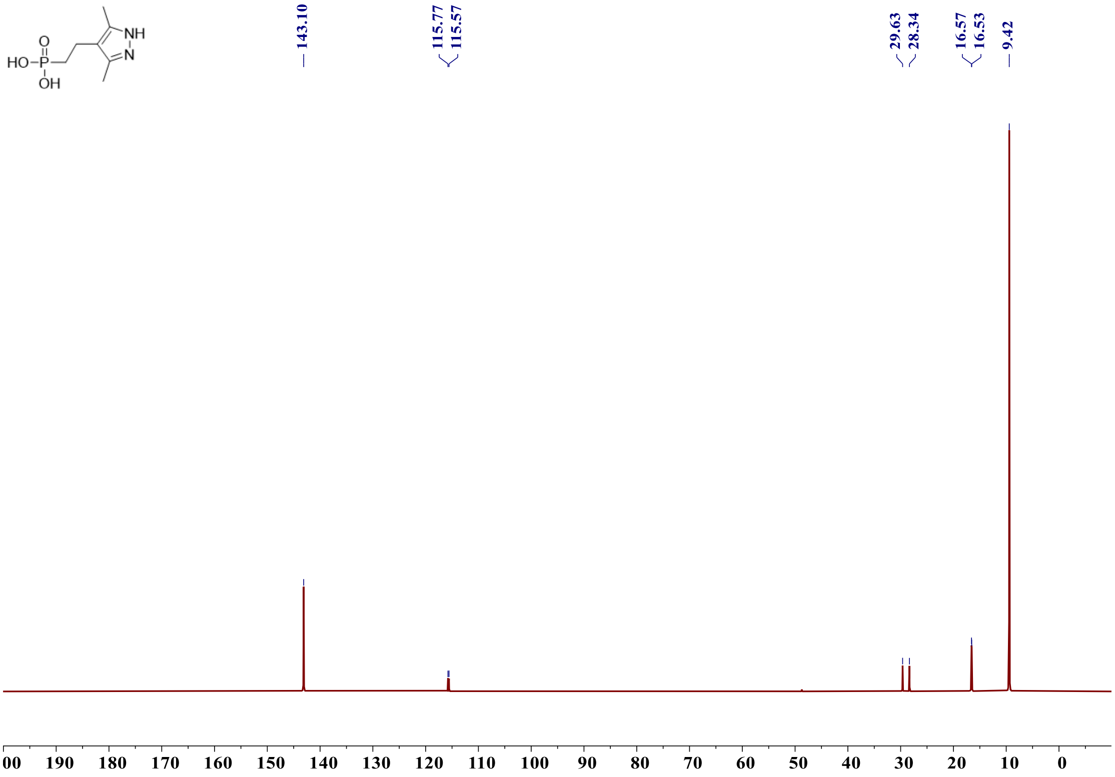

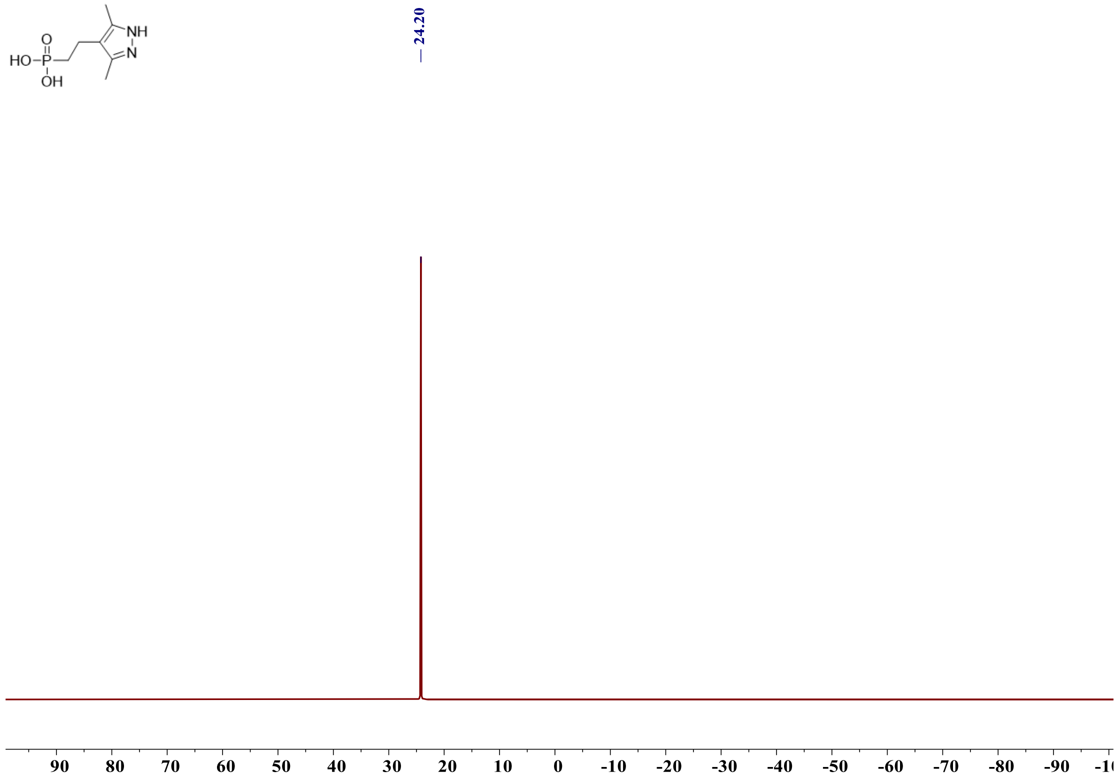

HO
O
P
OH
NH
N
24.20
90
80
70
60
50
40
30
20
10
0
-10
-20
-30
-40
-50
-60
-70
-80
-90

(2-(3,5-diethyl-4H-pyrazol-4-yl)ethyl)phosphonic acid (**5f**)

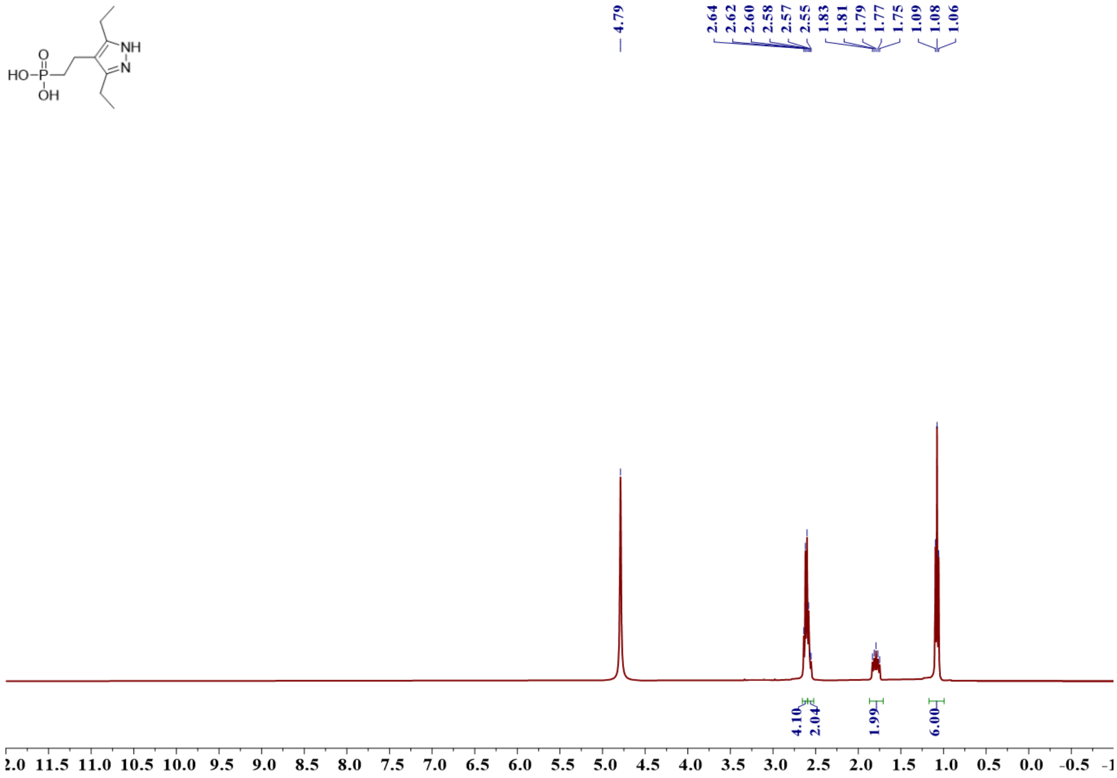

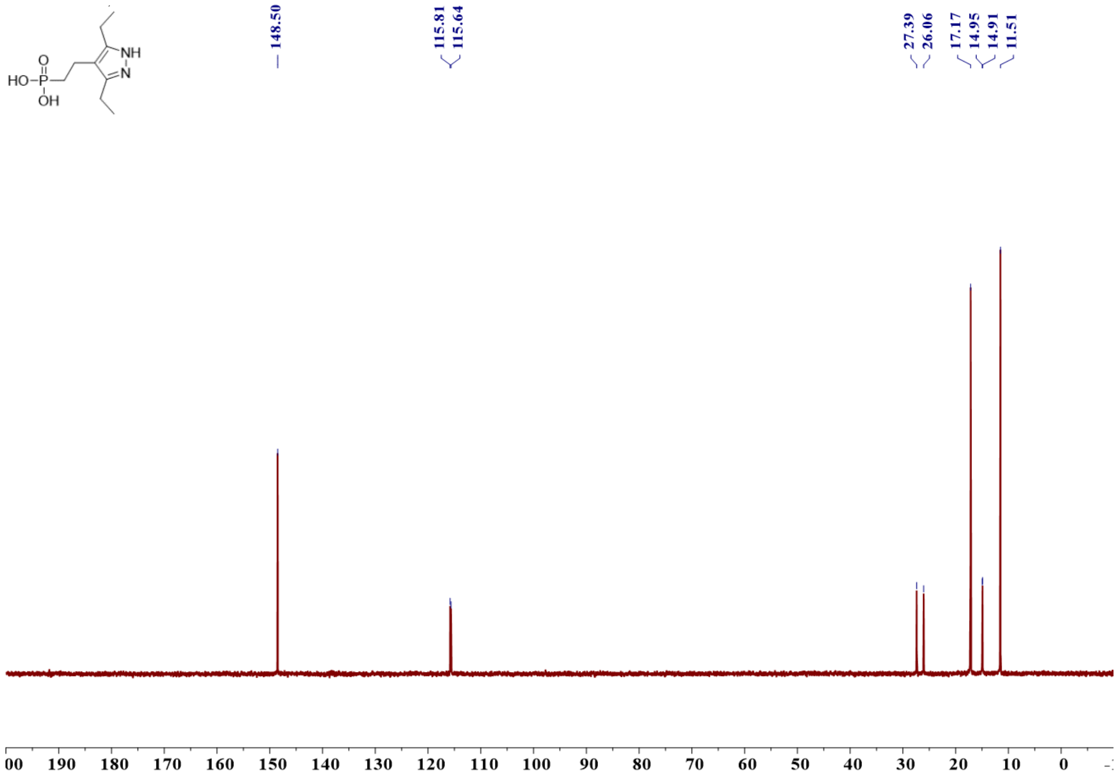

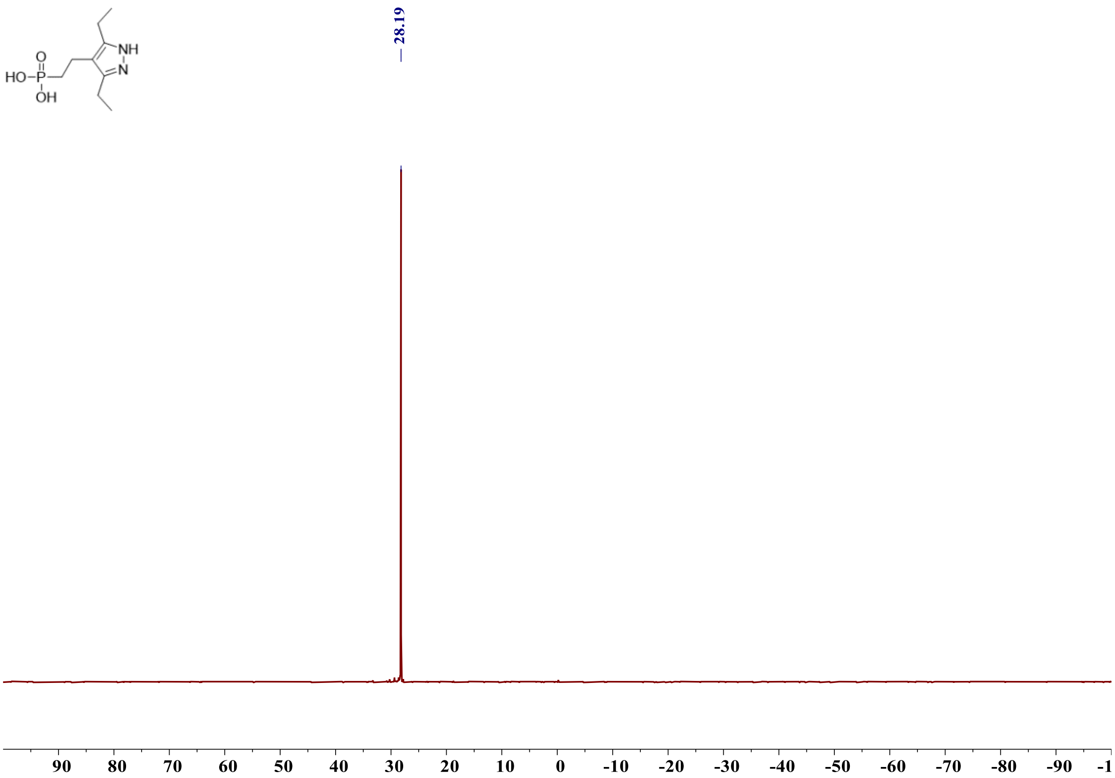
O
HO-P
OH
NH
N
28.19
90 80 70 60 50 40 30 20 10 0 -10 -20 -30 -40 -50 -60 -70 -80 -90 -1

(2-(3-hydroxy-5-methylisoxazol-4-yl)ethyl)phosphonic acid (**6a**)

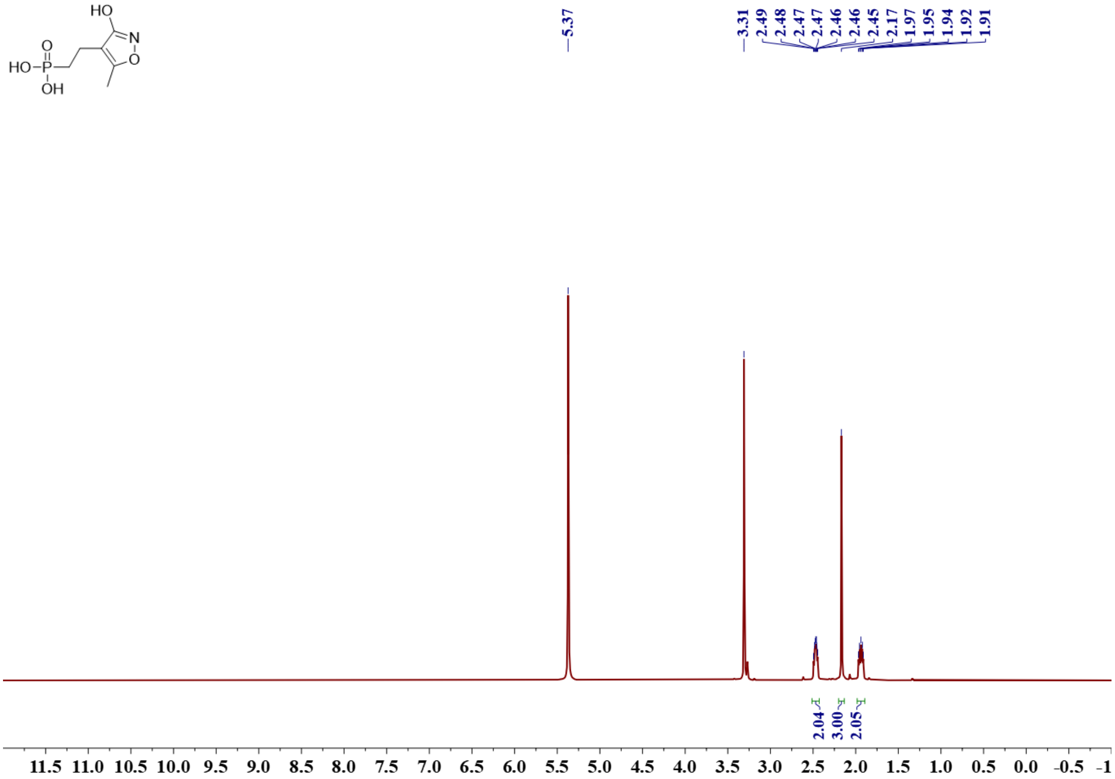

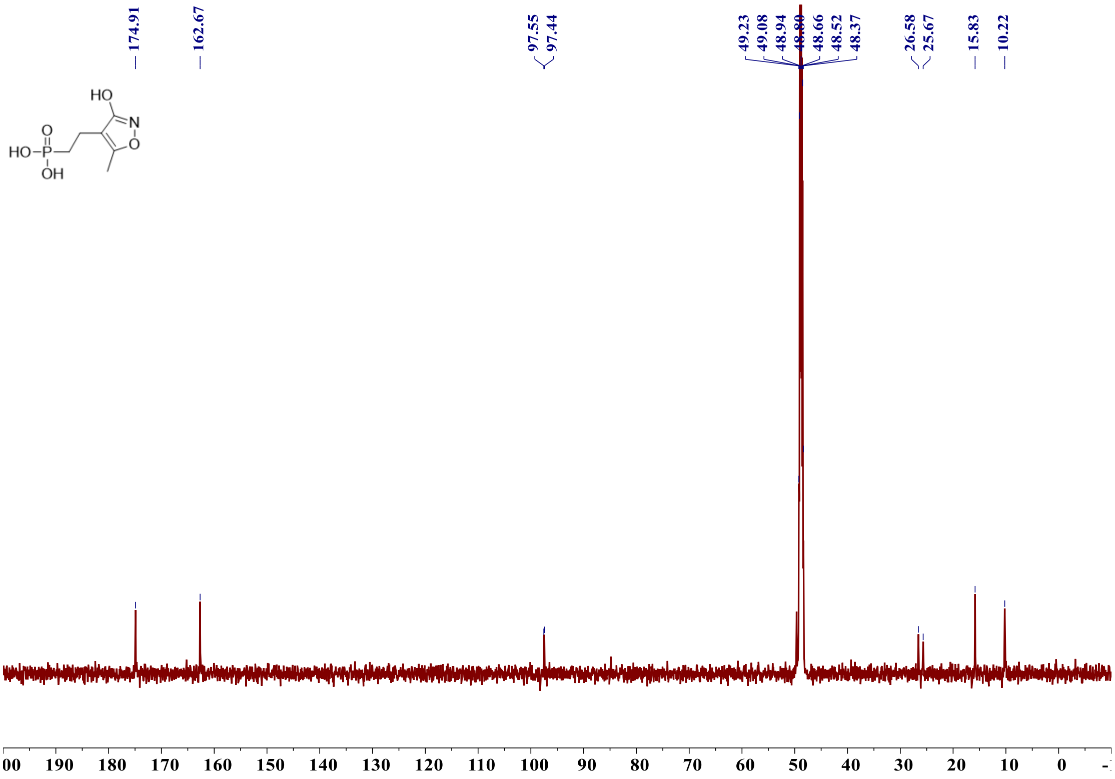

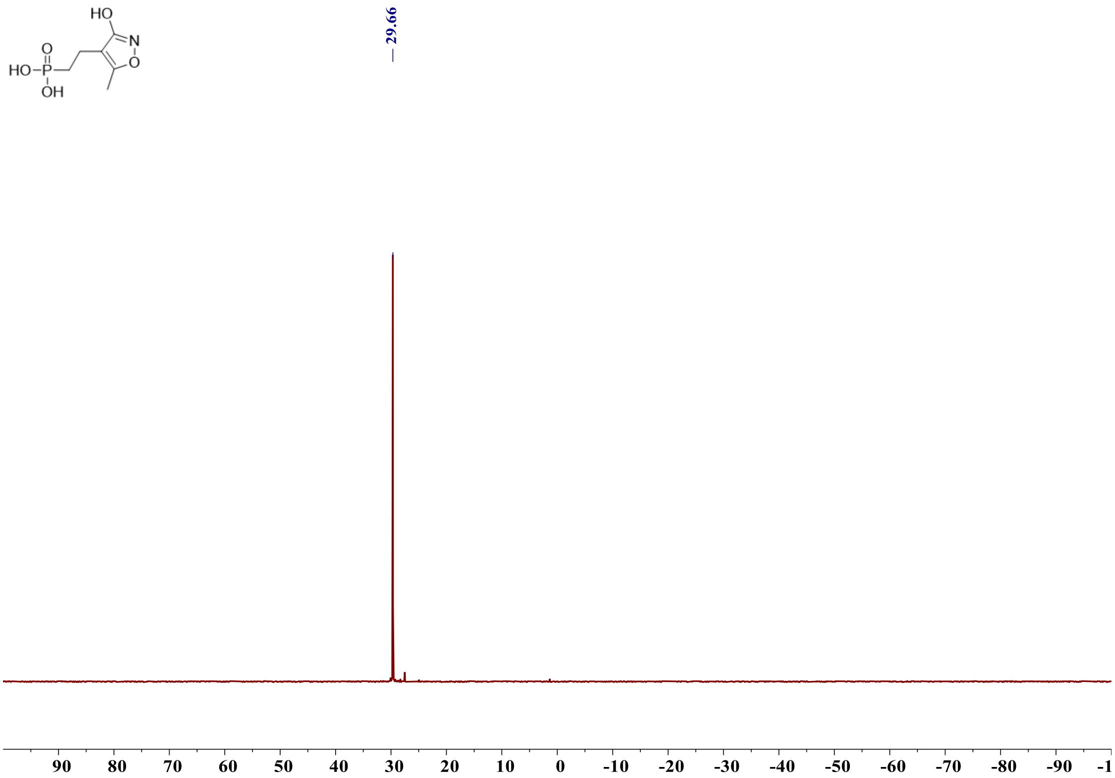
HO
O
HO–P
OH
N
O
— 29.66
90 80 70 60 50 40 30 20 10 0 -10 -20 -30 -40 -50 -60 -70 -80 -90 -1

(2-(5-ethyl-3-hydroxyisoxazol-4-yl)ethyl)phosphonic acid (**6b**)

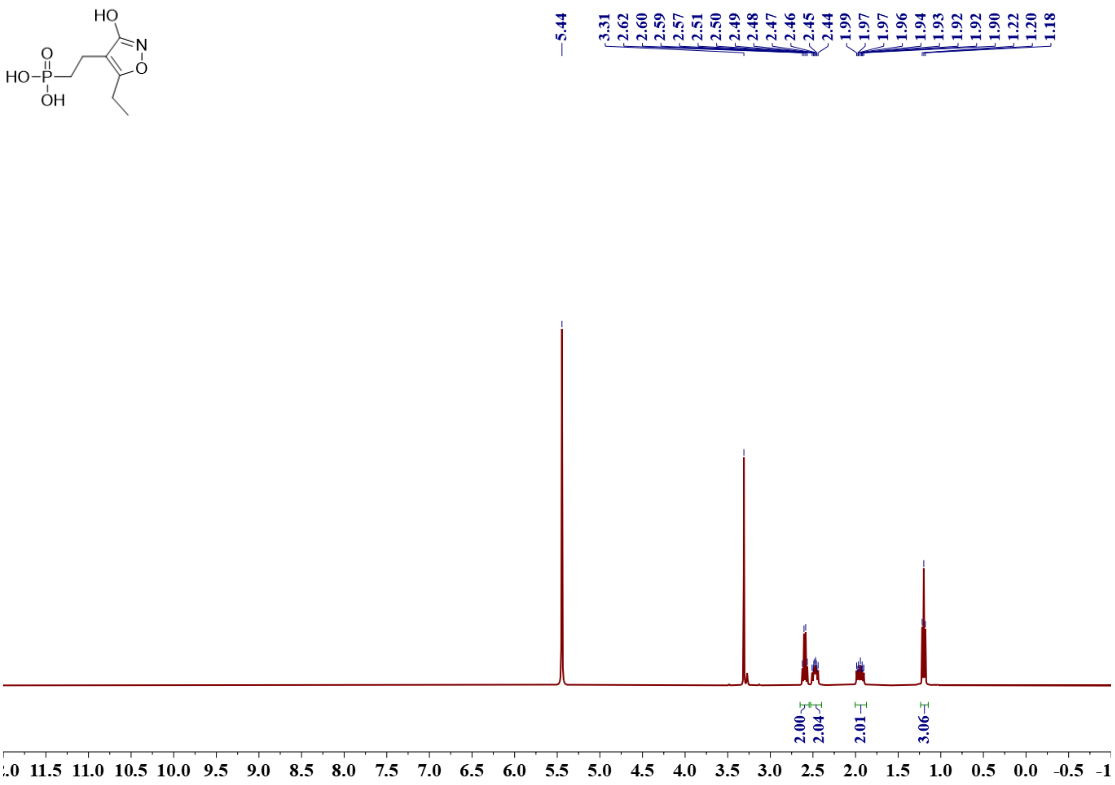

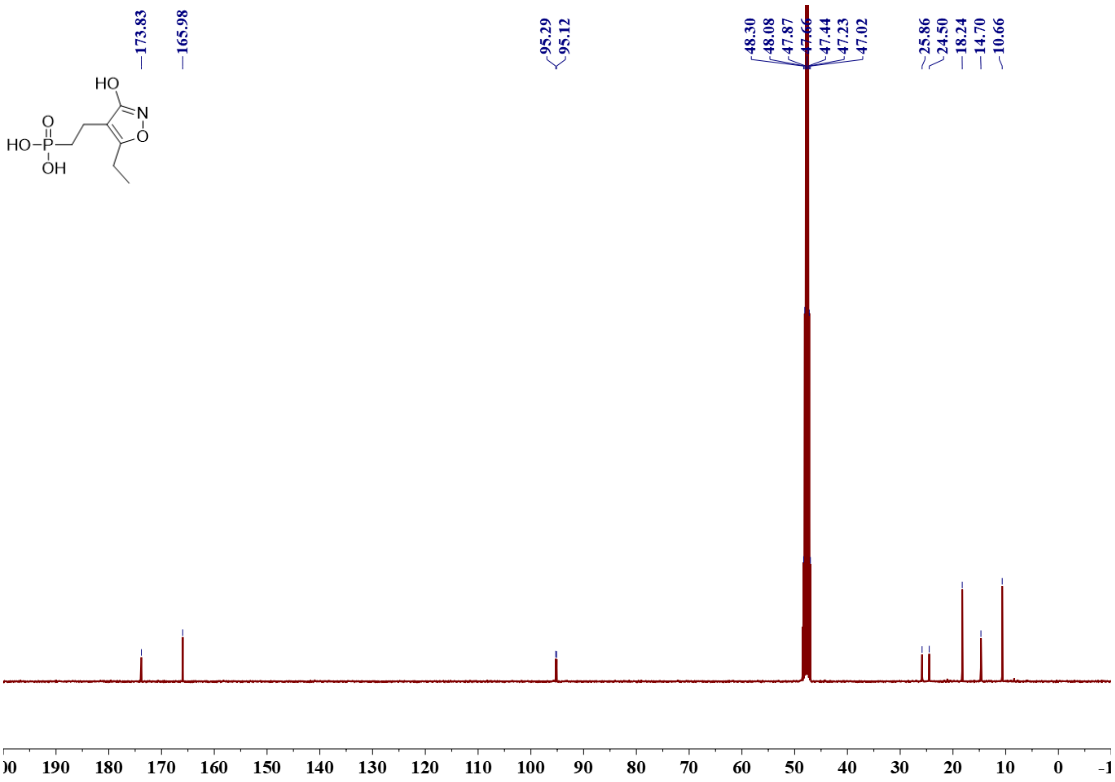

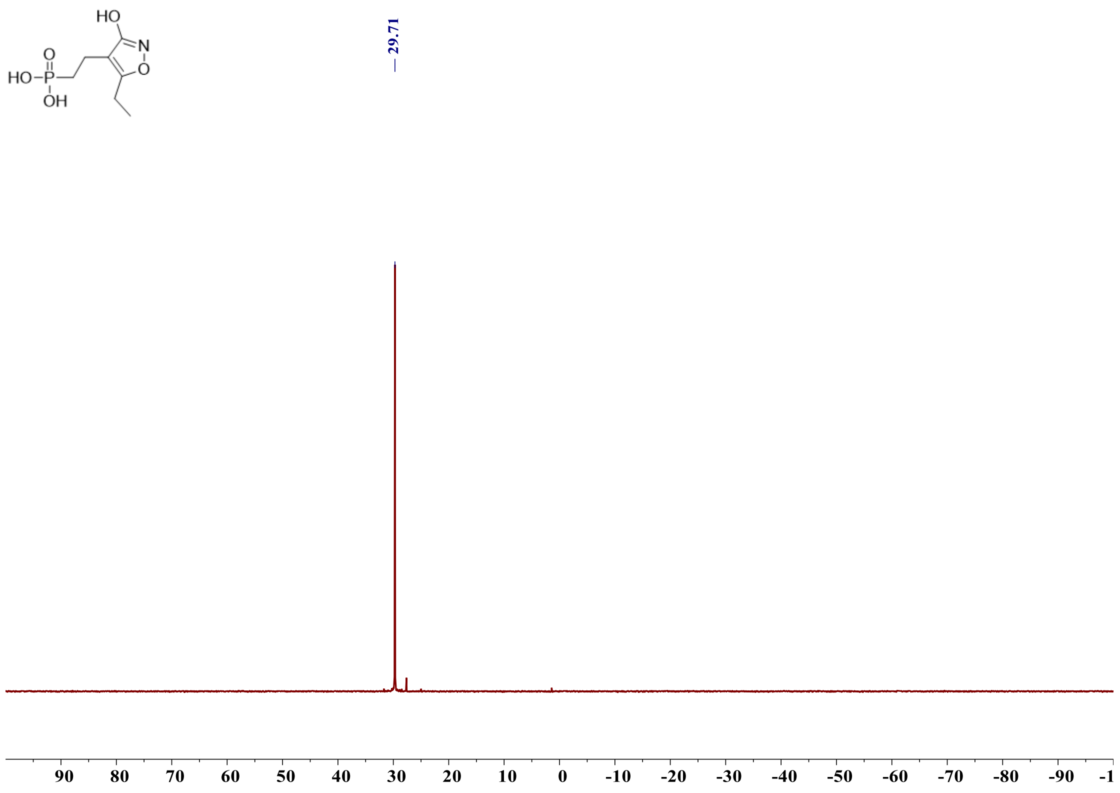

HO
N
O
O
HO
P
OH
29.71
90
80
70
60
50
40
30
20
10
0
-10
-20
-30
-40
-50
-60
-70
-80
-90
-1

(2-(3-hydroxy-5-propylisoxazol-4-yl)ethyl)phosphonic acid (**6c**)

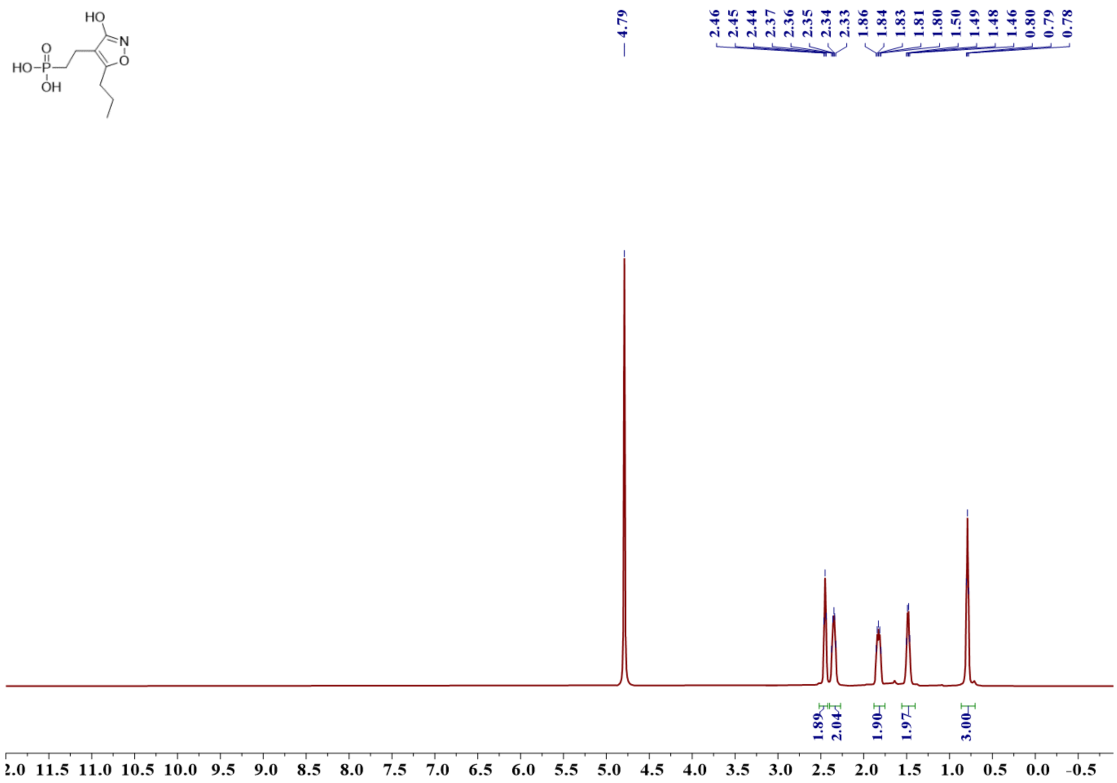

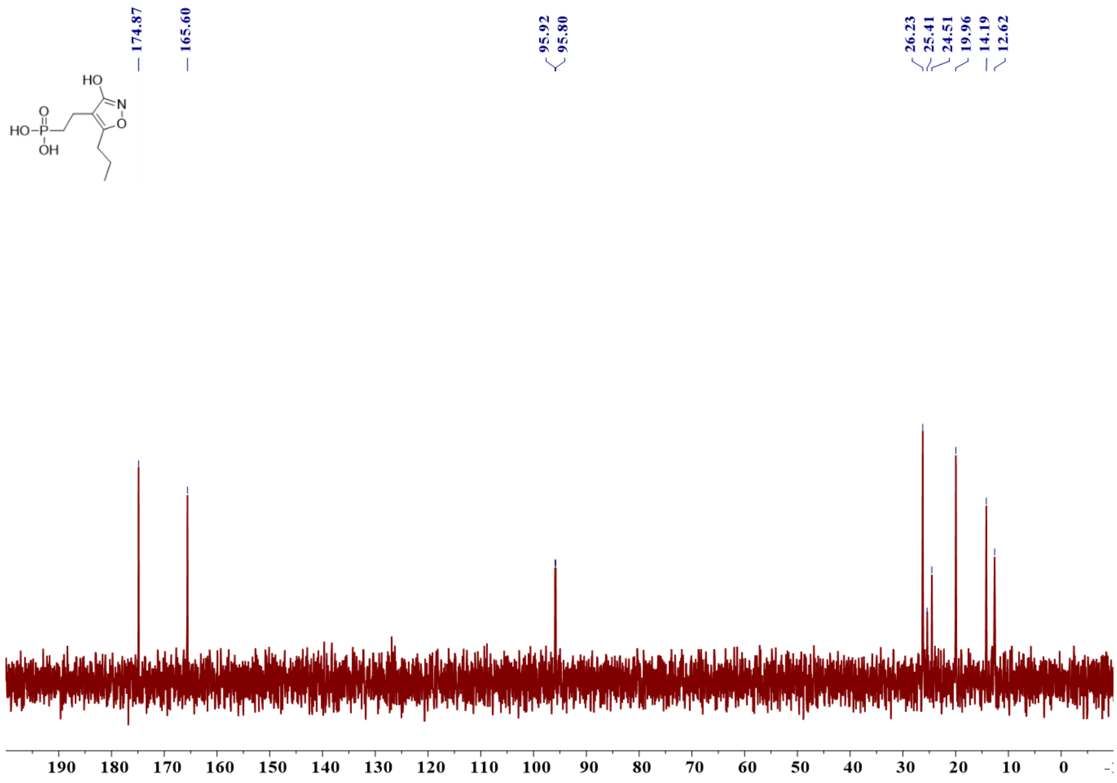

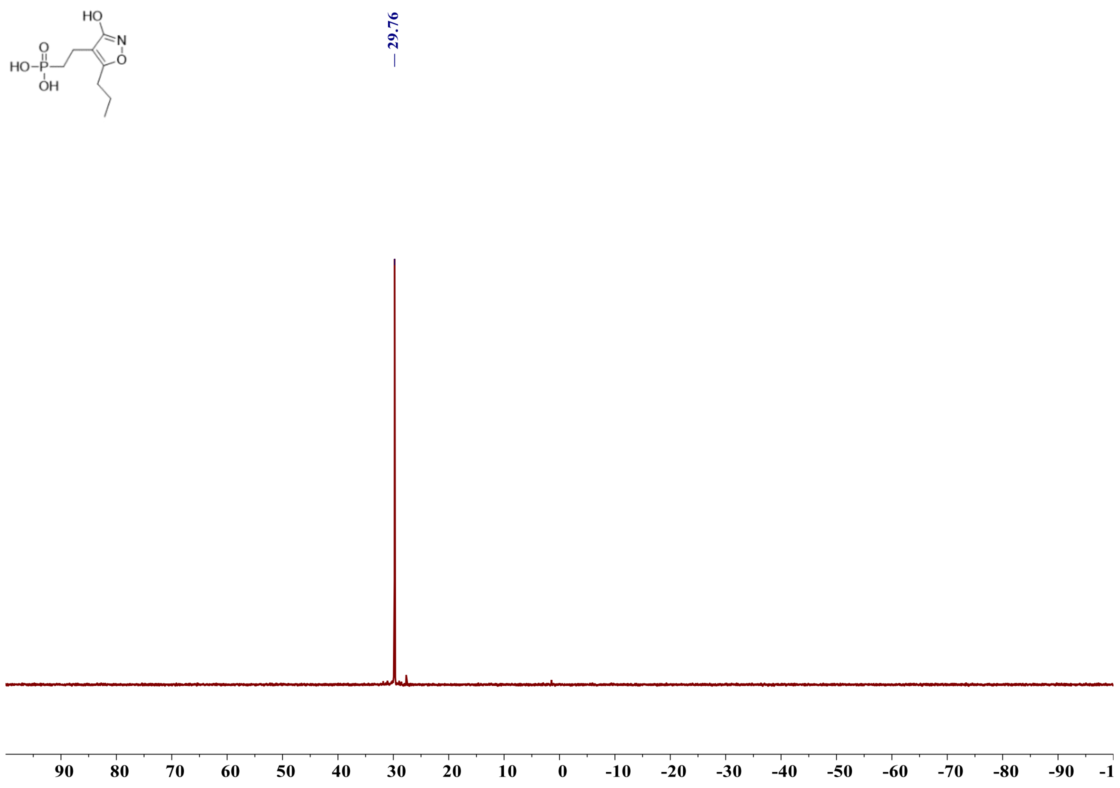
HO
N
O
HO
P
O
OH
29.76
90 80 70 60 50 40 30 20 10 0 -10 -20 -30 -40 -50 -60 -70 -80 -90 -1

(2-(3-hydroxy-5-isopropylisoxazol-4-yl)ethyl)phosphonic acid (**6d**)

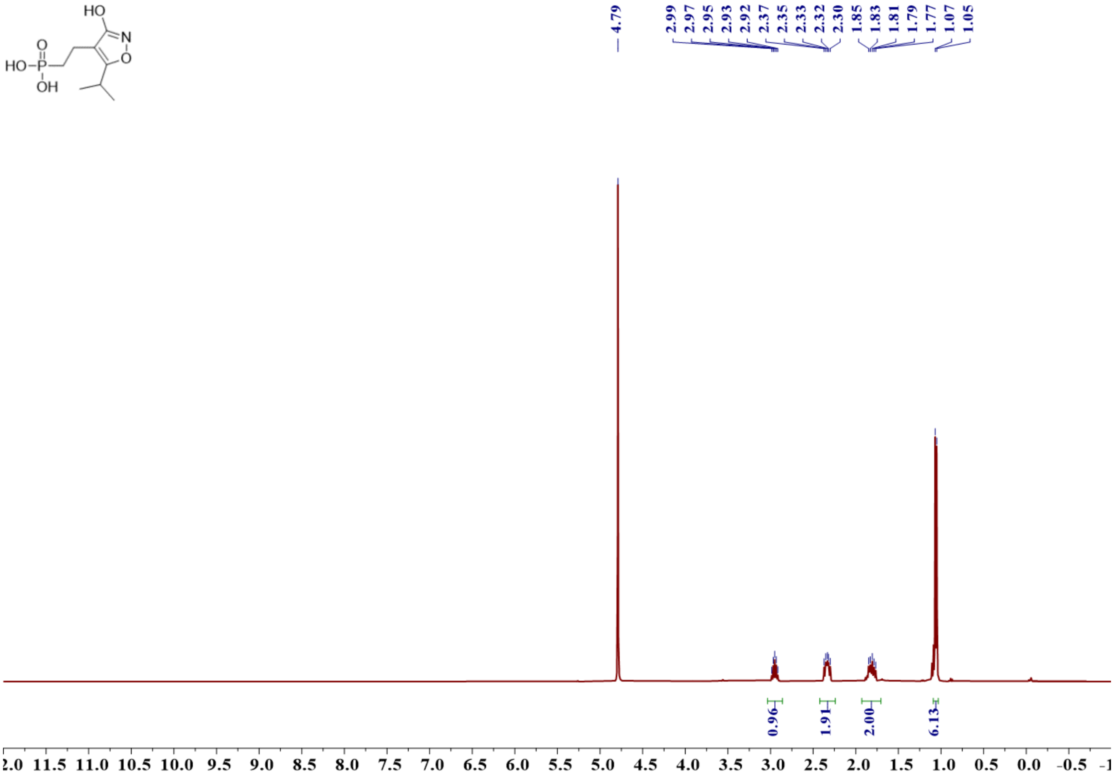

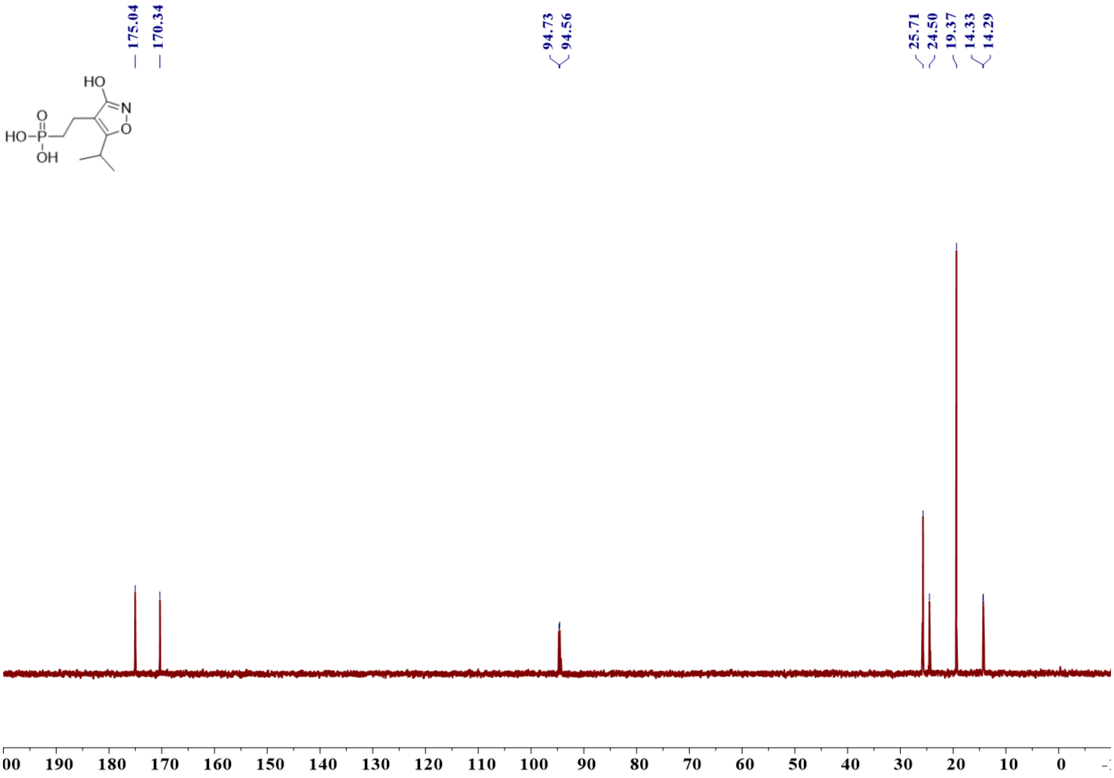

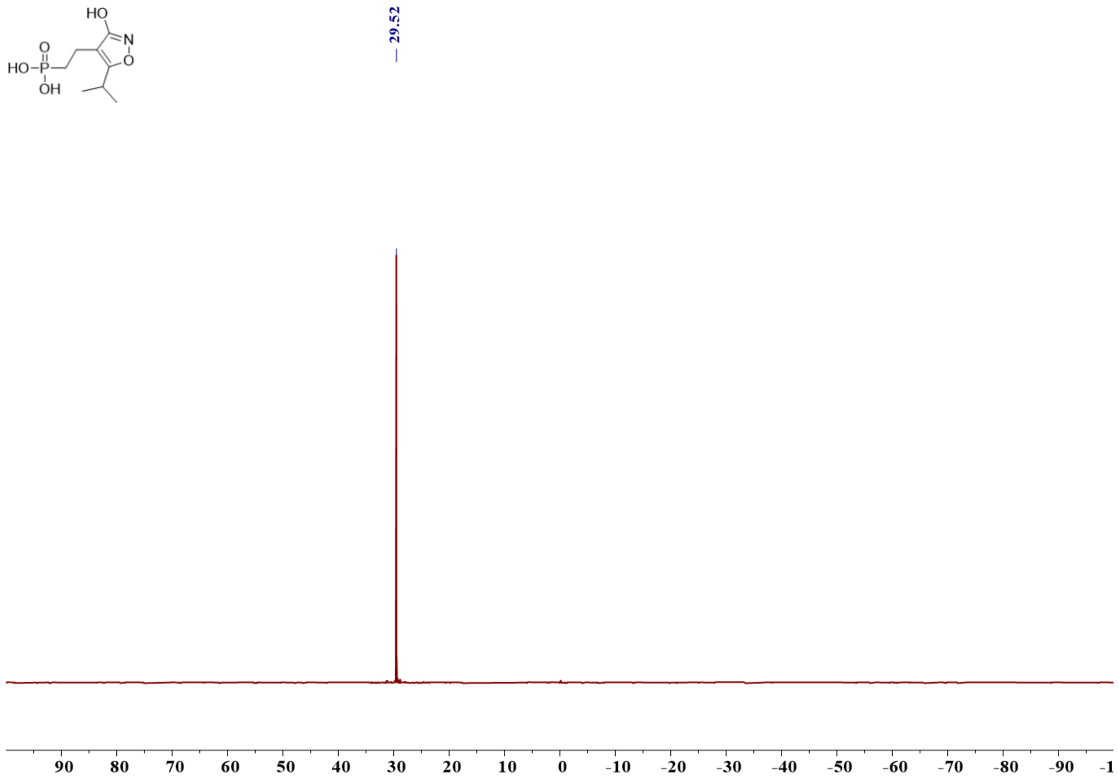

HO
N
O
O
HO
P
OH
29.52
90
80
70
60
50
40
30
20
10
0
-10
-20
-30
-40
-50
-60
-70
-80
-90
-1

(2-(3,5-dimethylisoxazol-4-yl)ethyl)phosphonic acid (**6e**)

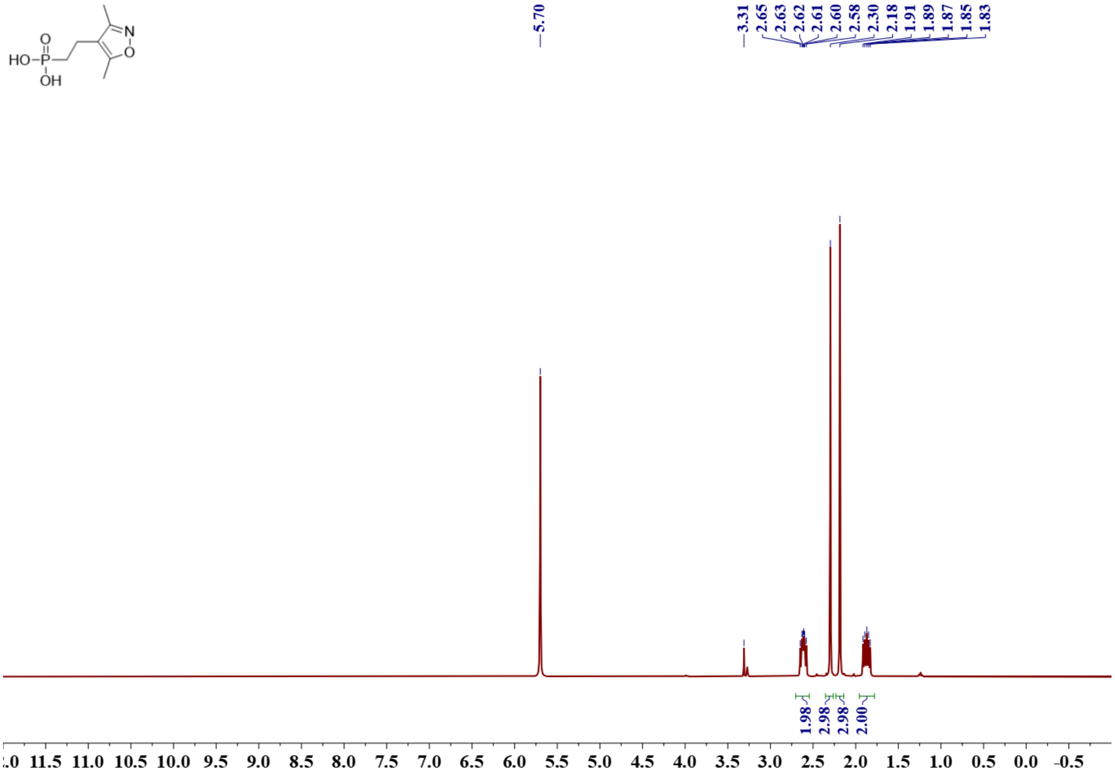

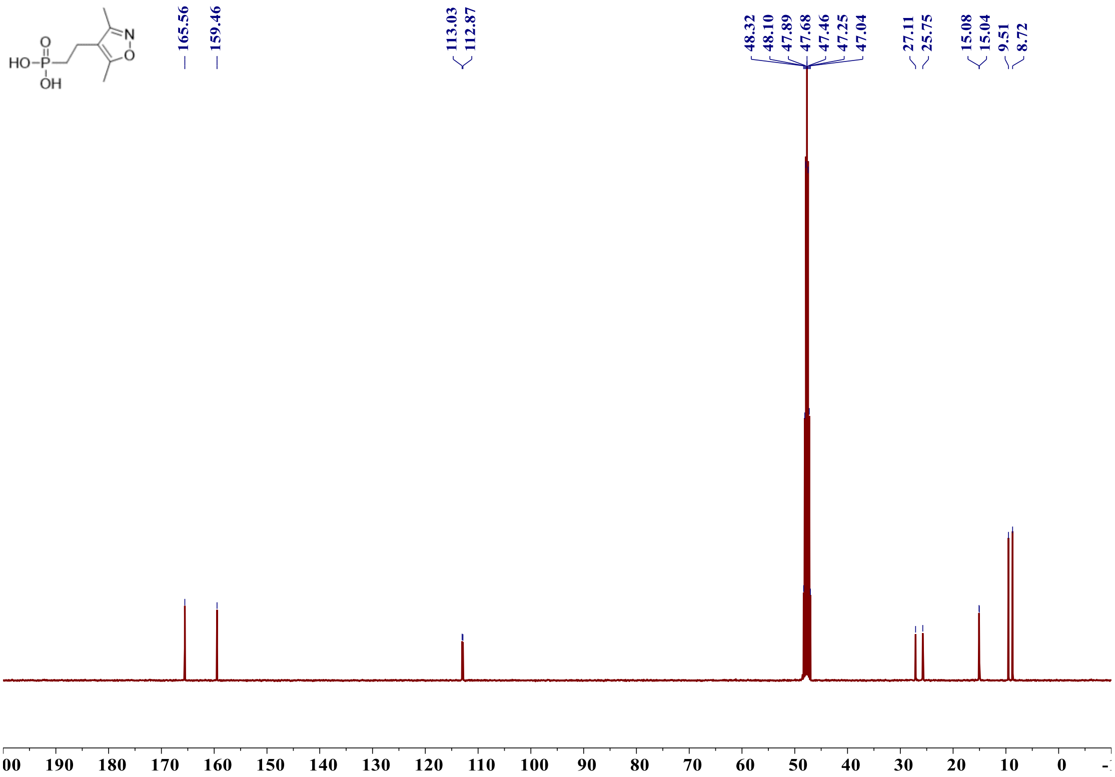

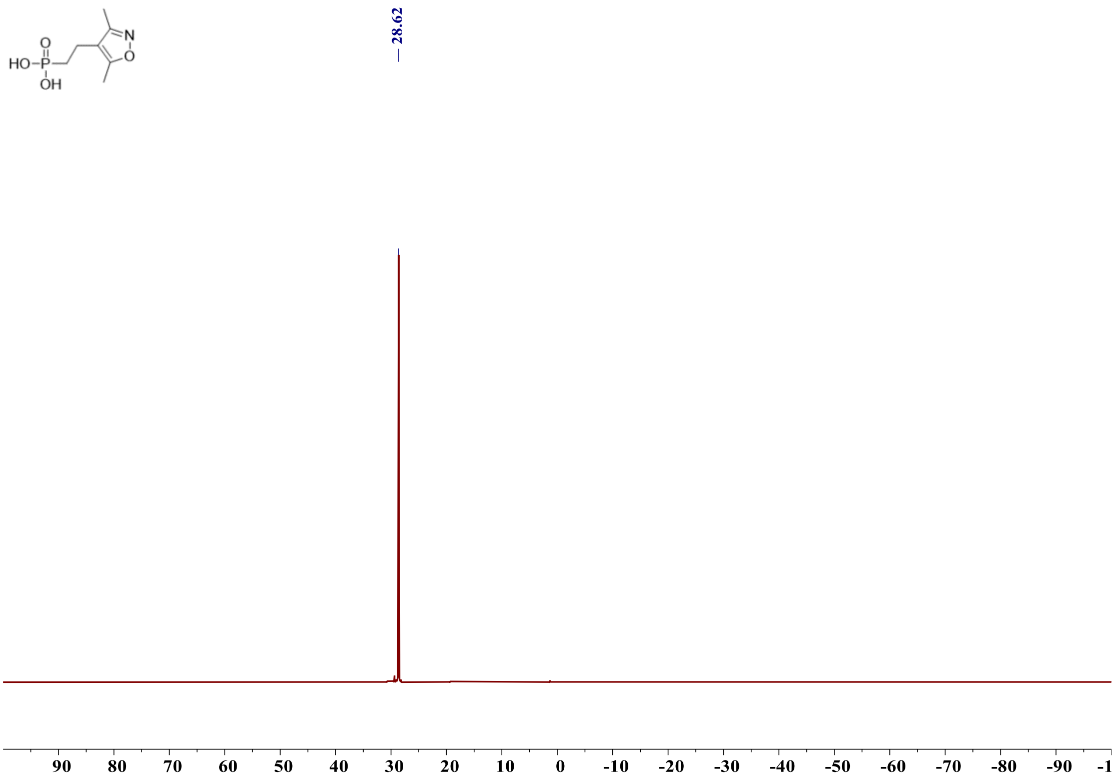
HO
O
P
OH
N
O
28.62
90 80 70 60 50 40 30 20 10 0 -10 -20 -30 -40 -50 -60 -70 -80 -90 -1

(2-(3,5-diethylisoxazol-4-yl)ethyl)phosphonic acid (**6f**)

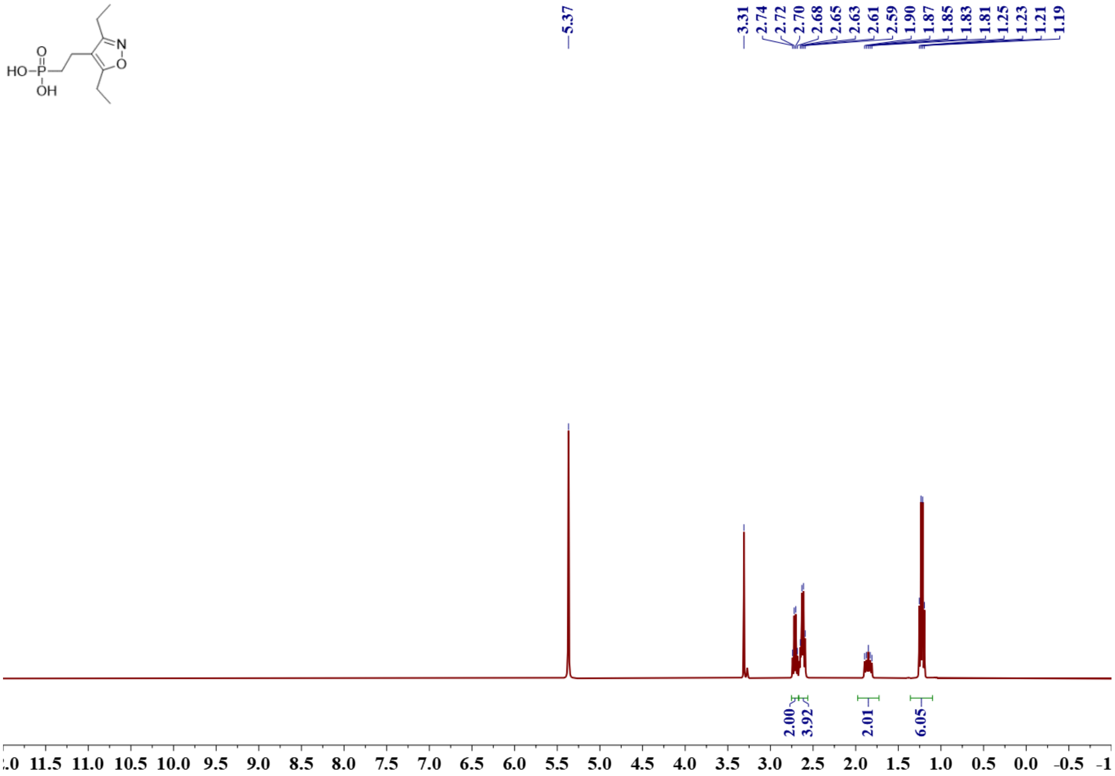

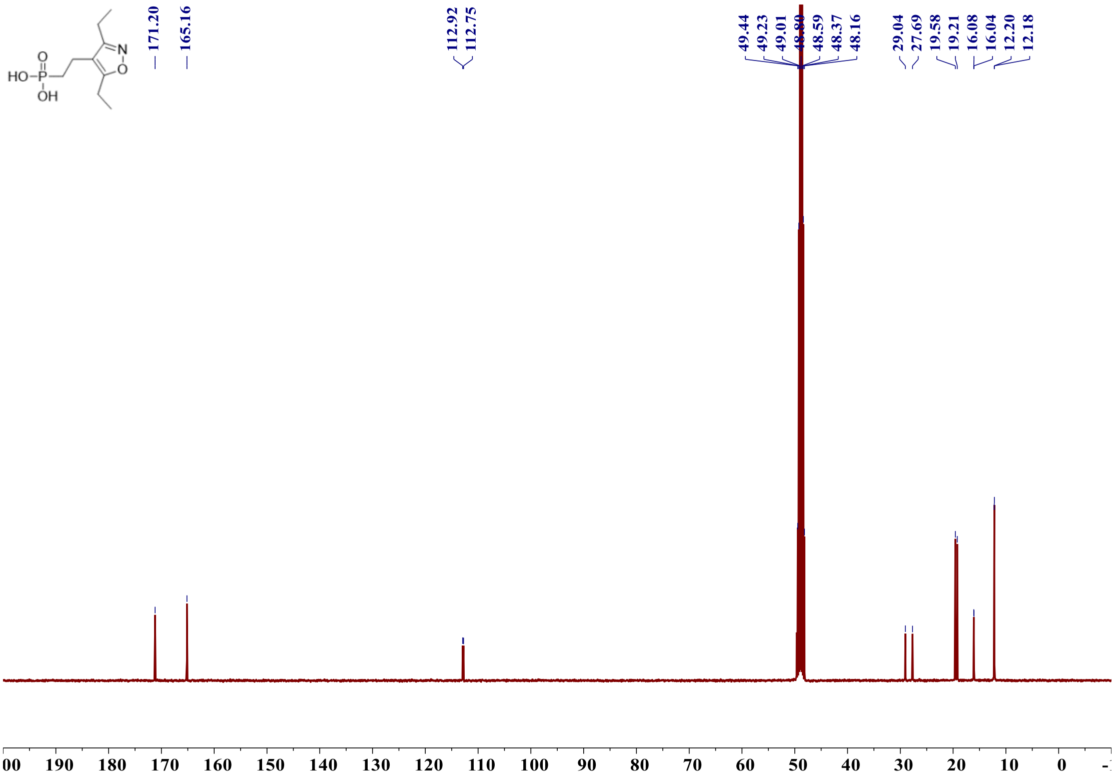

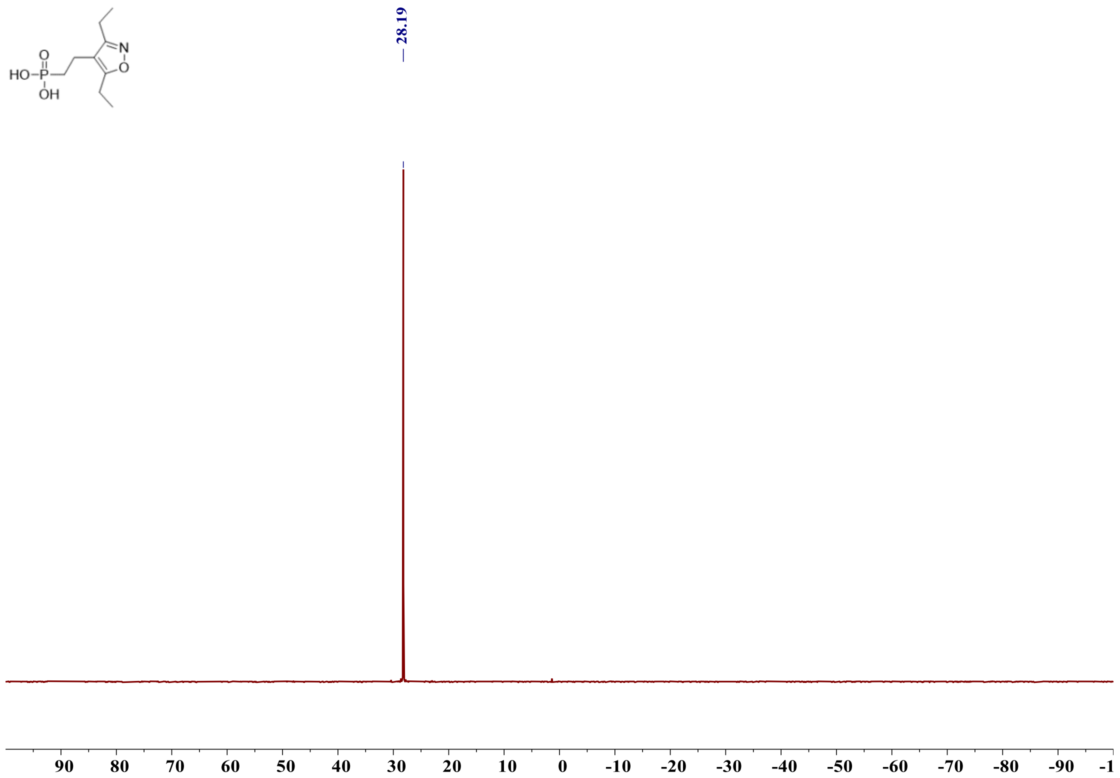
HO
OH
O
P
N
O
28.19
90
80
70
60
50
40
30
20
10
0
-10
-20
-30
-40
-50
-60
-70
-80
-90
-1

(2-(5-hydroxy-3-methyl-1H-pyrazol-4-yl)ethane-1,1-diyl)bis(phosphonic acid) (**12a**)

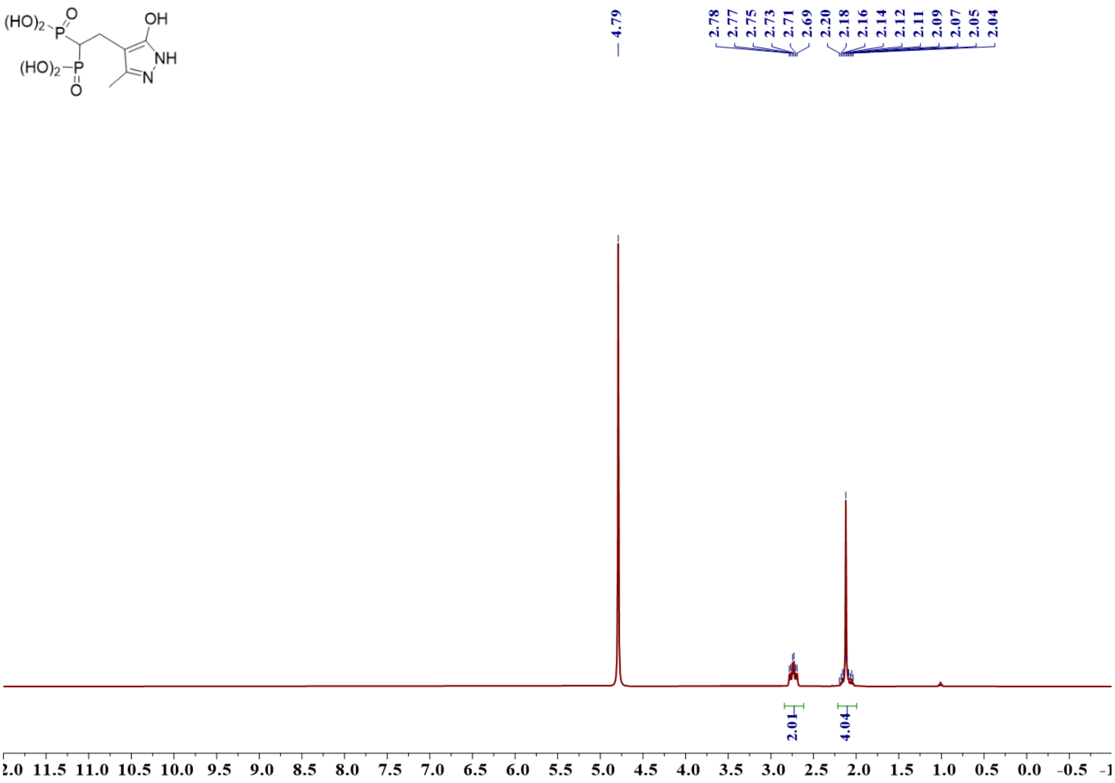

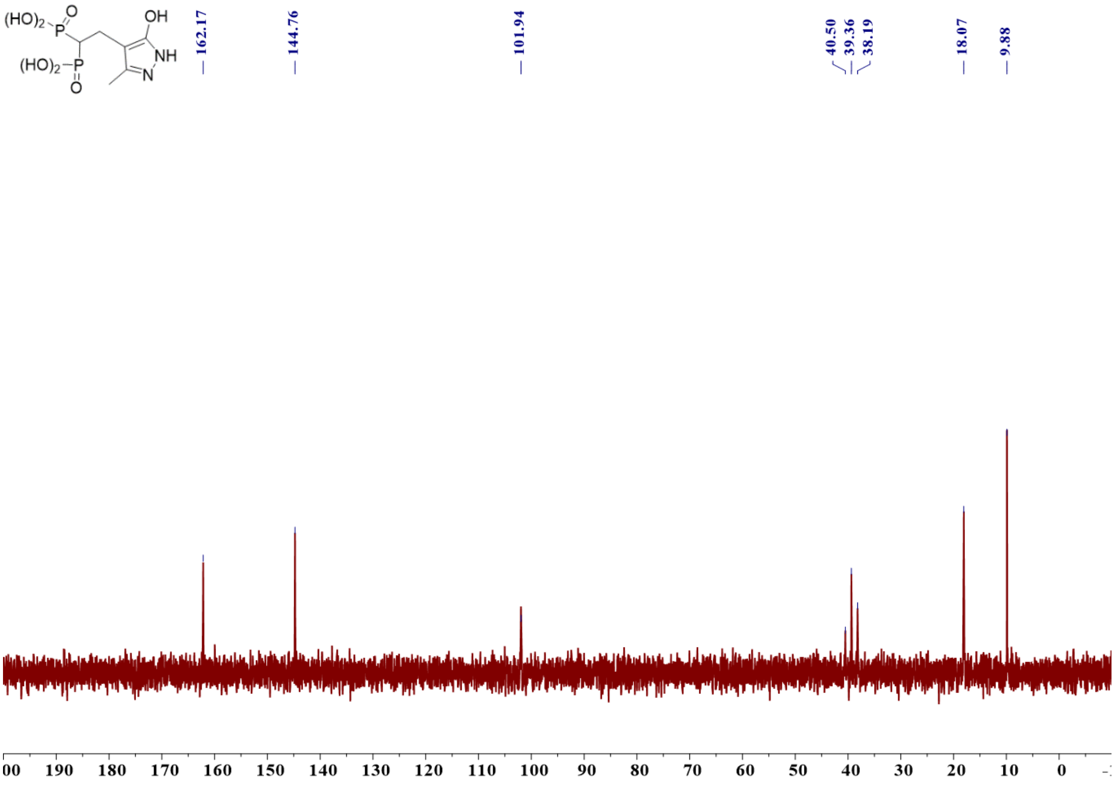

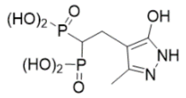

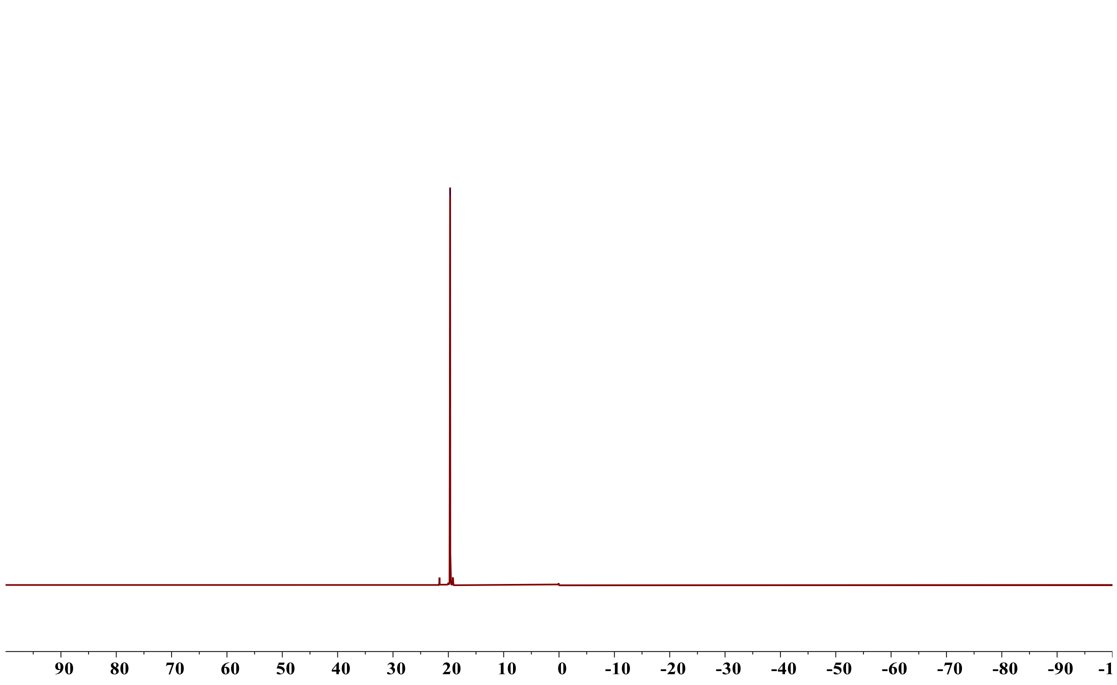
— 19.67
90
80
70
60
50
40
30
20
10
0
-10
-20
-30
-40
-50
-60
-70
-80
-90
-1

(2-(3-ethyl-5-hydroxy-1H-pyrazol-4-yl)ethane-1,1-diyl)bis(phosphonic acid) (**12b**)

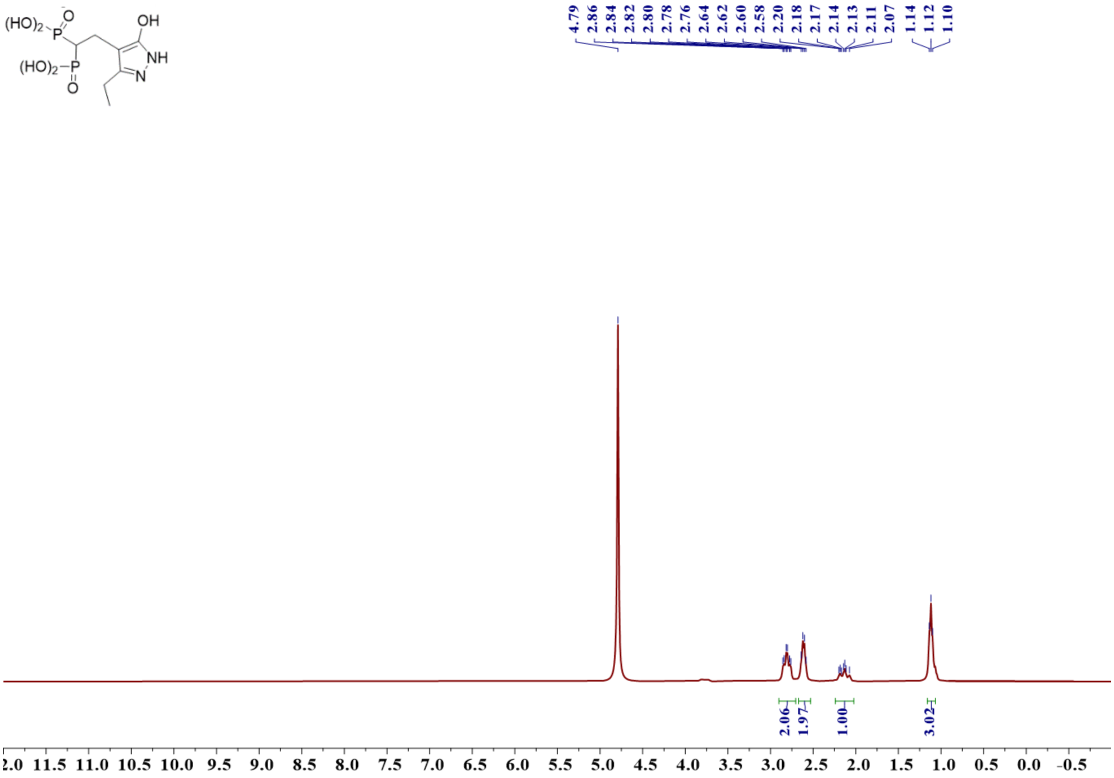

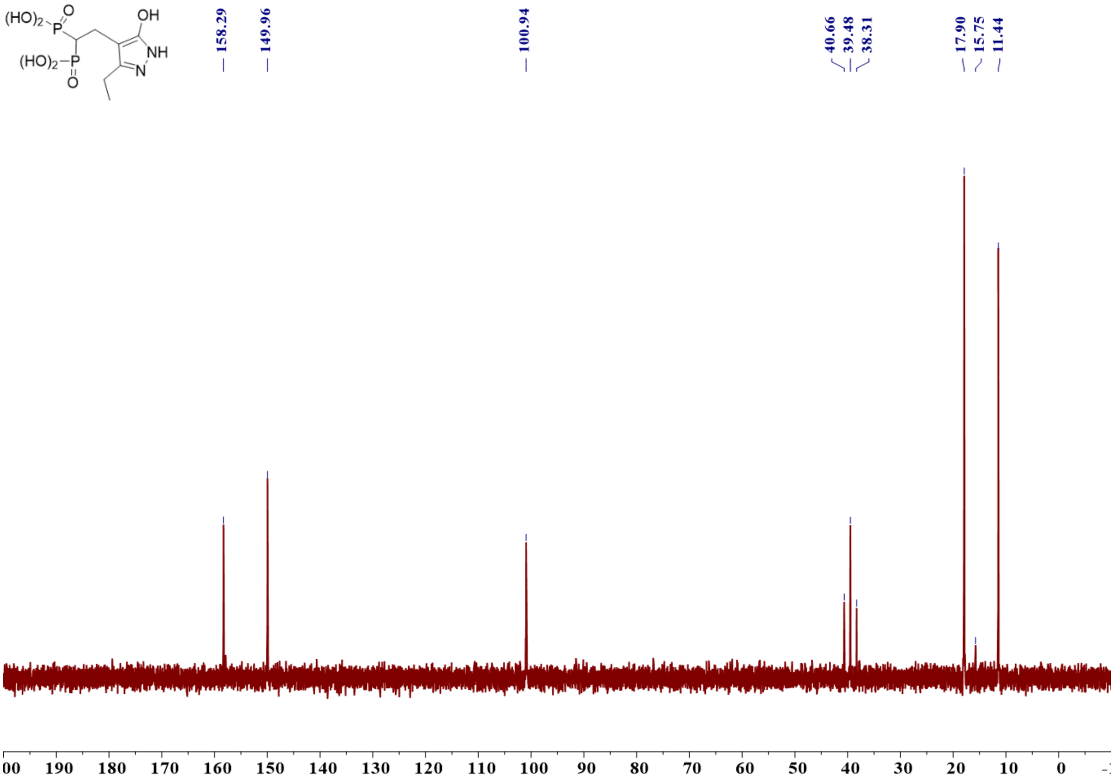

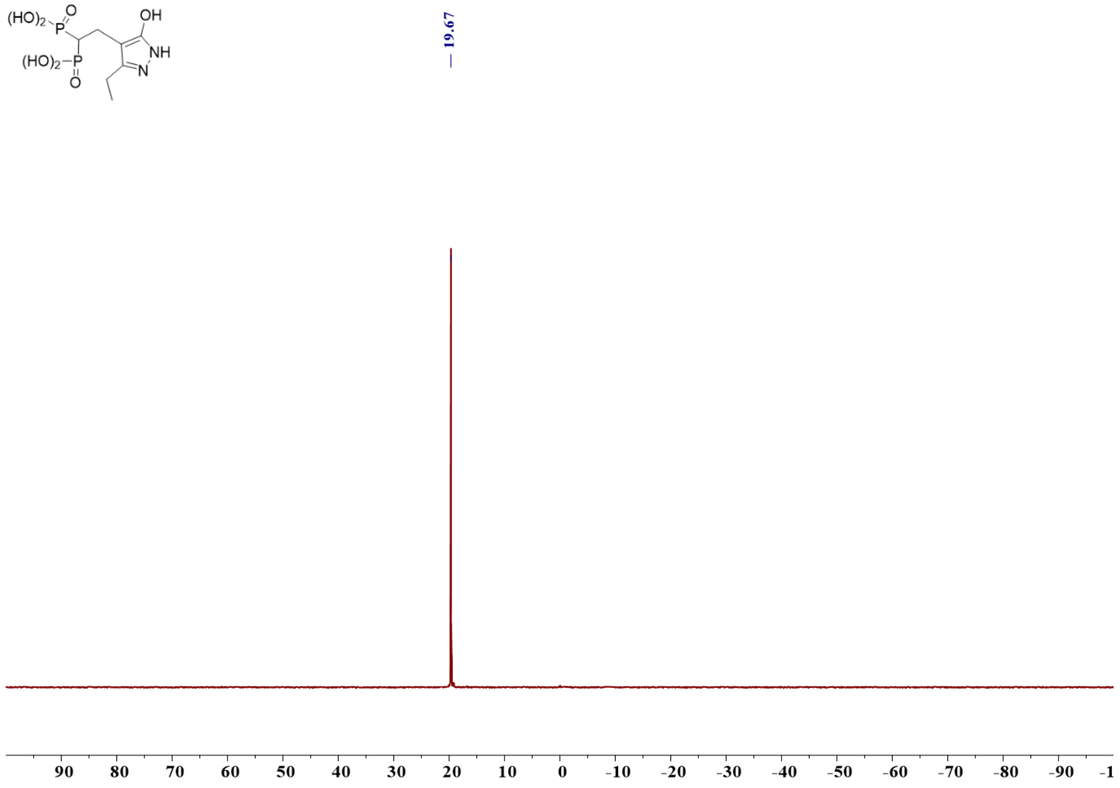

(HO)2 P O
(HO)2 P O
OH
NH
N
19.67
90 80 70 60 50 40 30 20 10 0 -10 -20 -30 -40 -50 -60 -70 -80 -90 -1

(2-(5-hydroxy-3-propyl-1H-pyrazol-4-yl)ethane-1,1-diyl)bis(phosphonic acid) (**12c**)

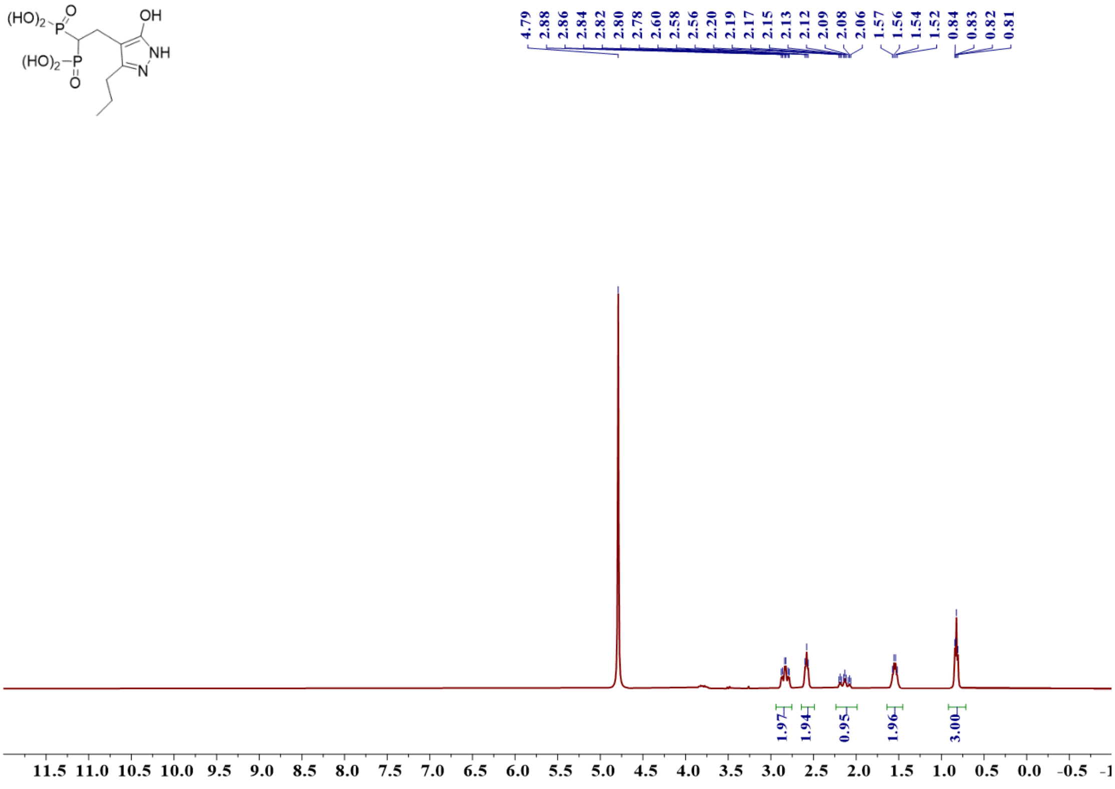

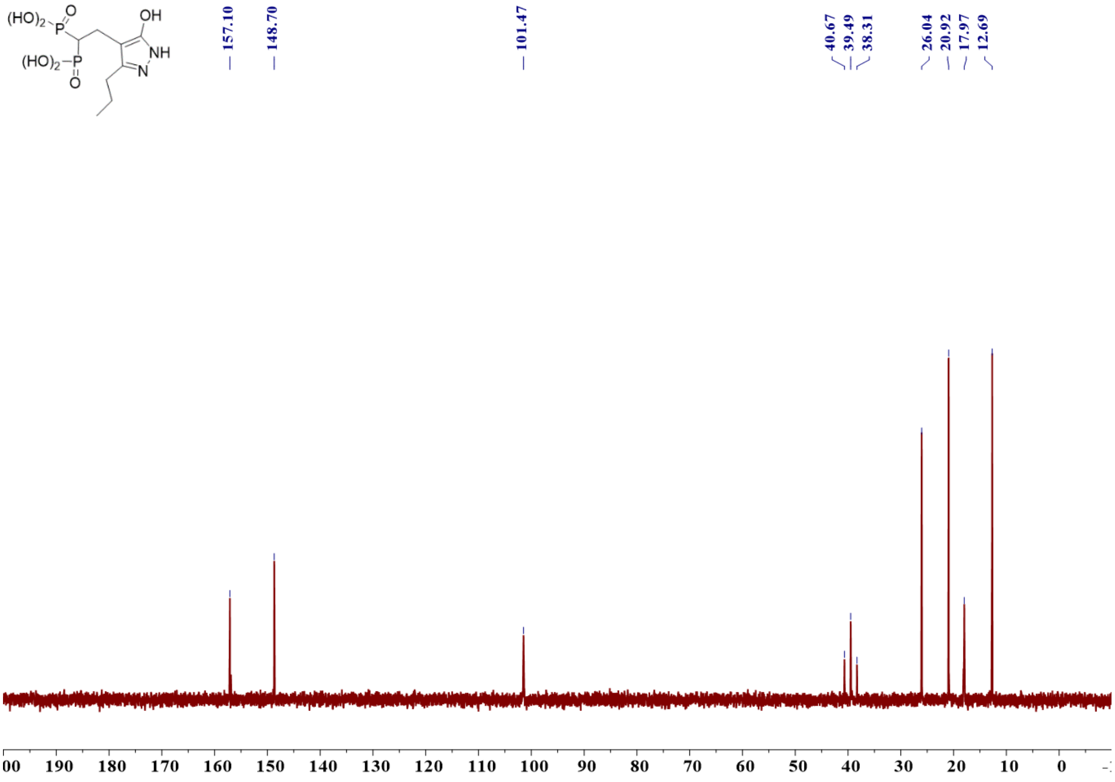

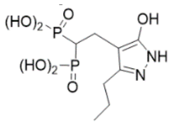

(HO)2
O
P
(HO)2
P
O
OH
NH
N

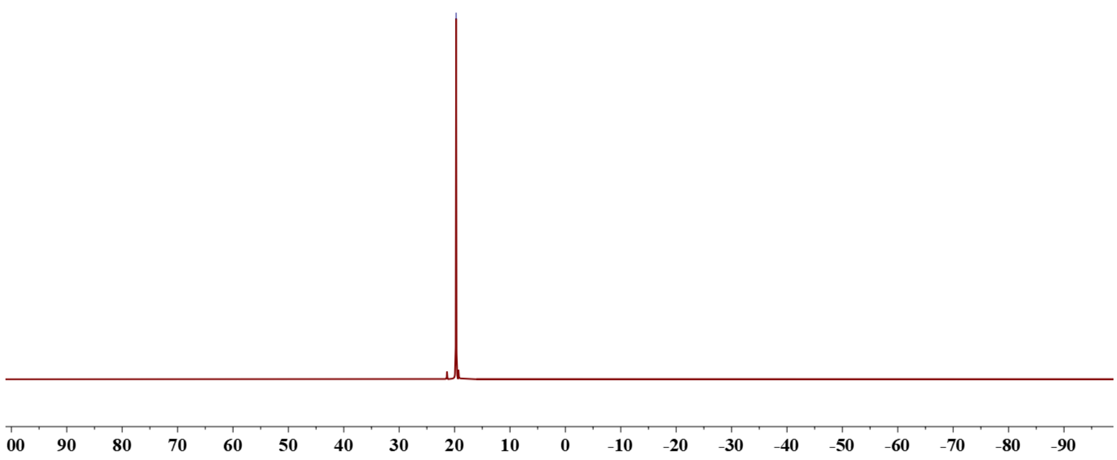

19.72
00
90
80
70
60
50
40
30
20
10
0
-10
-20
-30
-40
-50
-60
-70
-80
-90

(2-(5-hydroxy-3-isopropyl-1H-pyrazol-4-yl)ethane-1,1-diyl)bis(phosphonic acid) (**12d**)

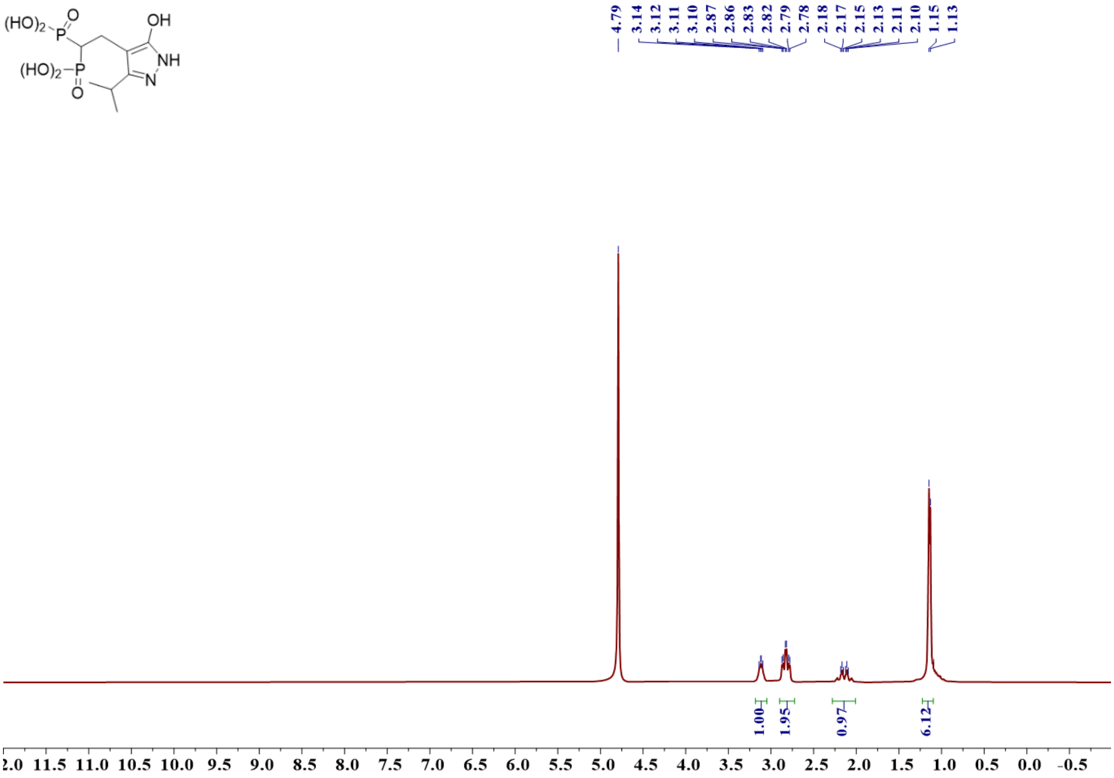

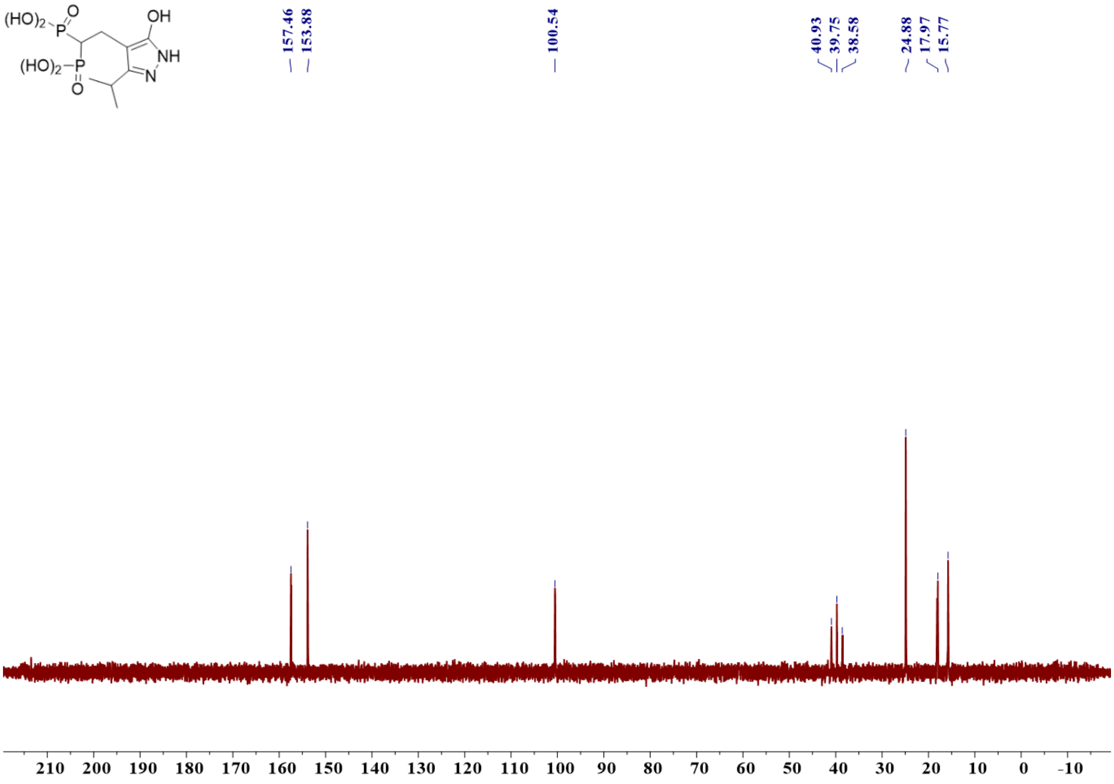

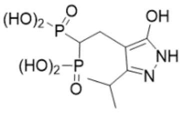

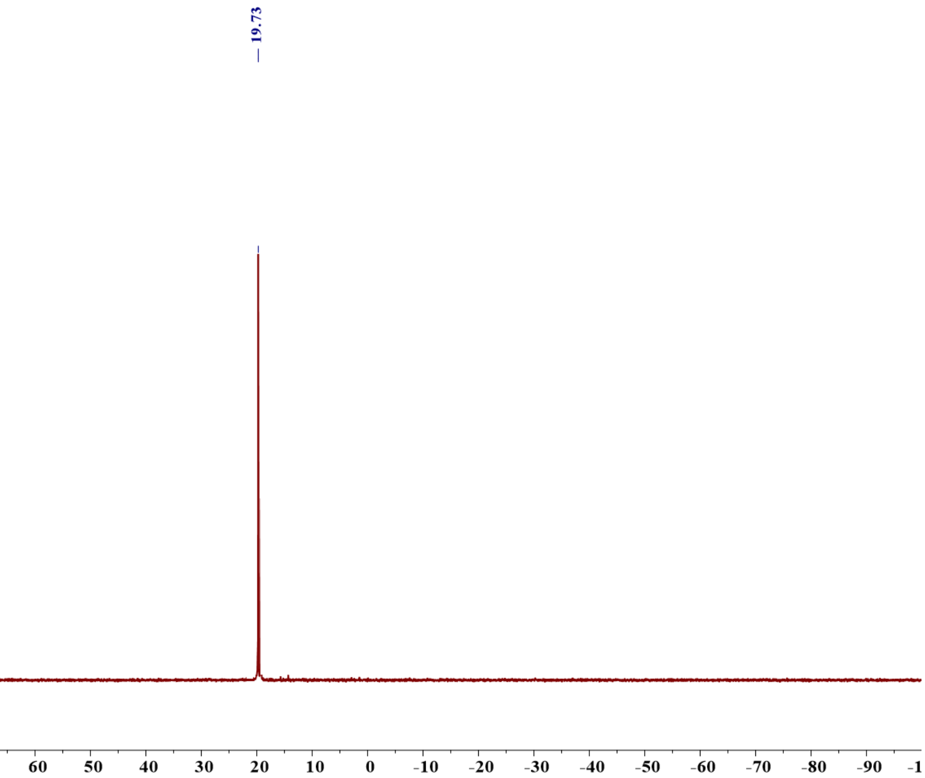

19.73
90
80
70
60
50
40
30
20
10
0
-10
-20
-30
-40
-50
-60
-70
-80
-90

(2-(5-hydroxy-3-(trifluoromethyl)-1H-pyrazol-4-yl)ethane-1,1-diyl)bis(phosphonic acid) (**12e**)

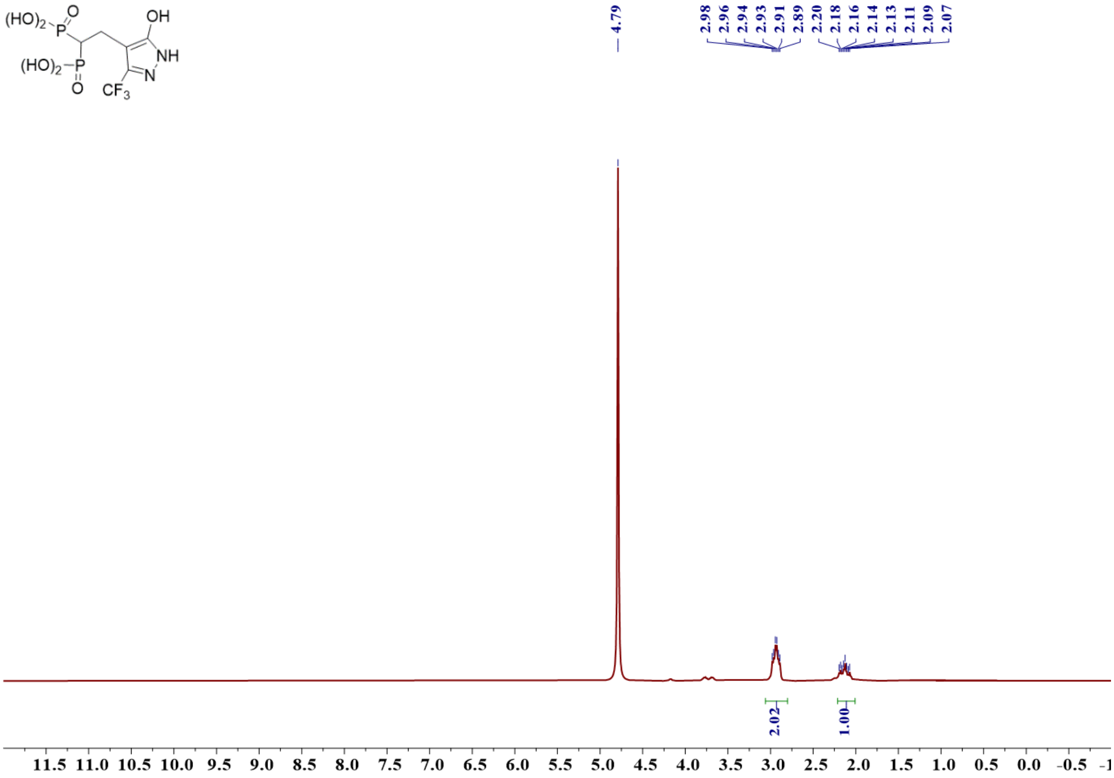

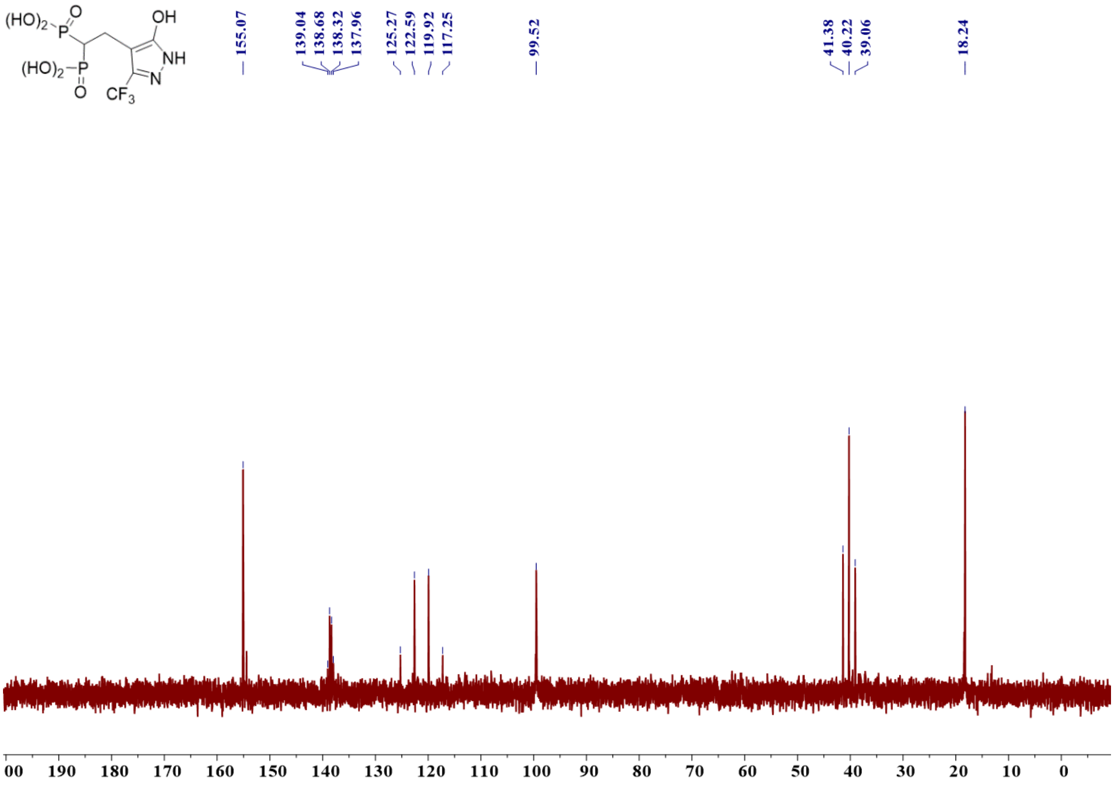

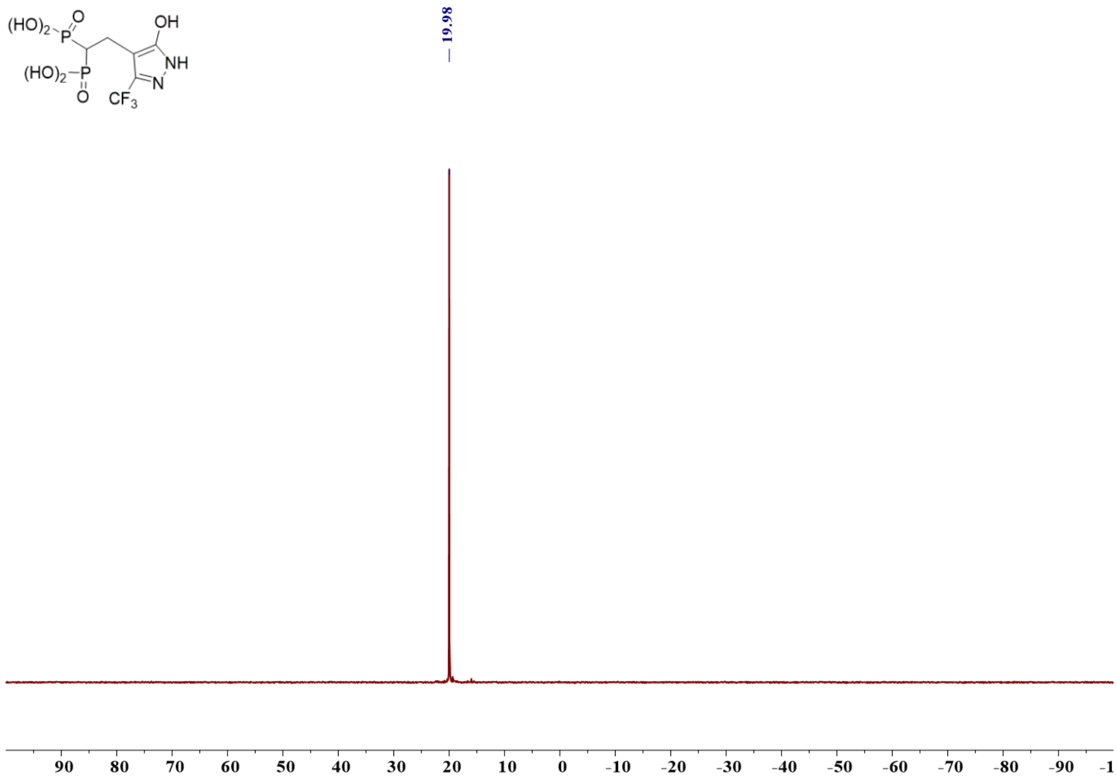

(HO)2 P O
(HO)2 P O
OH
NH
N
CF3
19.98
90 80 70 60 50 40 30 20 10 0 -10 -20 -30 -40 -50 -60 -70 -80 -90 -1

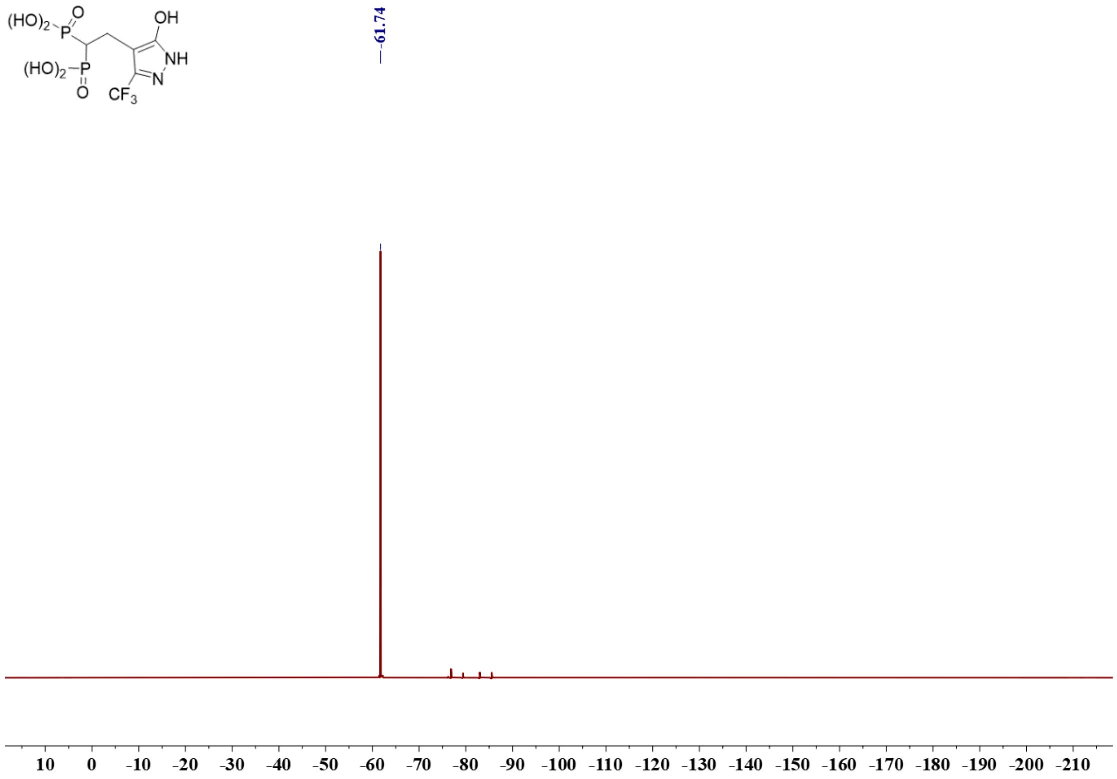

(HO)2 P O
(HO)2 P O
OH
NH
N
CF3
-61.74
10 0 -10 -20 -30 -40 -50 -60 -70 -80 -90 -100 -110 -120 -130 -140 -150 -160 -170 -180 -190 -200 -210

(2-(3-(difluoromethyl)-5-hydroxy-1H-pyrazol-4-yl)ethane-1,1-diyl)bis(phosphonic acid) (**12f**)

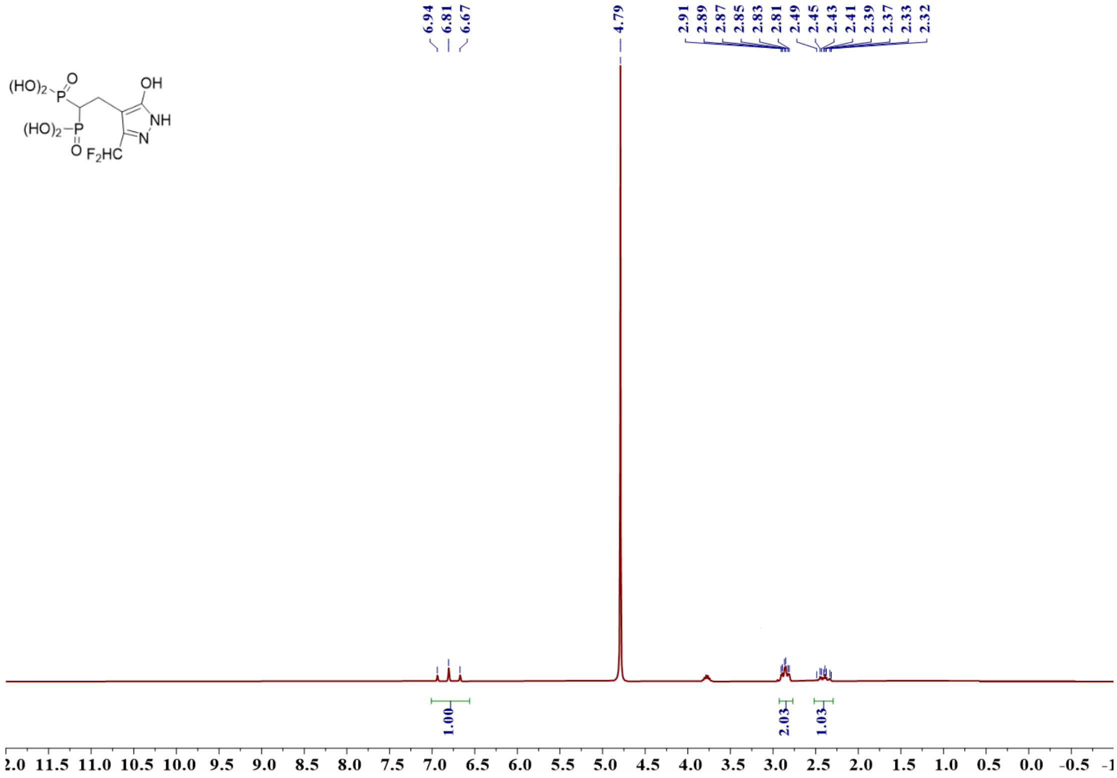

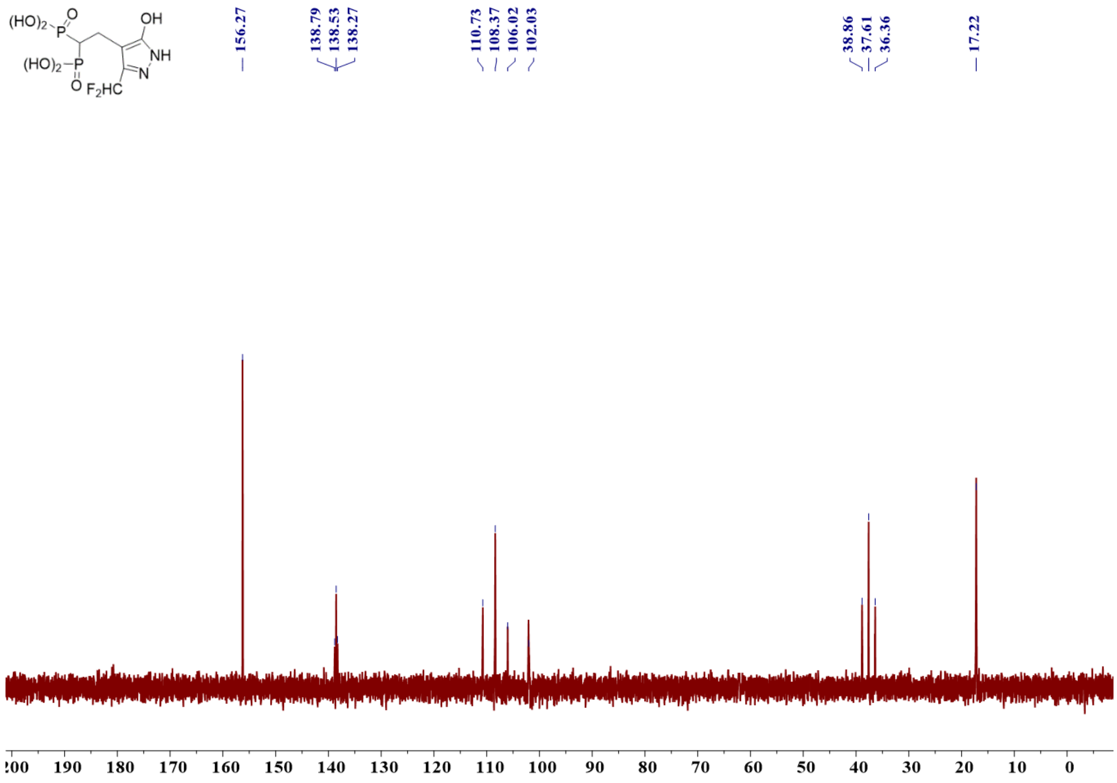

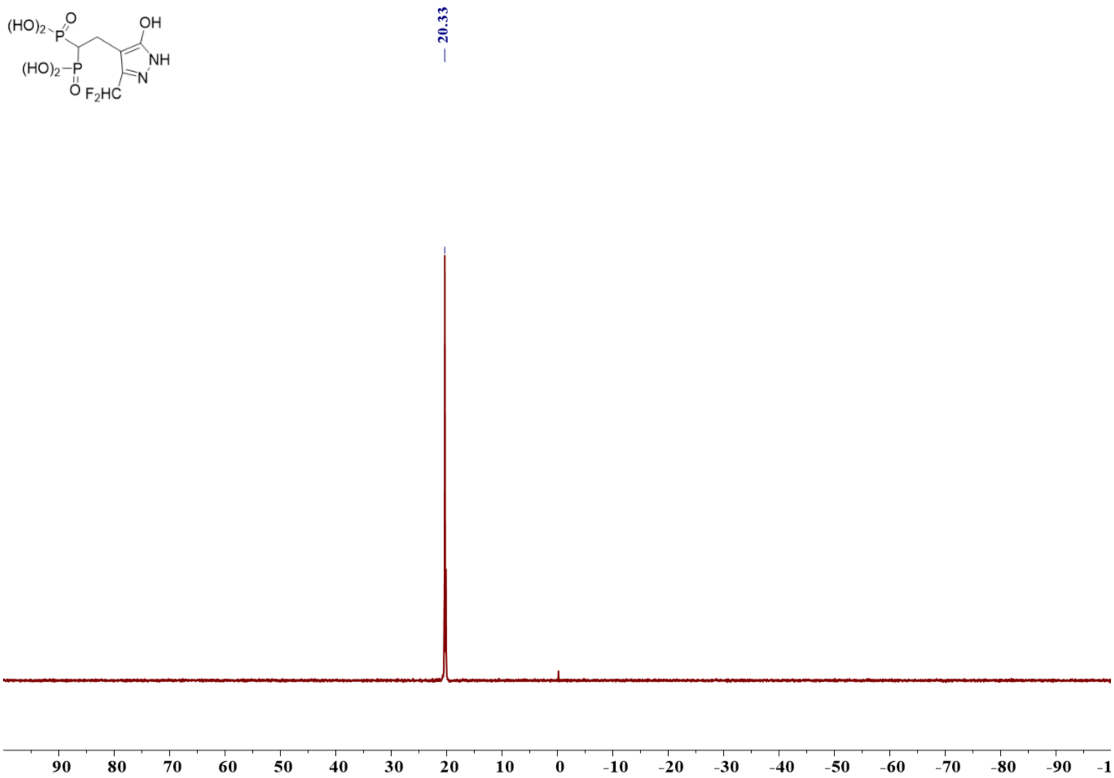

(HO)2
O
P
(HO)2
P
O
OH
NH
N
F2HC
20.33
90
80
70
60
50
40
30
20
10
0
-10
-20
-30
-40
-50
-60
-70
-80
-90

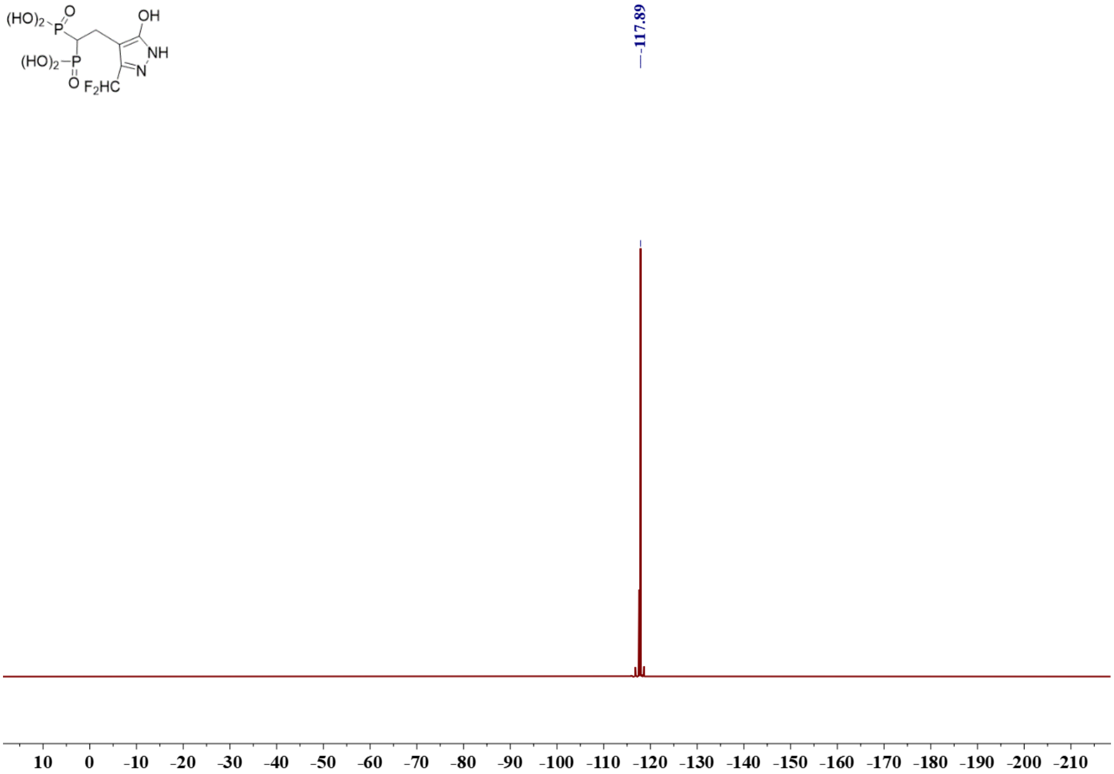

(HO)2
O
P
(HO)2
P
O
OH
NH
N
F2HC
-117.89
10
0
-10
-20
-30
-40
-50
-60
-70
-80
-90
-100
-110
-120
-130
-140
-150
-160
-170
-180
-190
-200
-210

(2-(3,5-dimethyl-1H-pyrazol-4-yl)ethane-1,1-diyl)bis(phosphonic acid) (**12g**)

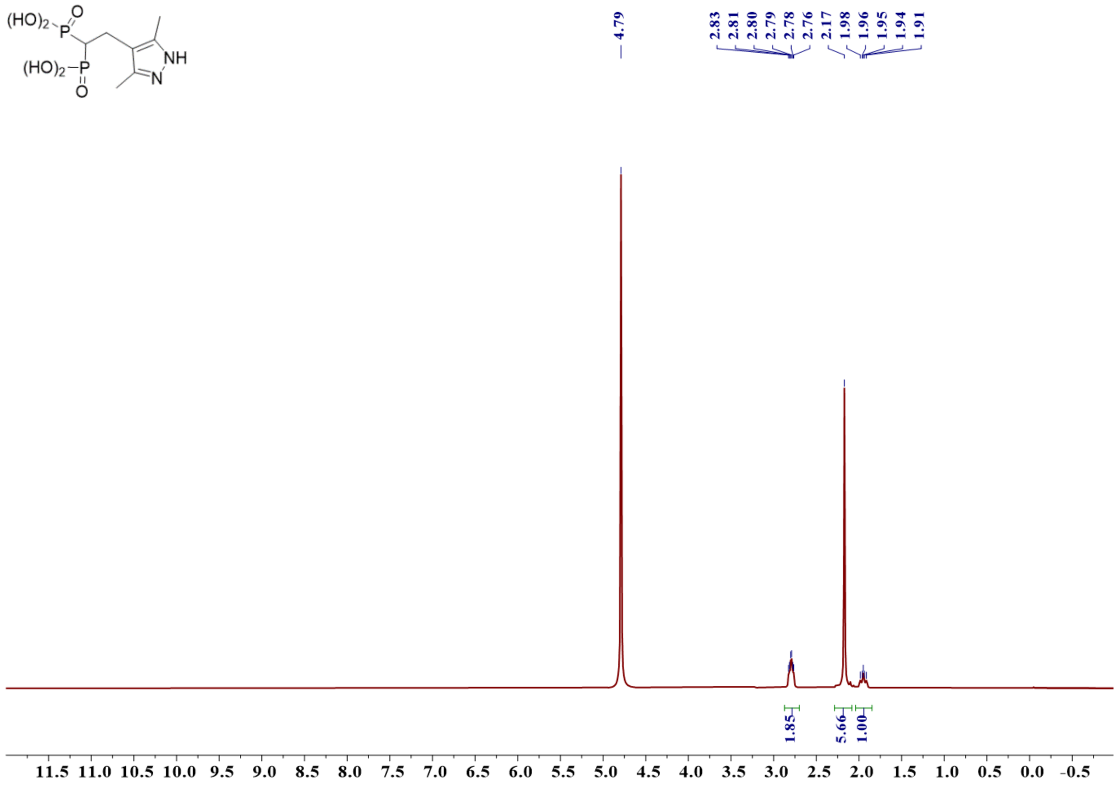

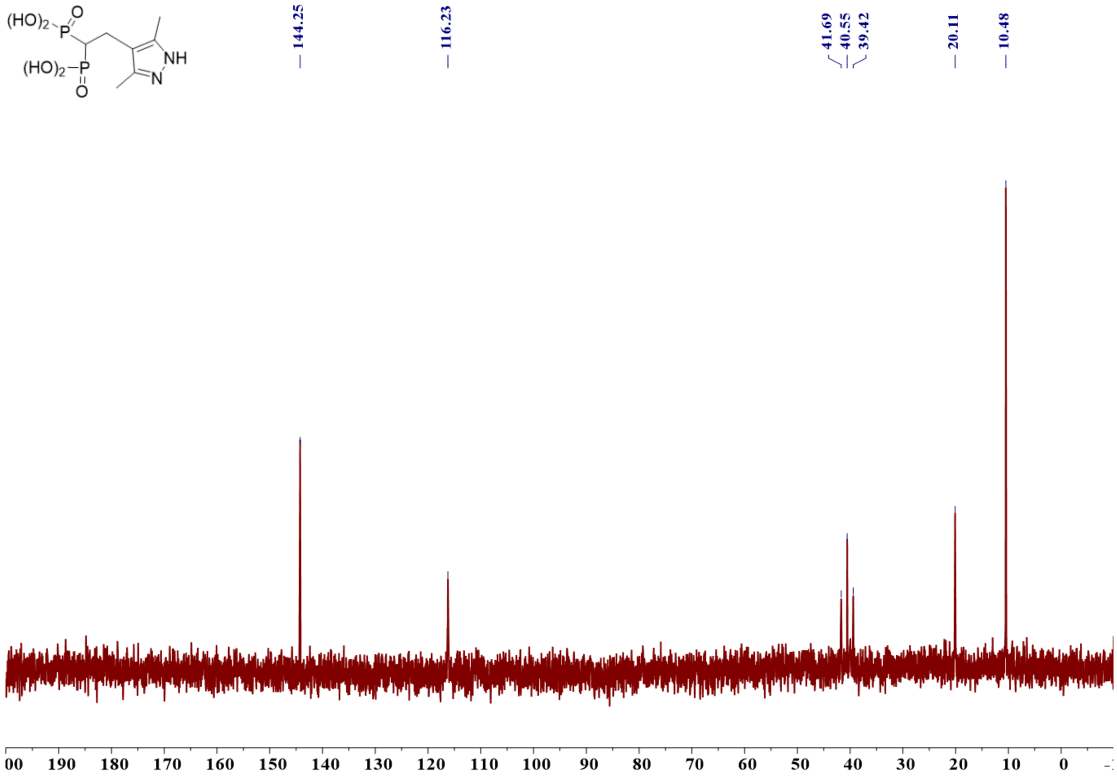

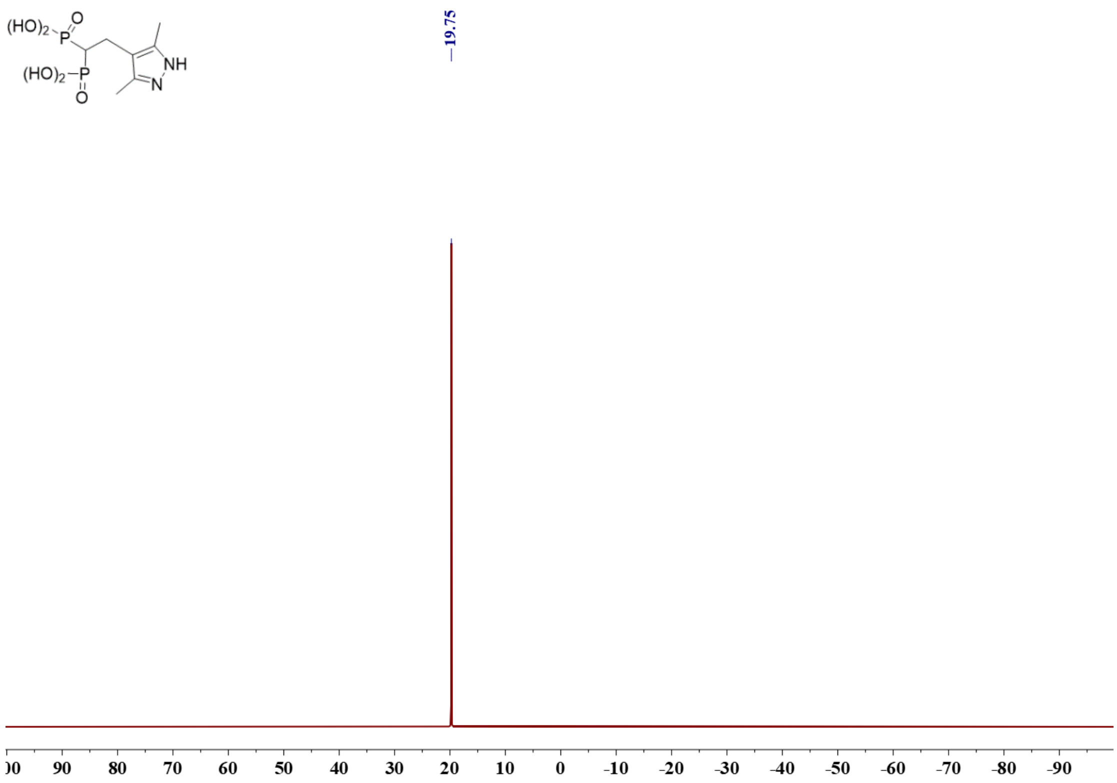
(HO)2
(HO)2
NH
N
19.75
00
90
80
70
60
50
40
30
20
10
0
-10
-20
-30
-40
-50
-60
-70
-80
-90

(2-(3,5-diethyl-1H-pyrazol-4-yl)ethane-1,1-diyl)bis(phosphonic acid) (**12h**)

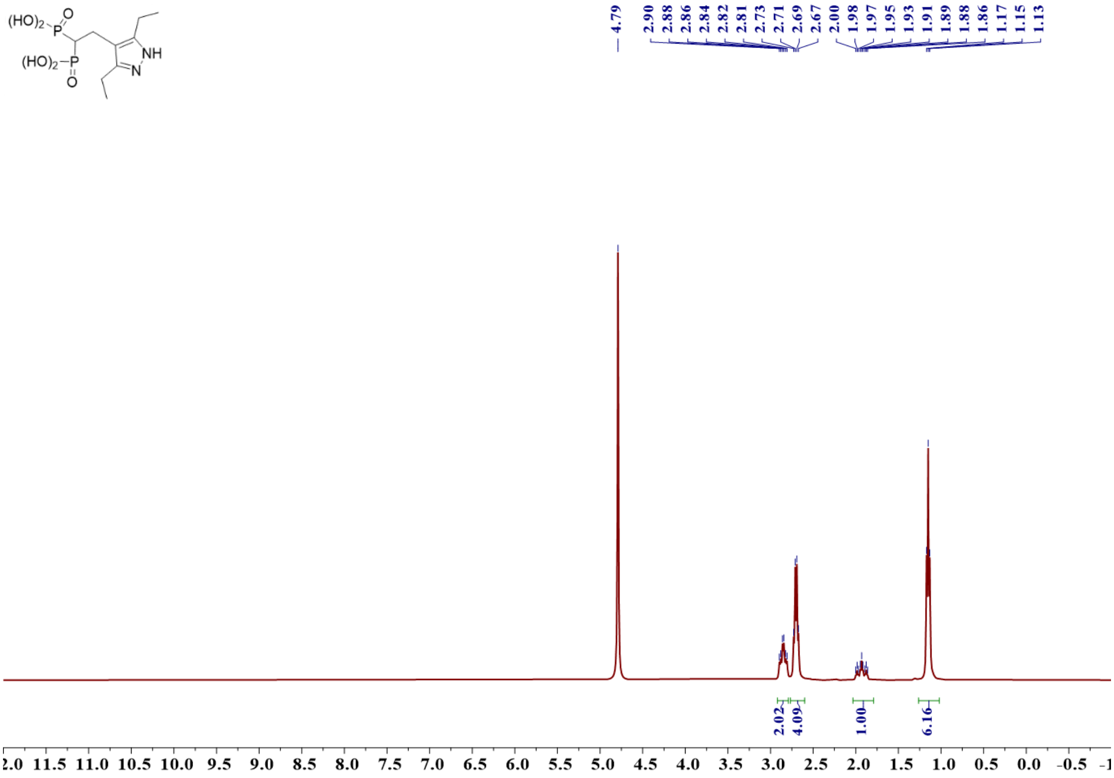

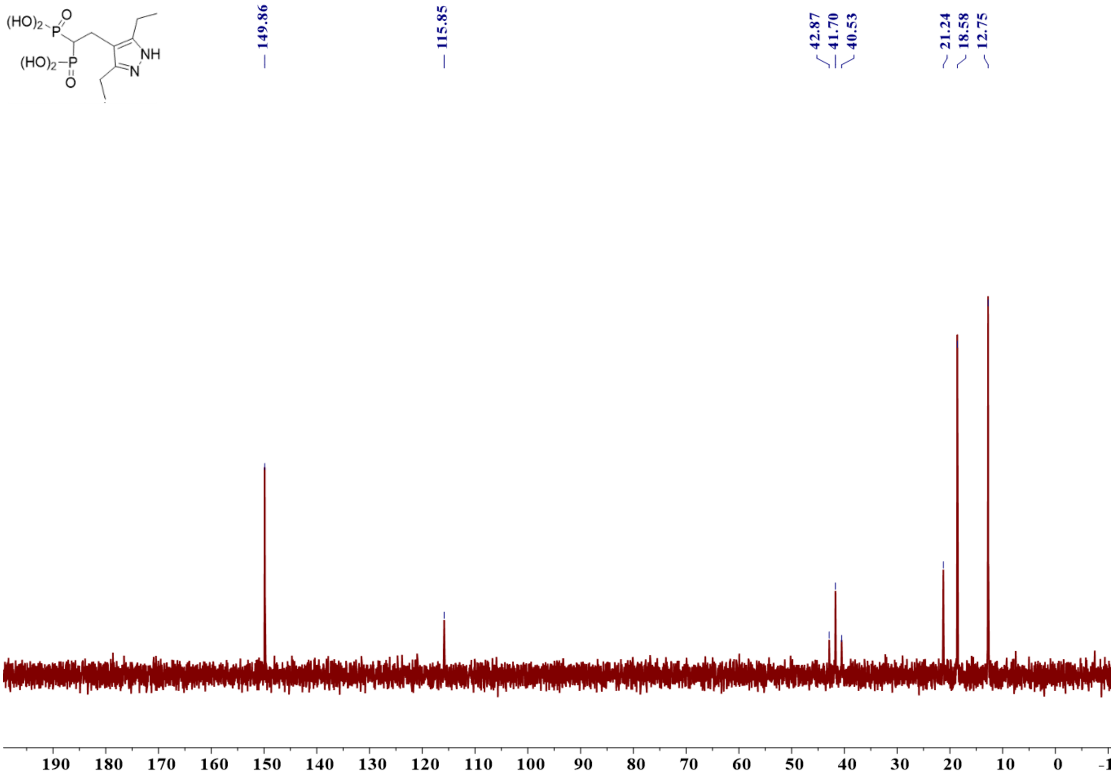

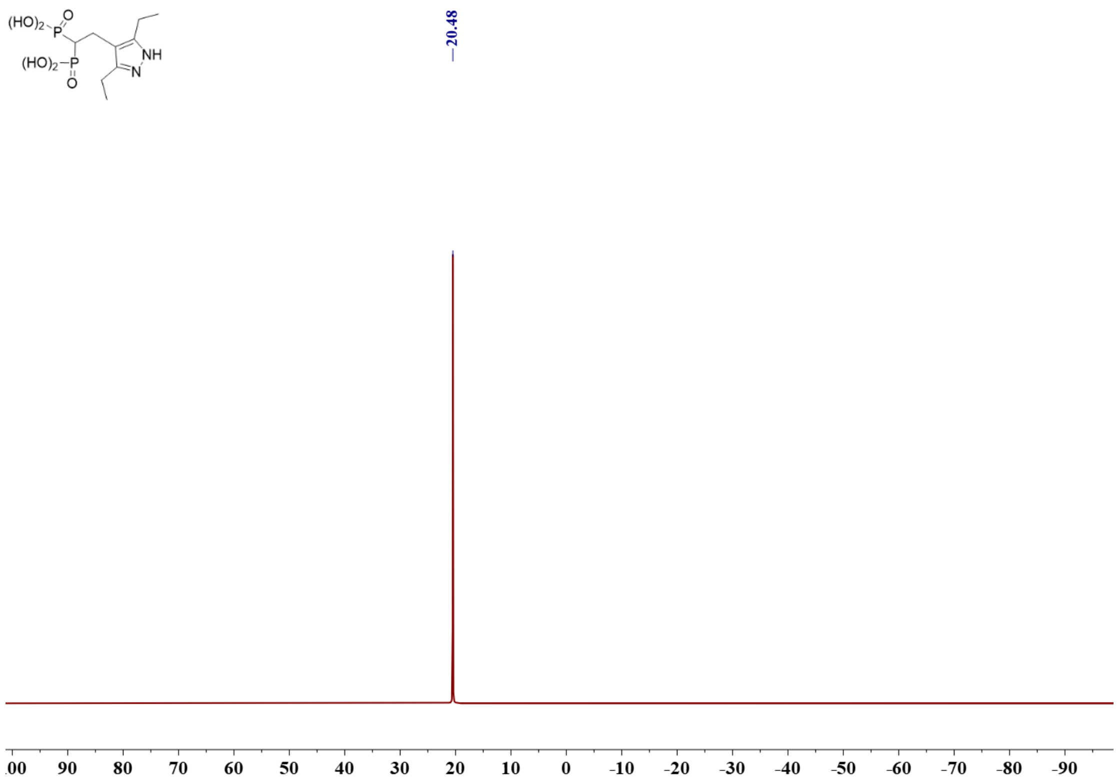
(HO)2
P
O
(HO)2
P
O
NH
N
20.48
.00 90 80 70 60 50 40 30 20 10 0 -10 -20 -30 -40 -50 -60 -70 -80 -90

(2-(3-methyl-5-(trifluoromethyl)-1H-pyrazol-4-yl)ethane-1,1-diyl)bis(phosphonic acid) (**12i**)

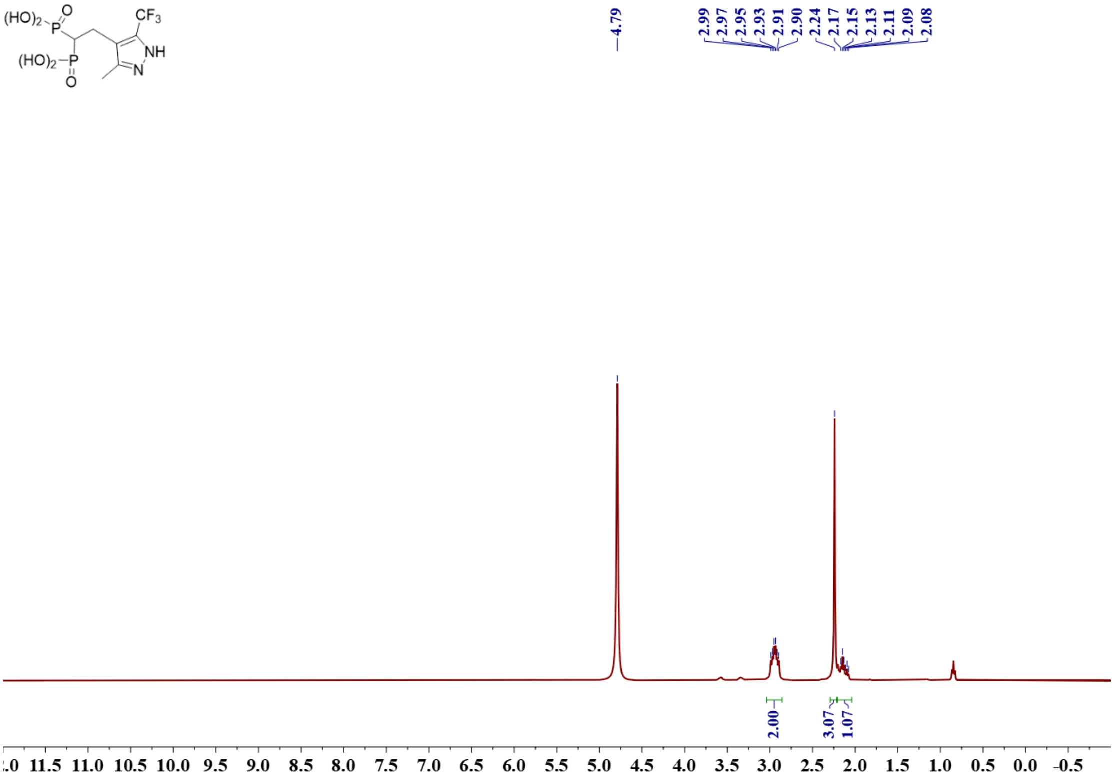

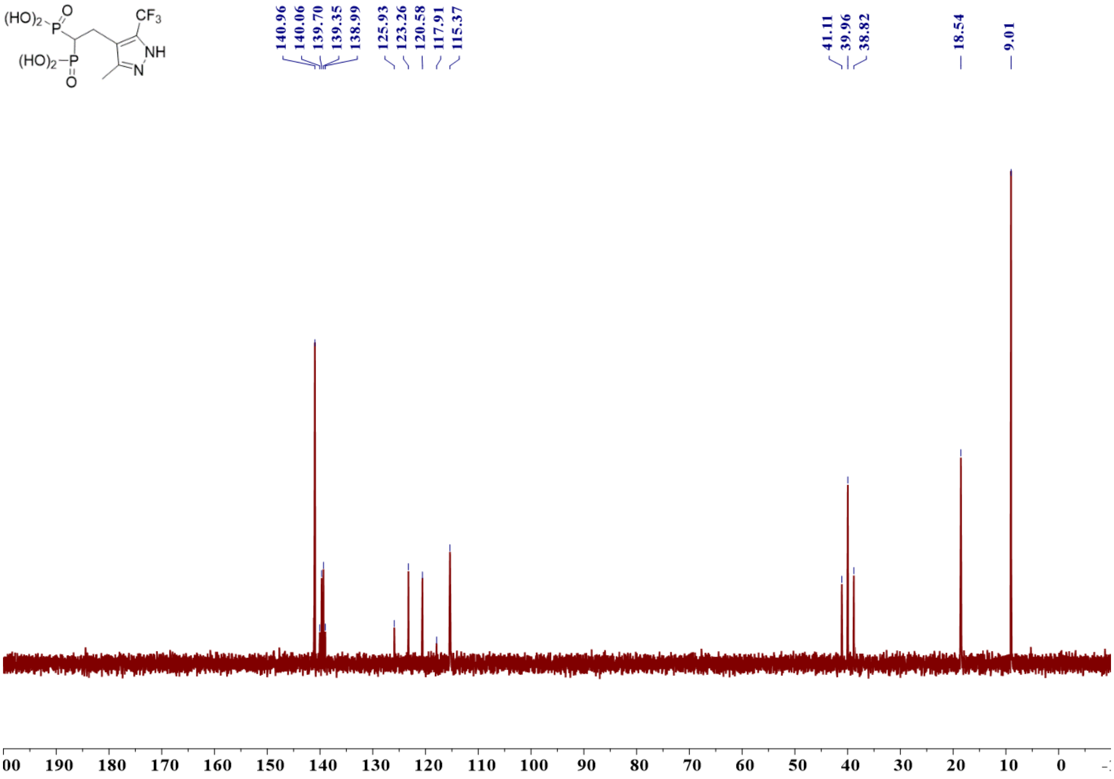

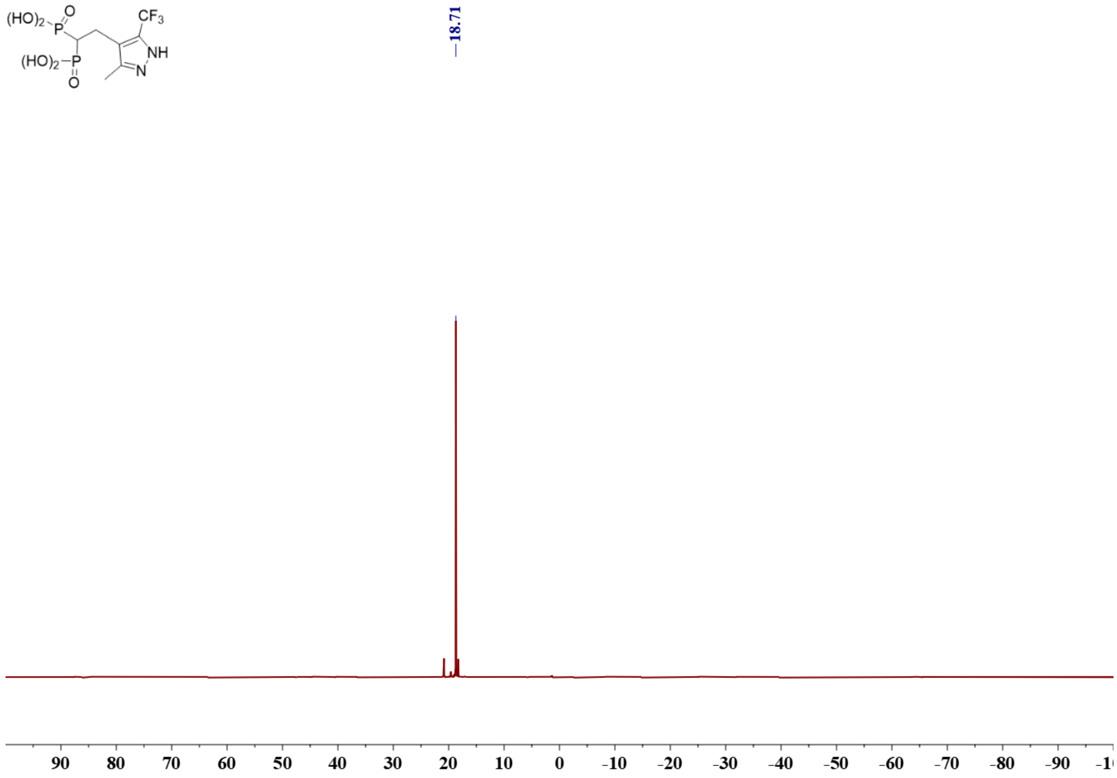
(HO)2
P
O
(HO)2
P
O
CF3
NH
N
18.71
90 80 70 60 50 40 30 20 10 0 -10 -20 -30 -40 -50 -60 -70 -80 -90

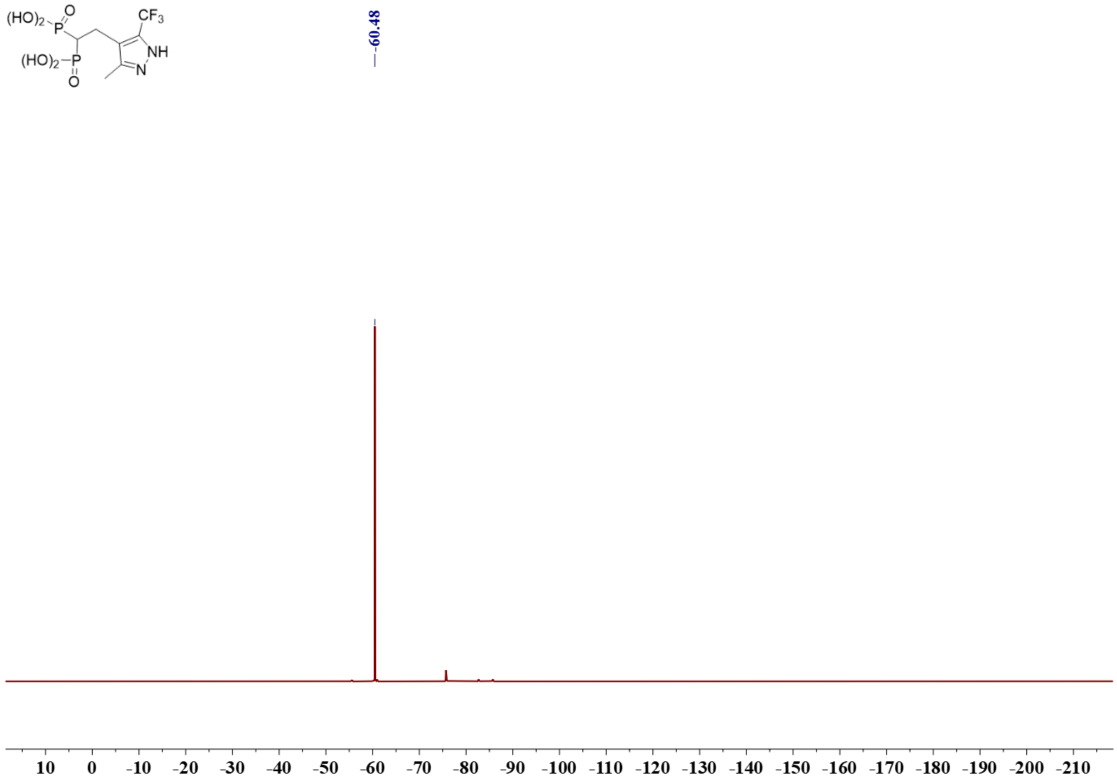
(HO)2
P
O
(HO)2
P
O
CF3
NH
N
-60.48
10 0 -10 -20 -30 -40 -50 -60 -70 -80 -90 -100 -110 -120 -130 -140 -150 -160 -170 -180 -190 -200 -210

(2-(3-hydroxy-5-methylisoxazol-4-yl)ethane-1,1-diyl)bis(phosphonic acid) (**13a**)

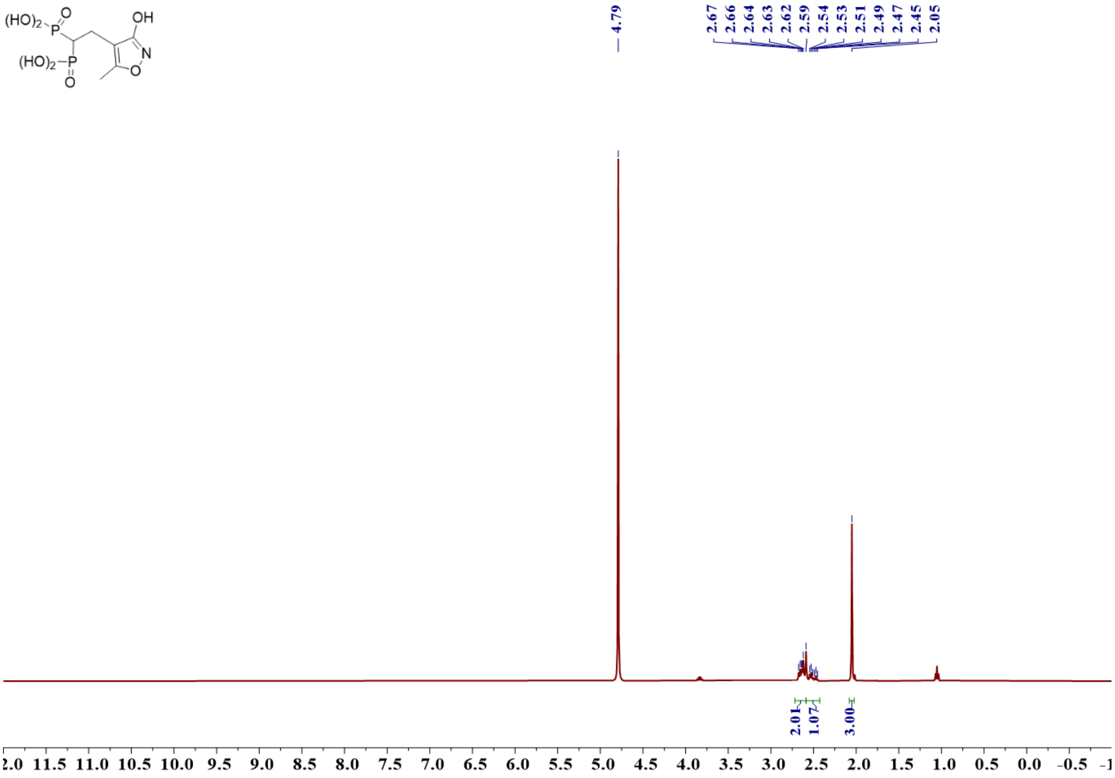

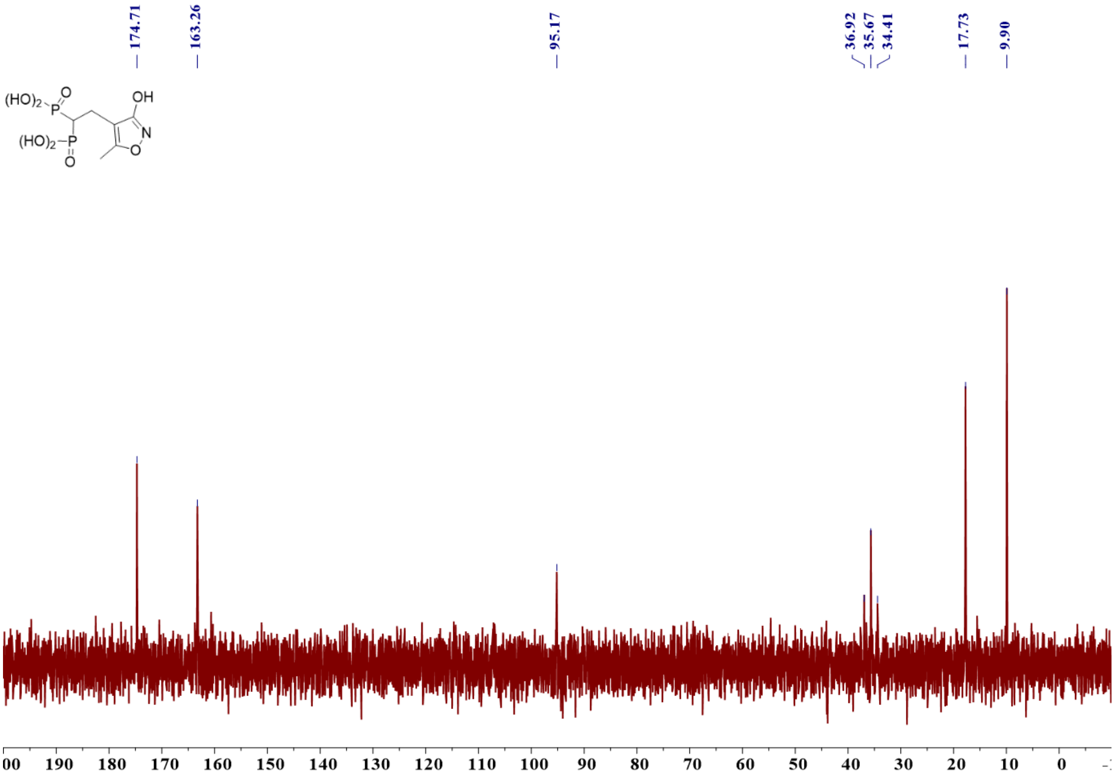

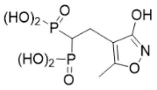
(HO)2
(HO)2
OH
N
O

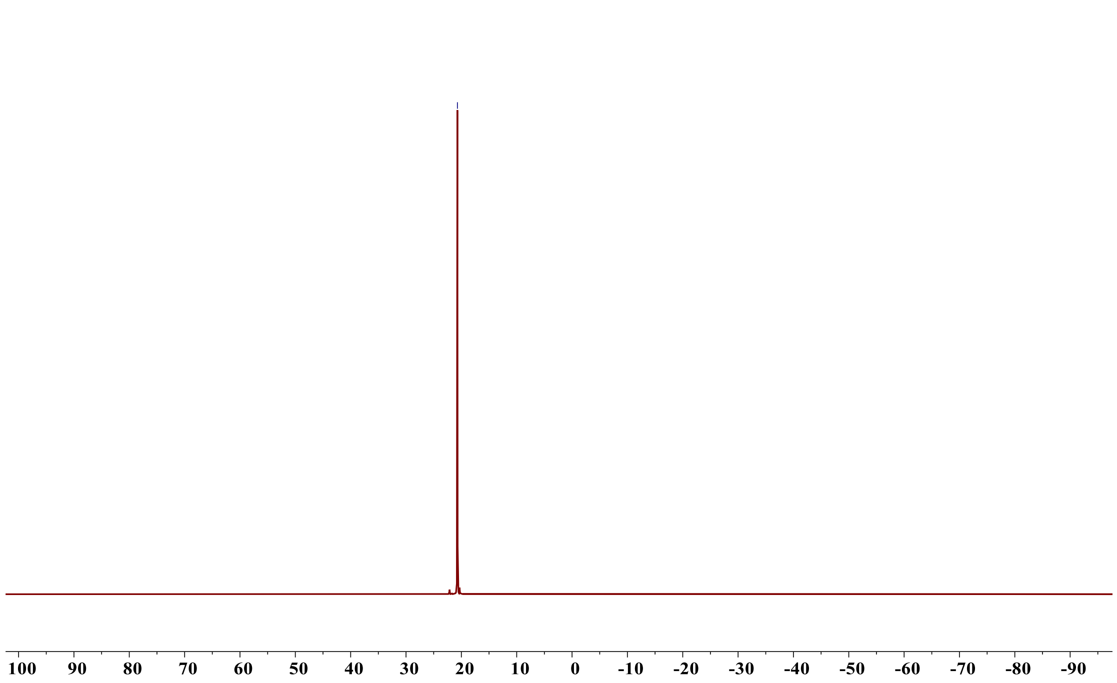
20.72
100 90 80 70 60 50 40 30 20 10 0 -10 -20 -30 -40 -50 -60 -70 -80 -90

(2-(5-ethyl-3-hydroxyisoxazol-4-yl)ethane-1,1-diyl)bis(phosphonic acid) (**13b**)

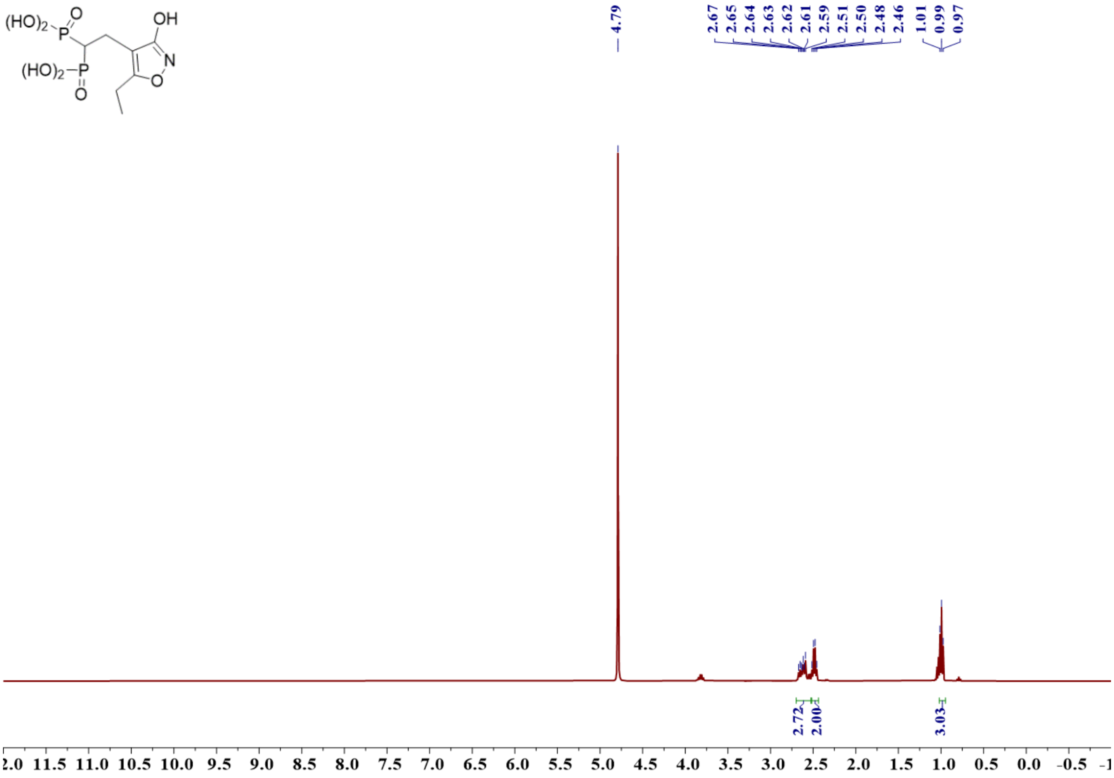

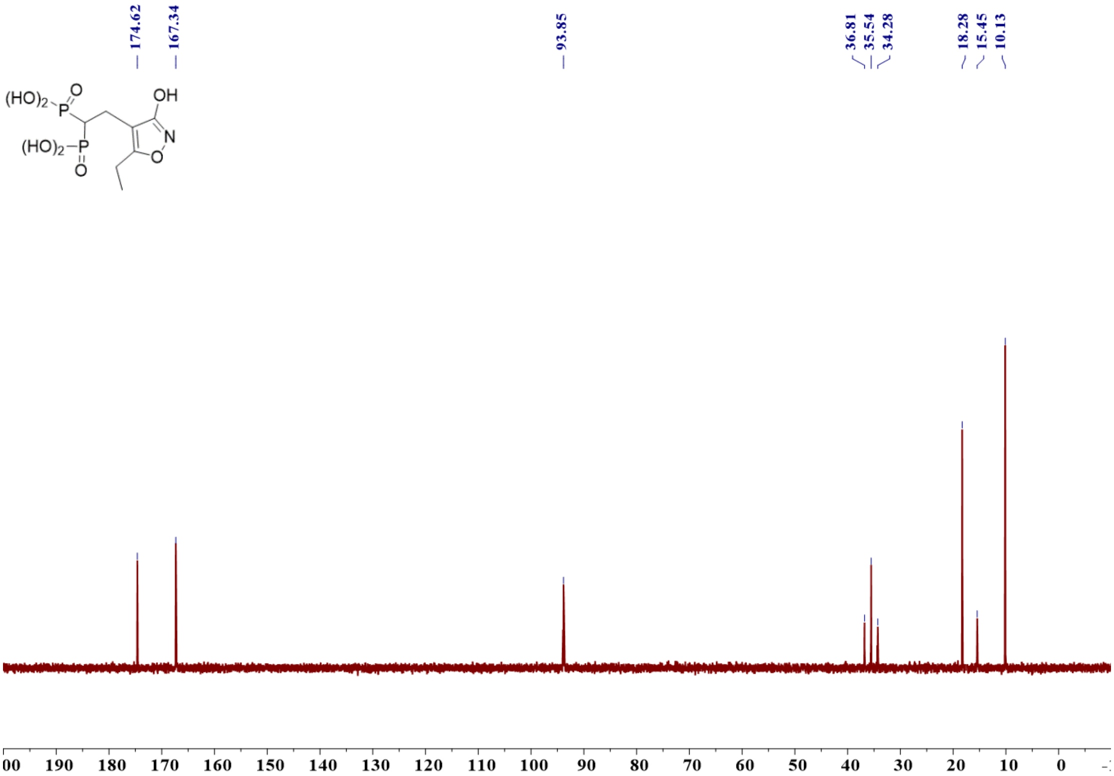

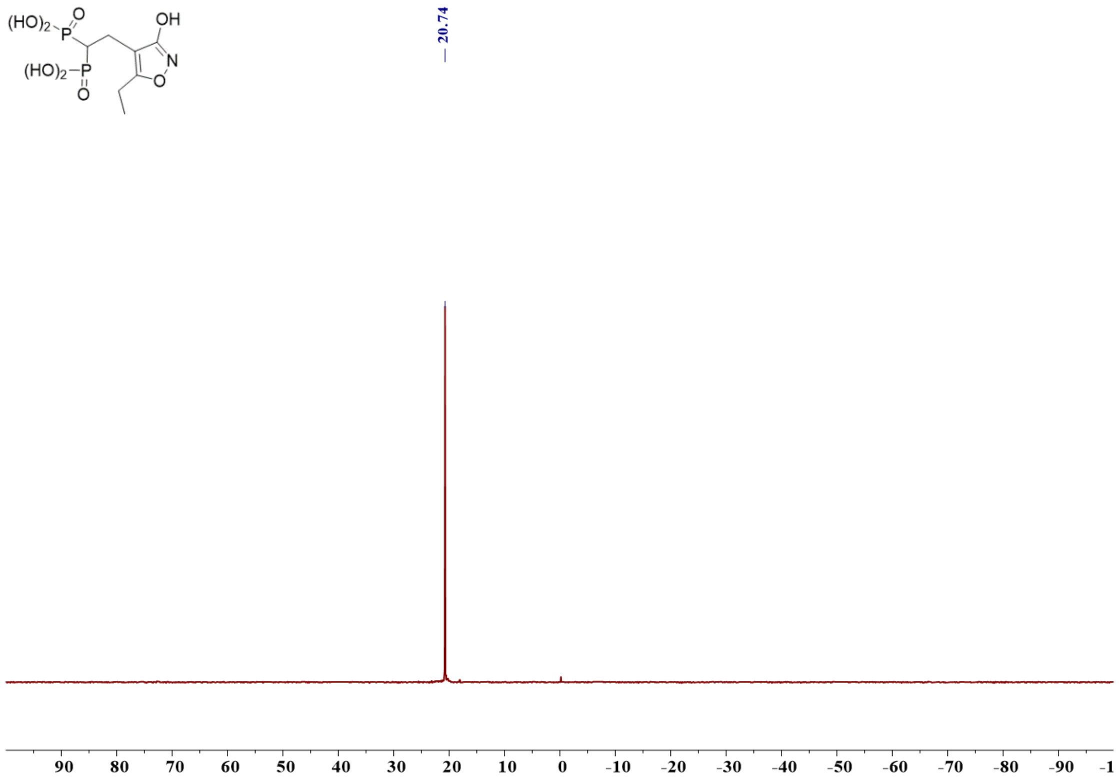
(HO)2
O
P
OH
N
O
(HO)2
P
O
20.74
90
80
70
60
50
40
30
20
10
0
-10
-20
-30
-40
-50
-60
-70
-80
-90
-1

(2-(3-hydroxy-5-propylisoxazol-4-yl)ethane-1,1-diyl)bis(phosphonic acid) (**13c**)

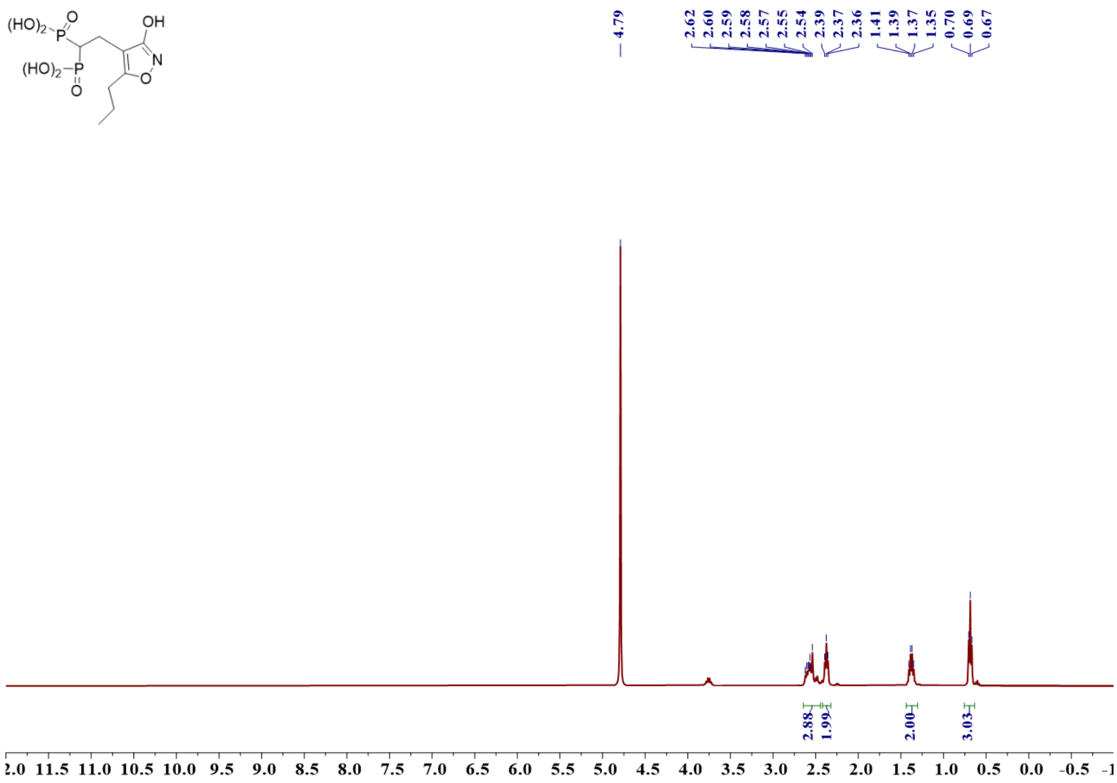

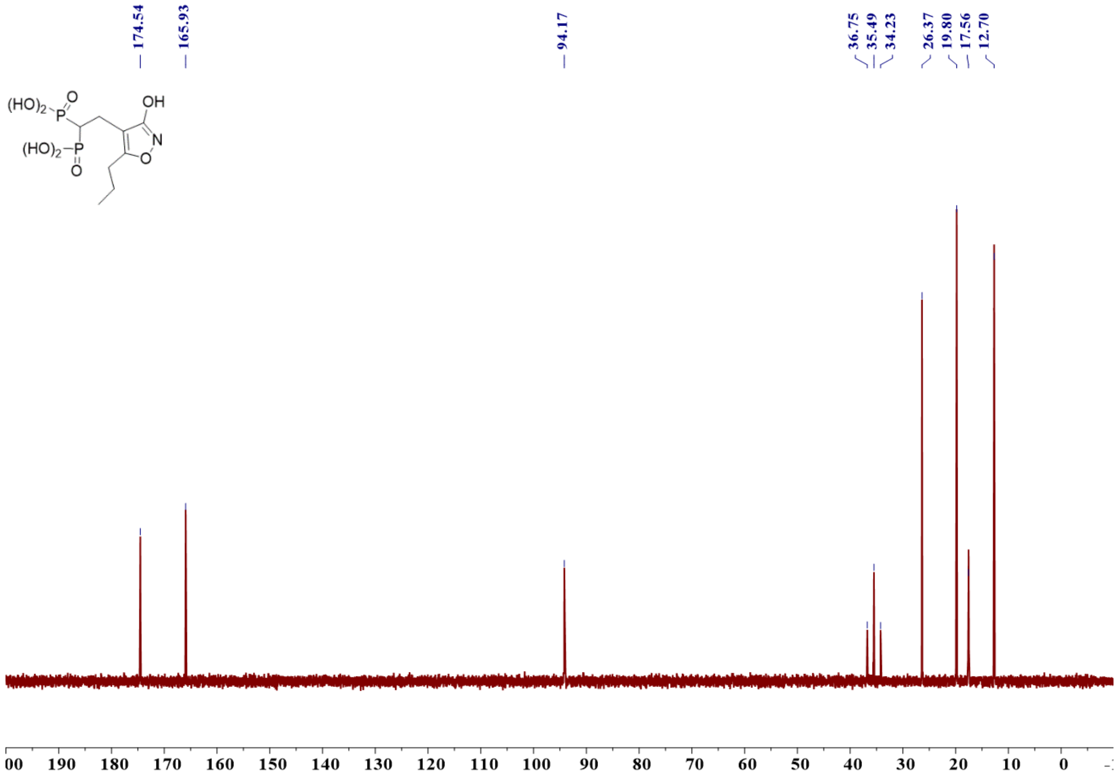

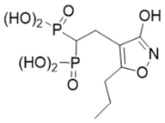

(HO)2
O
P
(HO)2
P
O
OH
N
O

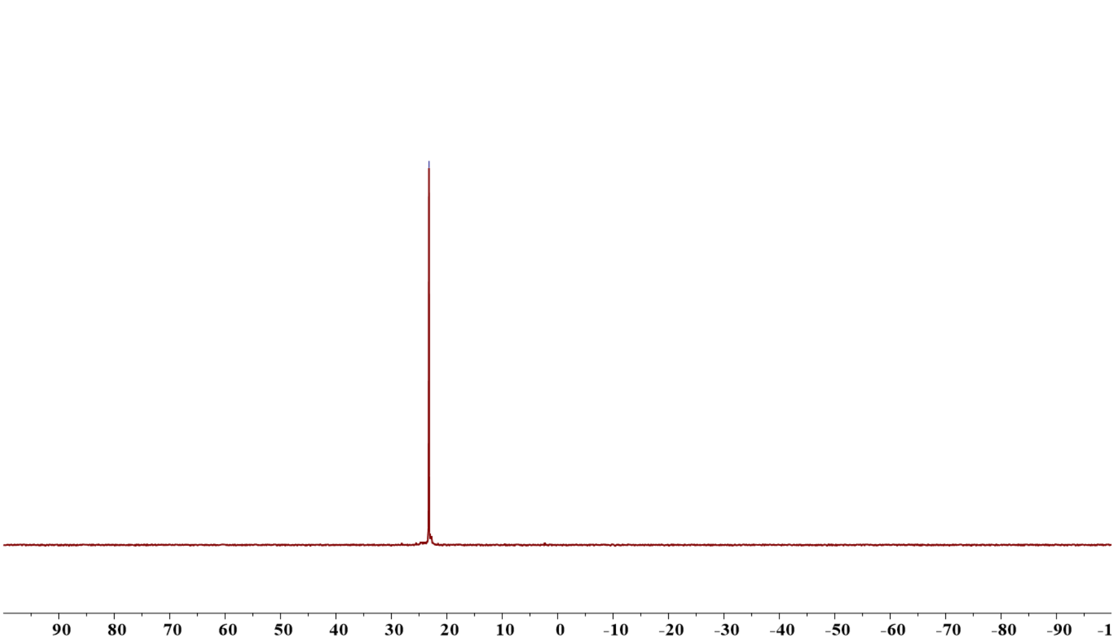

23.22
90
80
70
60
50
40
30
20
10
0
-10
-20
-30
-40
-50
-60
-70
-80
-90
-1

(2-(3-hydroxy-5-isopropylisoxazol-4-yl)ethane-1,1-diyl)bis(phosphonic acid) (**13d**)

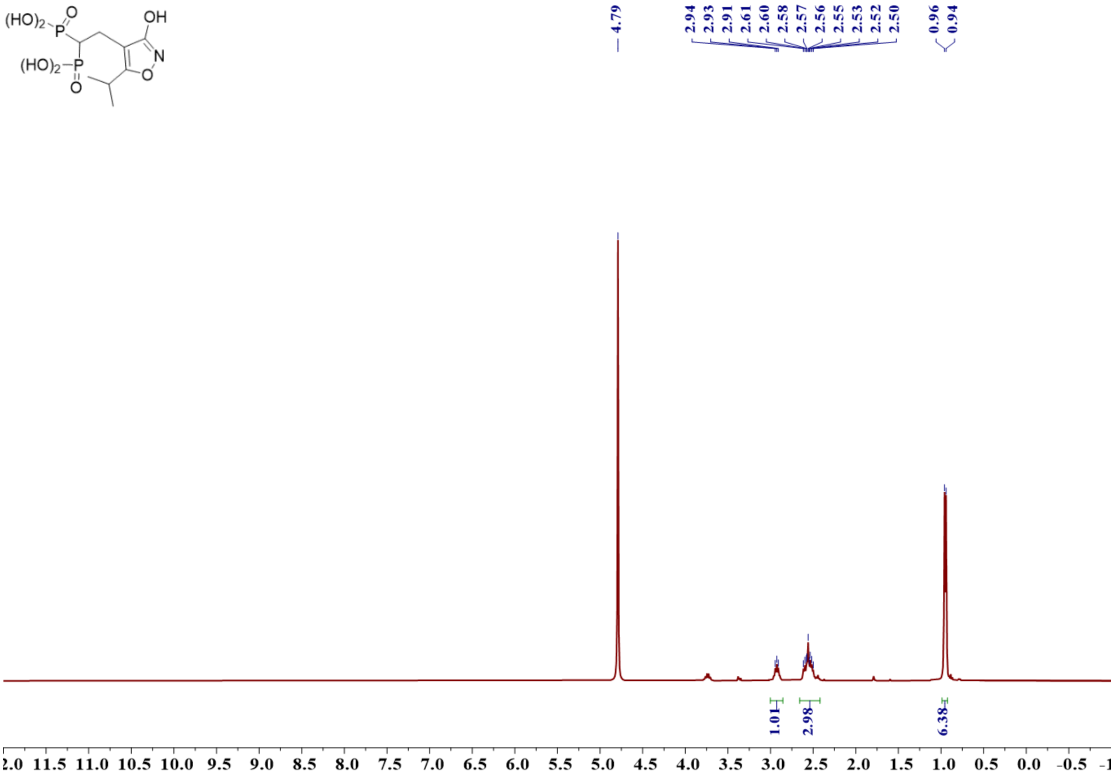

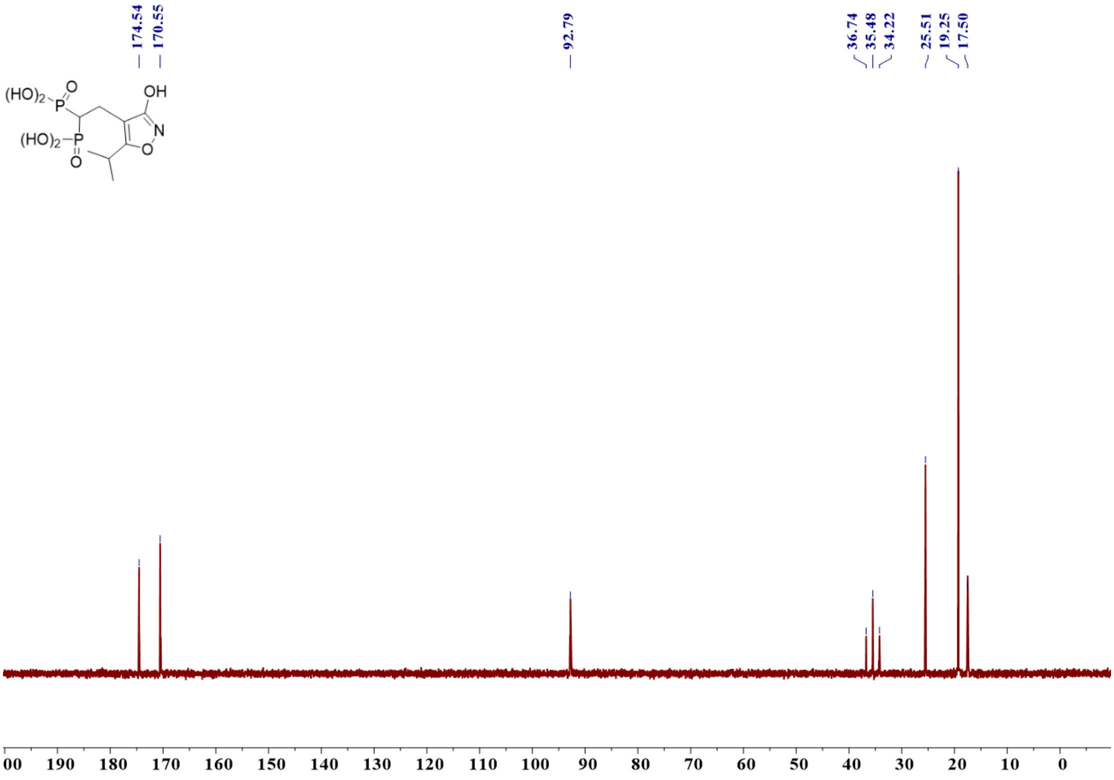

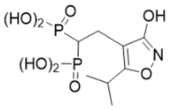

(HO)2
O
P
OH
(HO)2
P
N
O
O

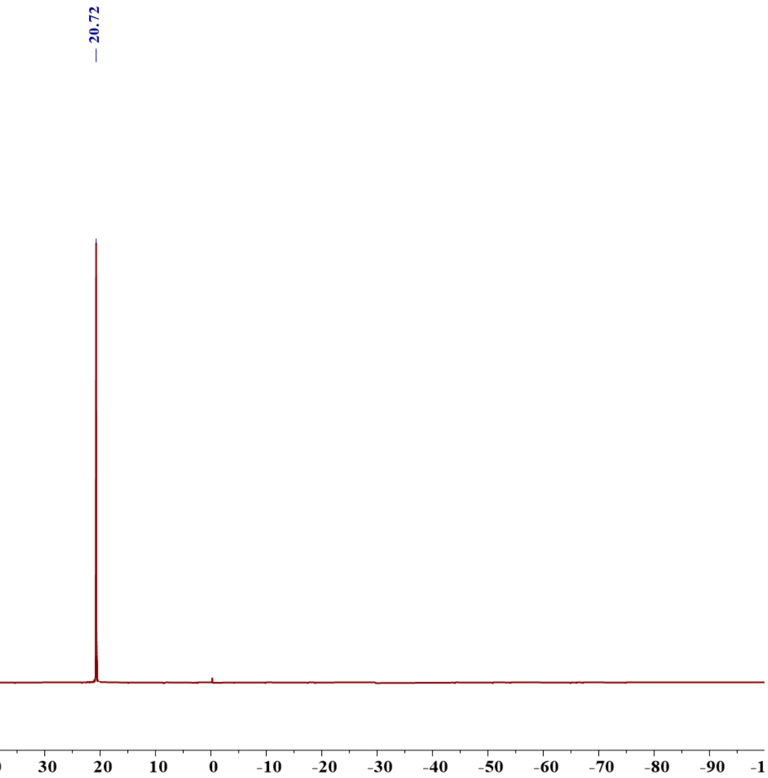

20.72
90
80
70
60
50
40
30
20
10
0
-10
-20
-30
-40
-50
-60
-70
-80
-90
-1

(2-(3-hydroxy-5-(trifluoromethyl)isoxazol-4-yl)ethane-1,1-diyl)bis(phosphonic acid) (**13e**)

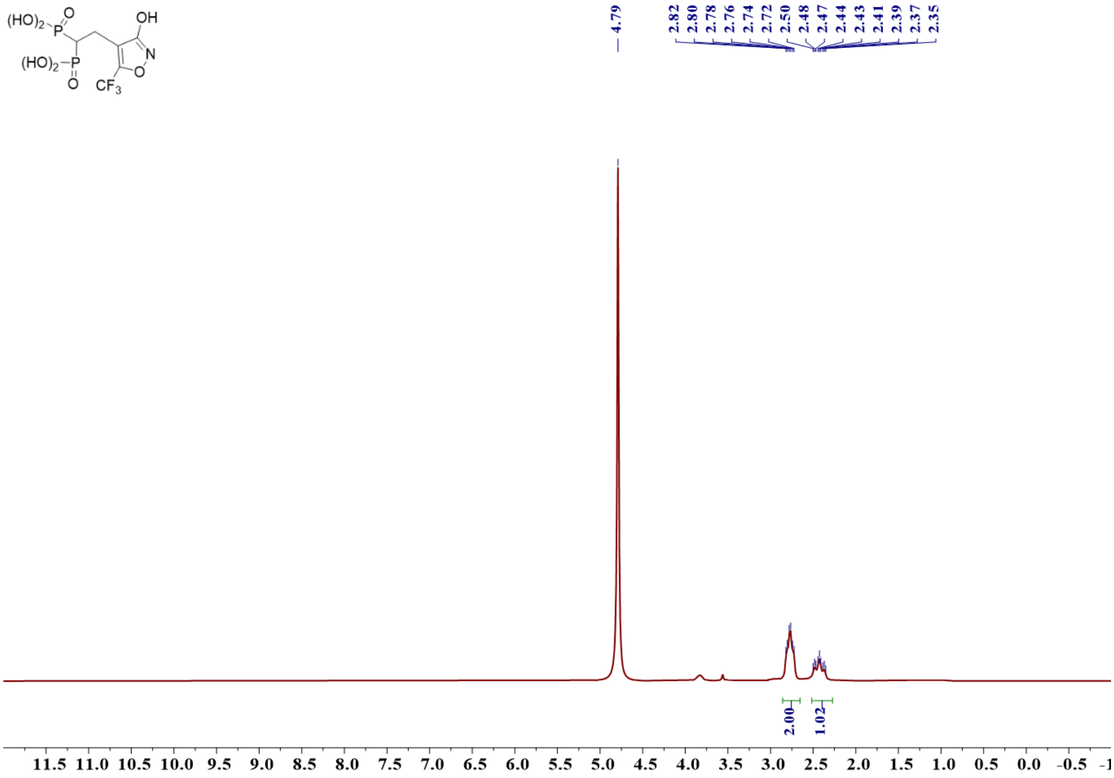

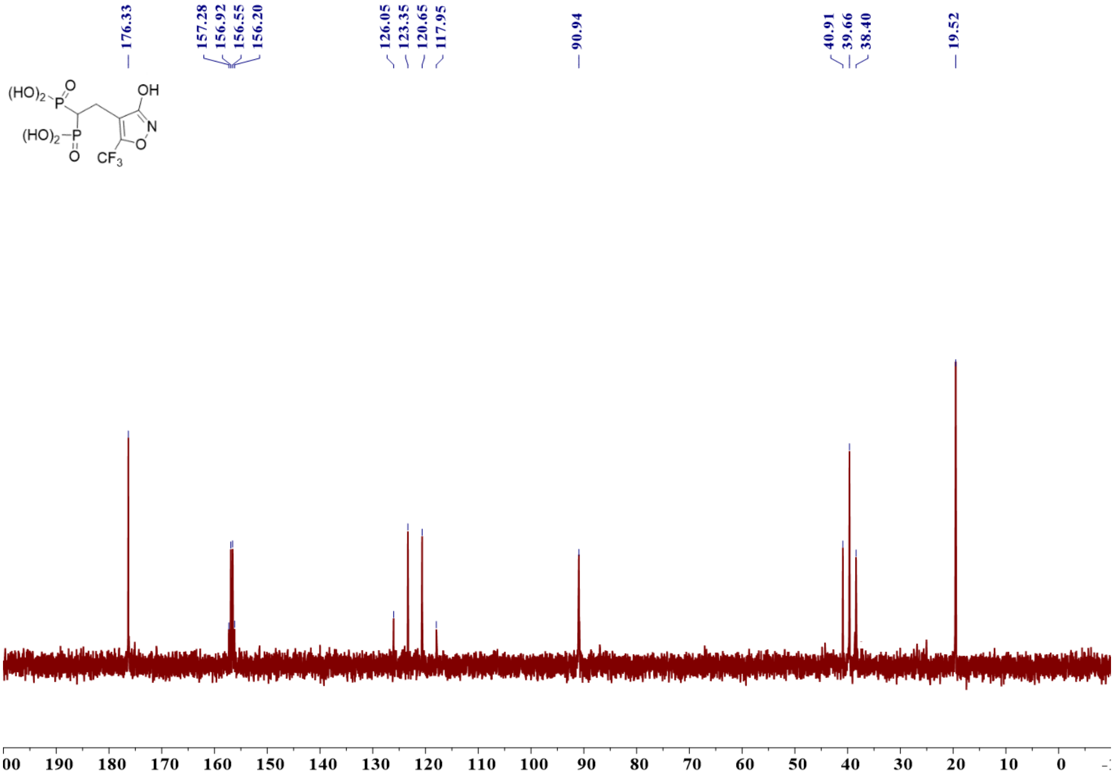

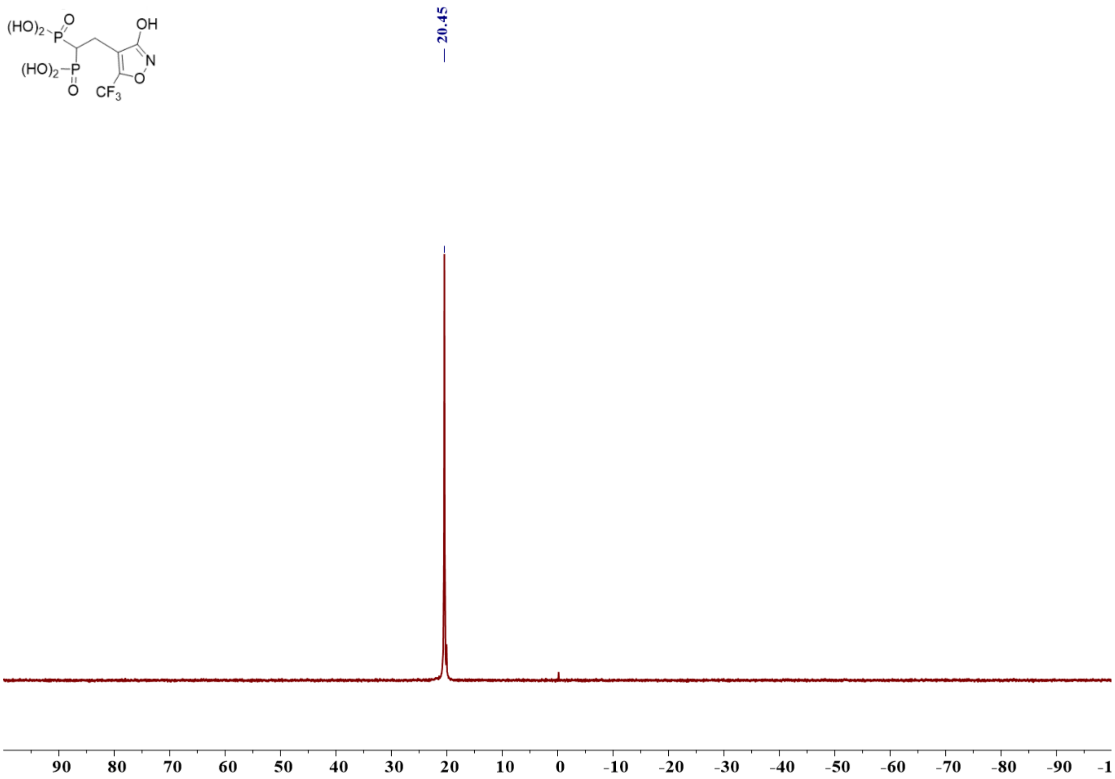
(HO)2
O
P
(HO)2
P
O
OH
N
O
CF3
20.45
90
80
70
60
50
40
30
20
10
0
-10
-20
-30
-40
-50
-60
-70
-80
-90

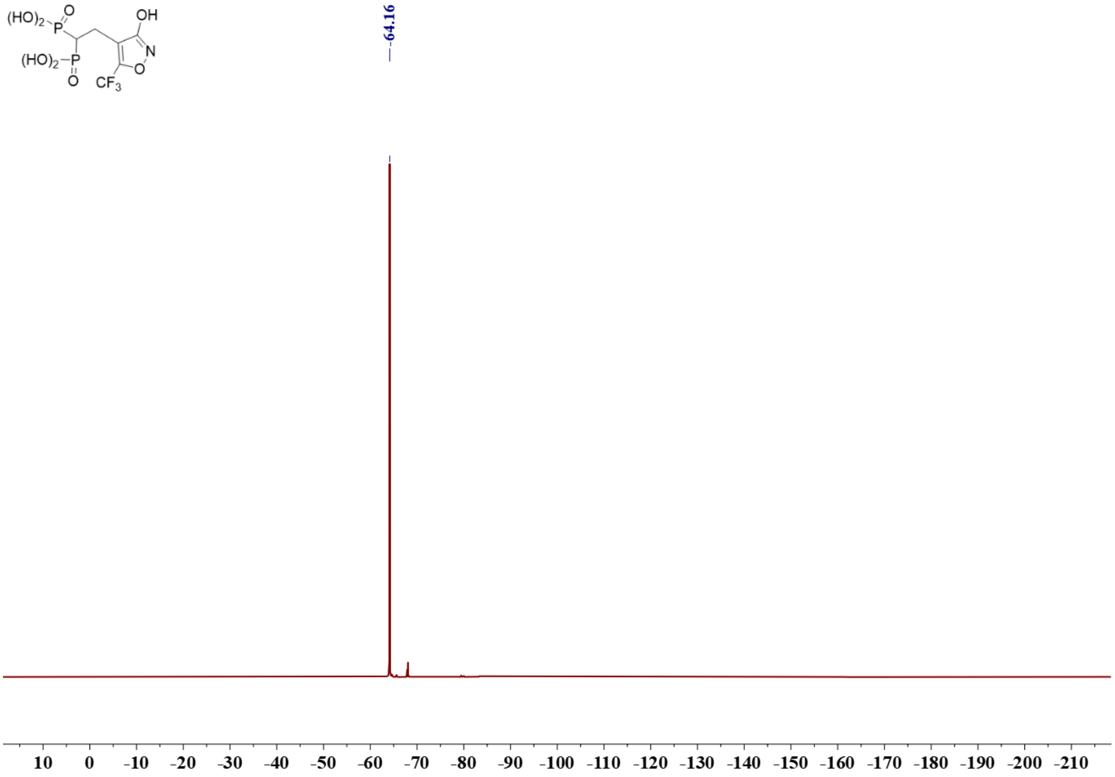
(HO)2
O
P
(HO)2
P
O
OH
N
O
CF3
-64.16
10
0
-10
-20
-30
-40
-50
-60
-70
-80
-90
-100
-110
-120
-130
-140
-150
-160
-170
-180
-190
-200
-210

(2-(5-(1,1-difluoroethyl)-3-hydroxyisoxazol-4-yl)ethane-1,1-diyl)bis(phosphonic acid) (**13f**)

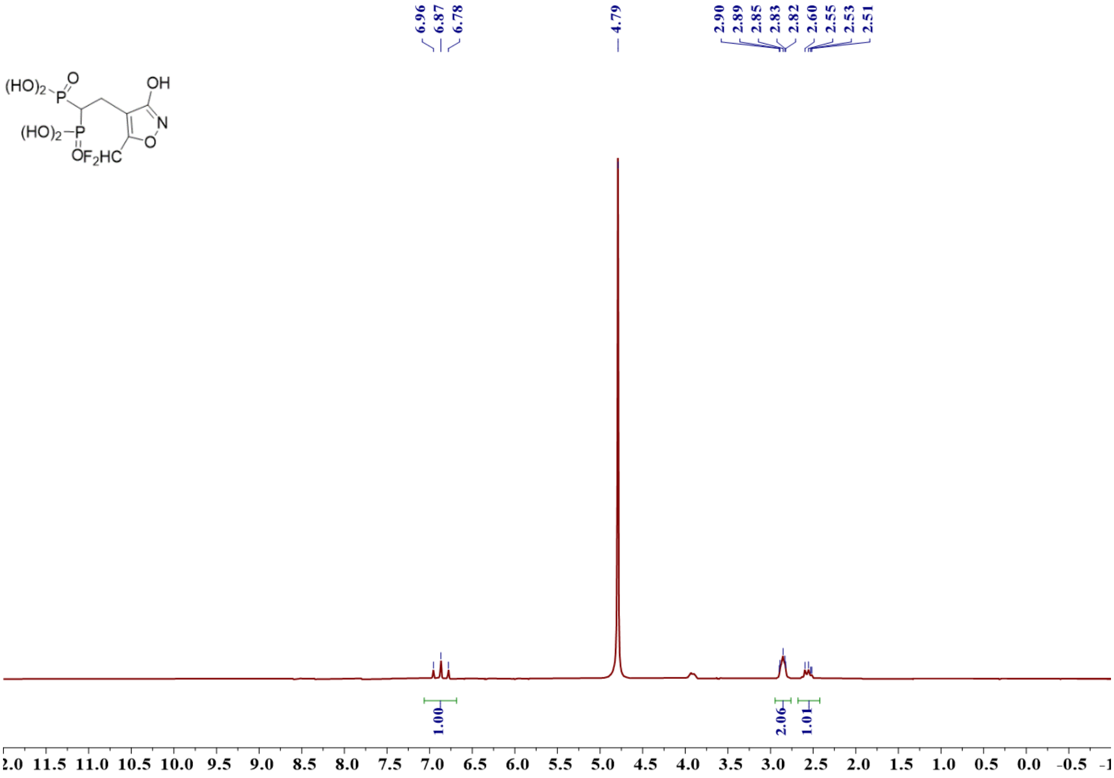

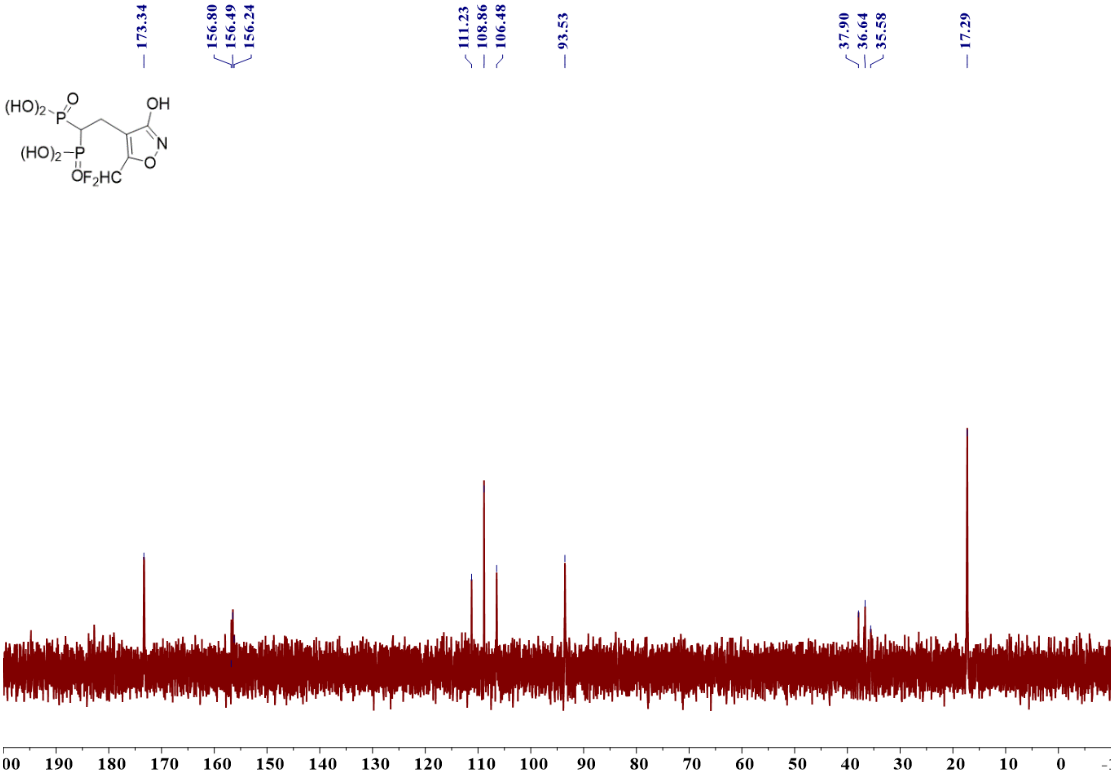

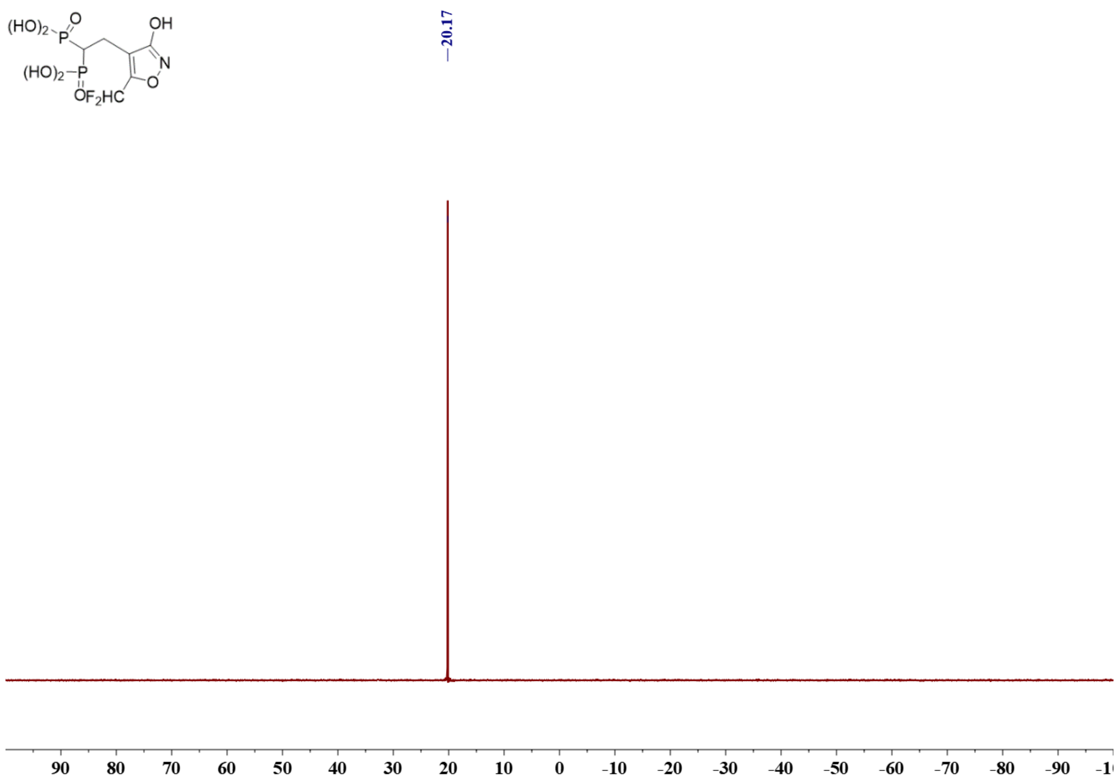
(HO)2 P O
(HO)2 P O
OH
N
O
F2HC
20.17
90 80 70 60 50 40 30 20 10 0 -10 -20 -30 -40 -50 -60 -70 -80 -90

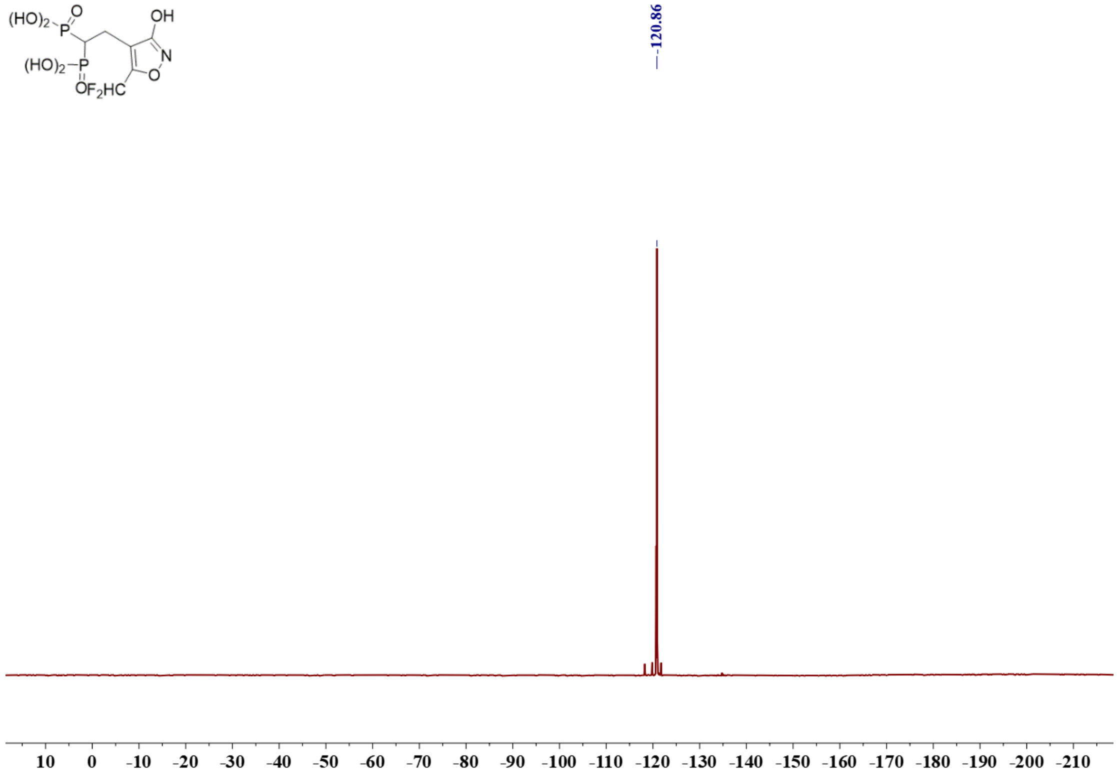
(HO)2 P O
(HO)2 P O
OH
N
O
F2HC
-120.86
10 0 -10 -20 -30 -40 -50 -60 -70 -80 -90 -100 -110 -120 -130 -140 -150 -160 -170 -180 -190 -200 -210

(2-(3,5-dimethylisoxazol-4-yl)ethane-1,1-diyl)bis(phosphonic acid) (**13g**)

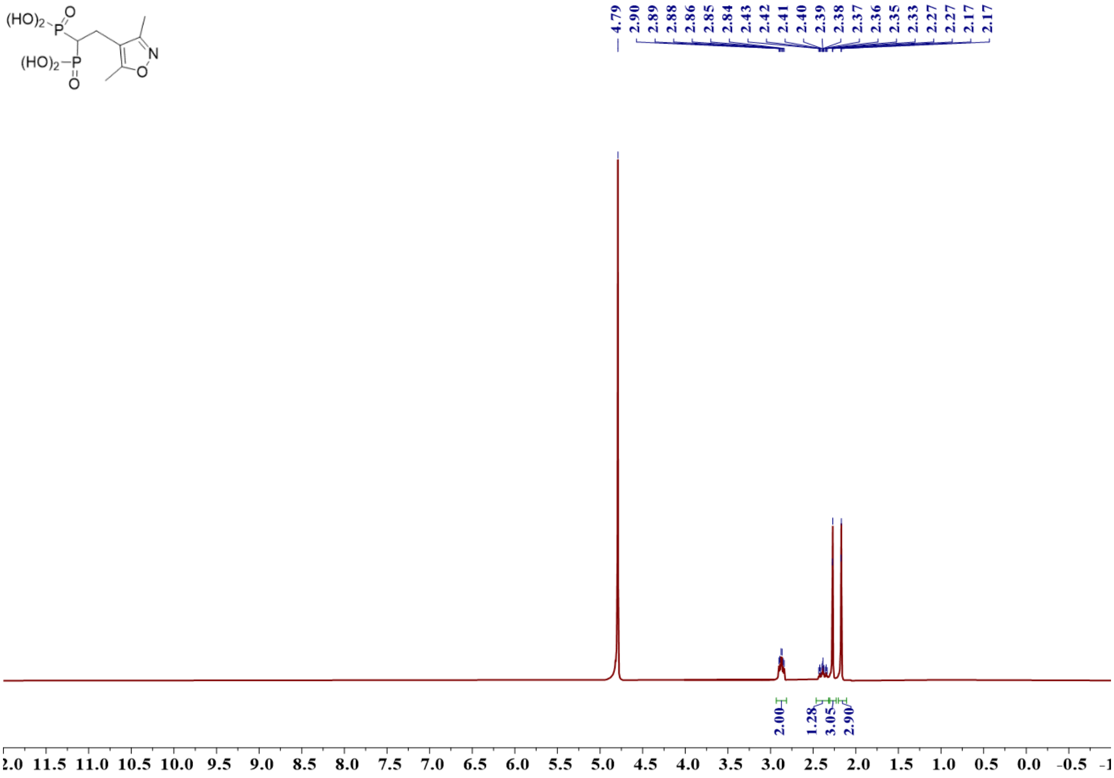

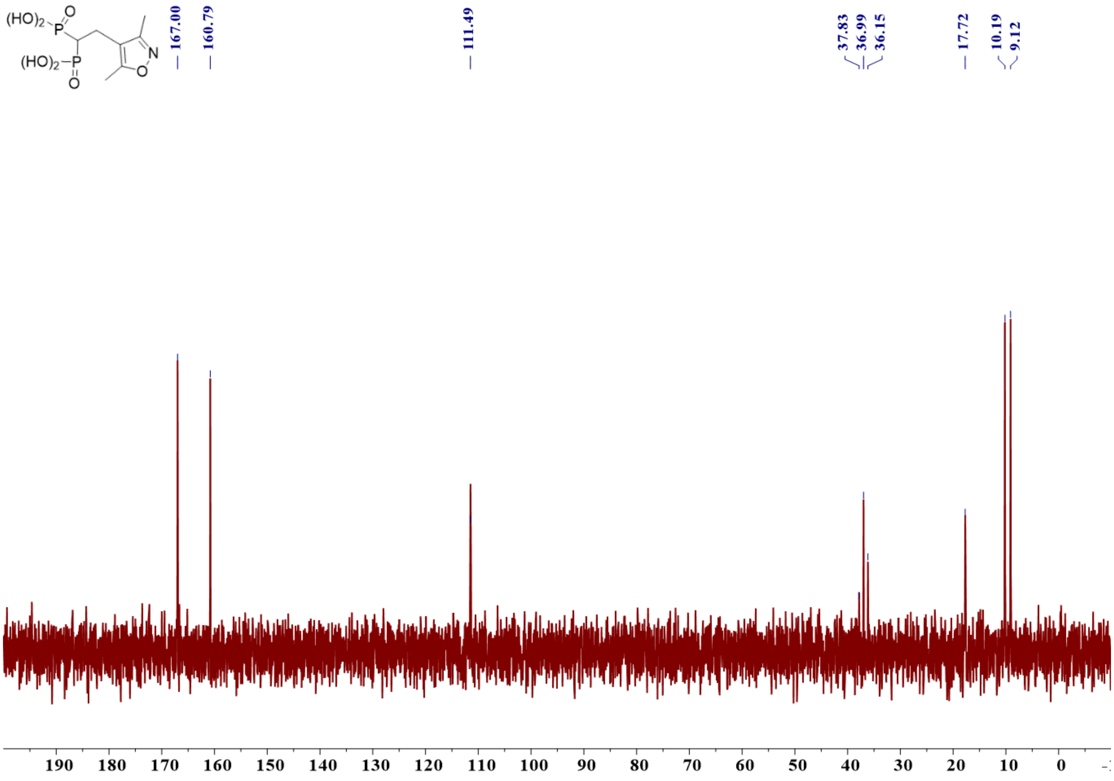

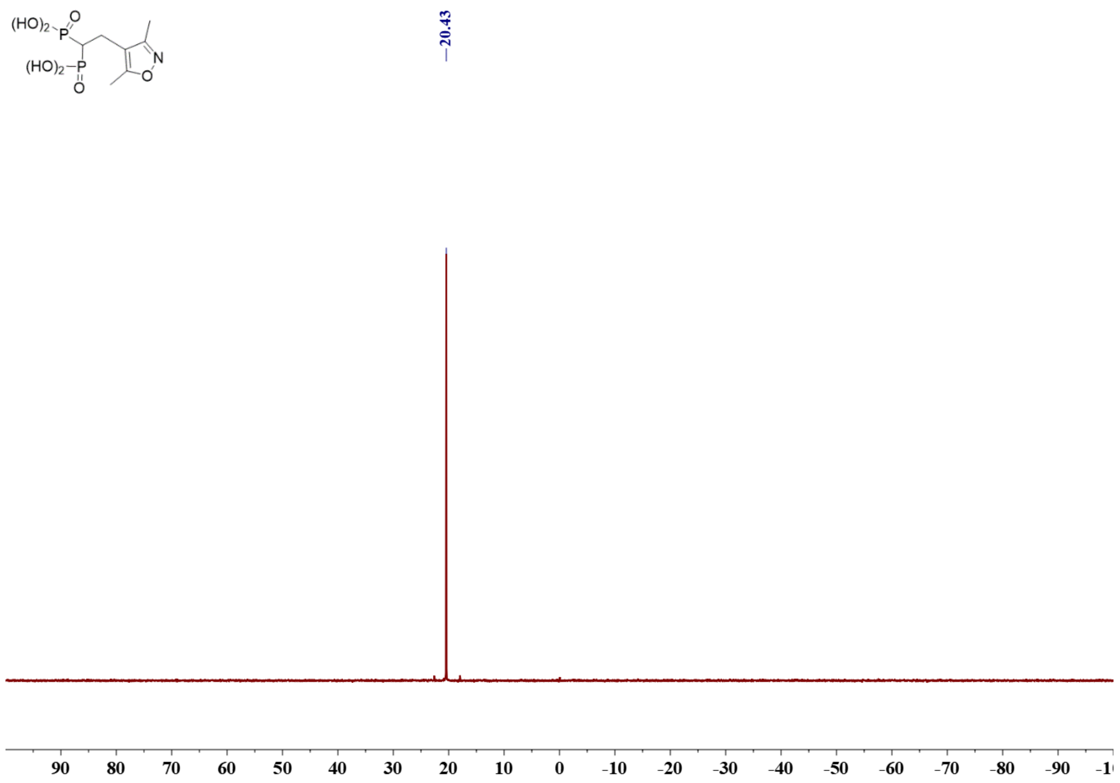
(HO)2
(HO)2
P
P
O
O
N
O
20.43
90 80 70 60 50 40 30 20 10 0 -10 -20 -30 -40 -50 -60 -70 -80 -90

(2-(3,5-diethylisoxazol-4-yl)ethane-1,1-diyl)bis(phosphonic acid) (**13h**)

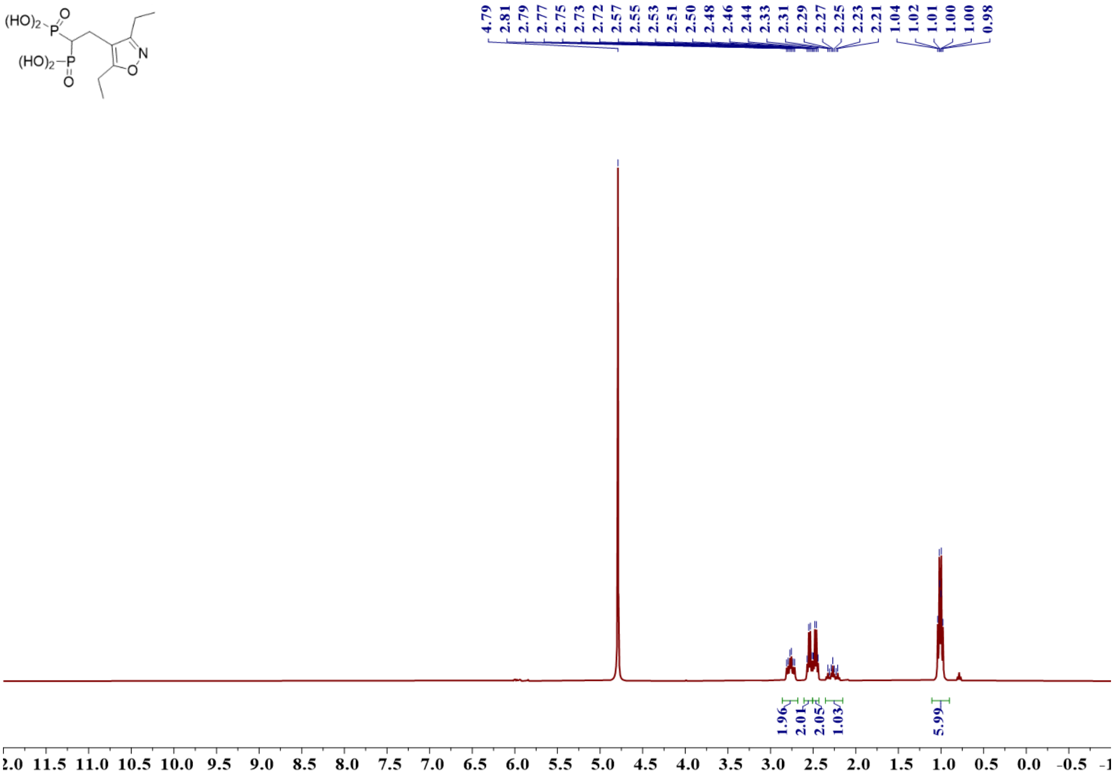

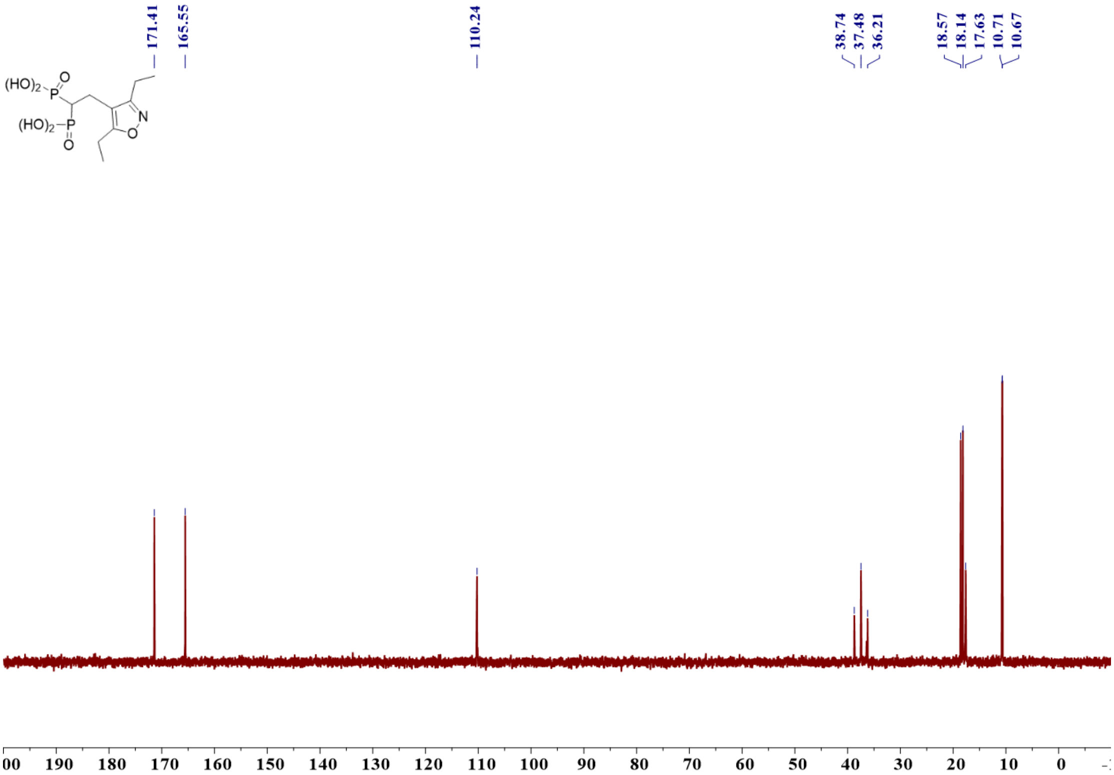

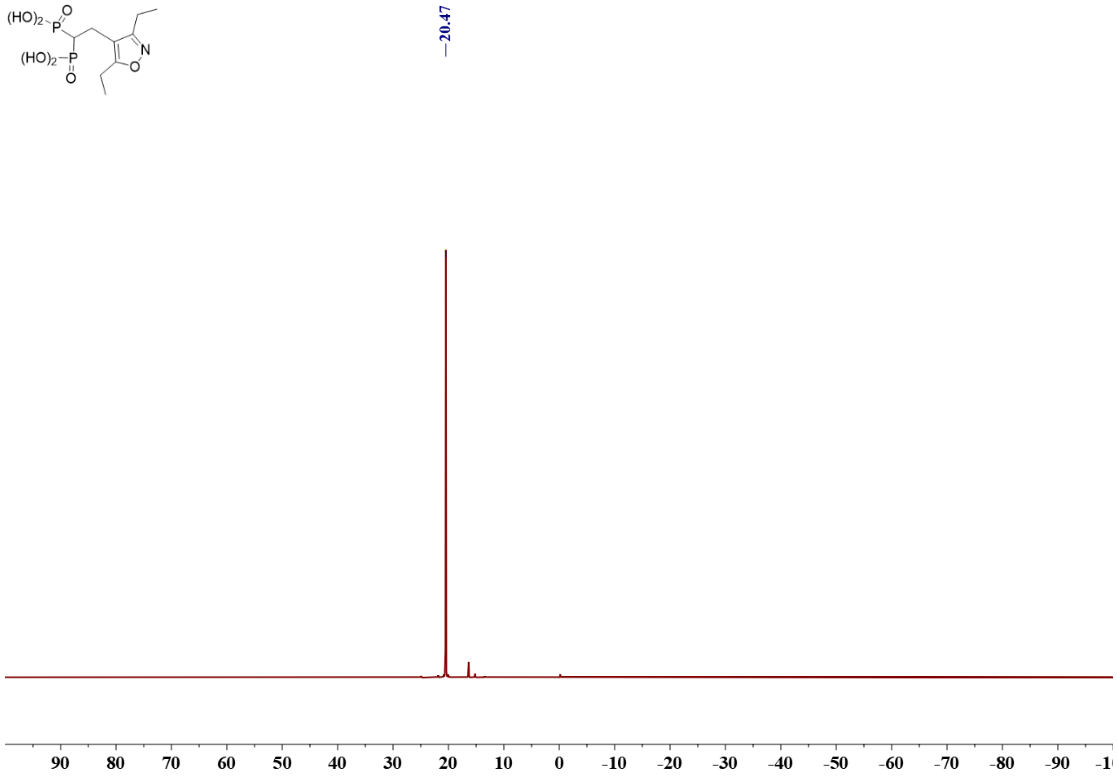
(HO)2
P
O
(HO)2
P
O
N
O
20.47
90
80
70
60
50
40
30
20
10
0
-10
-20
-30
-40
-50
-60
-70
-80
-90

(2-(5-methyl-3-(trifluoromethyl)isoxazol-4-yl)ethane-1,1-diyl)bis(phosphonic acid) (**13i**)

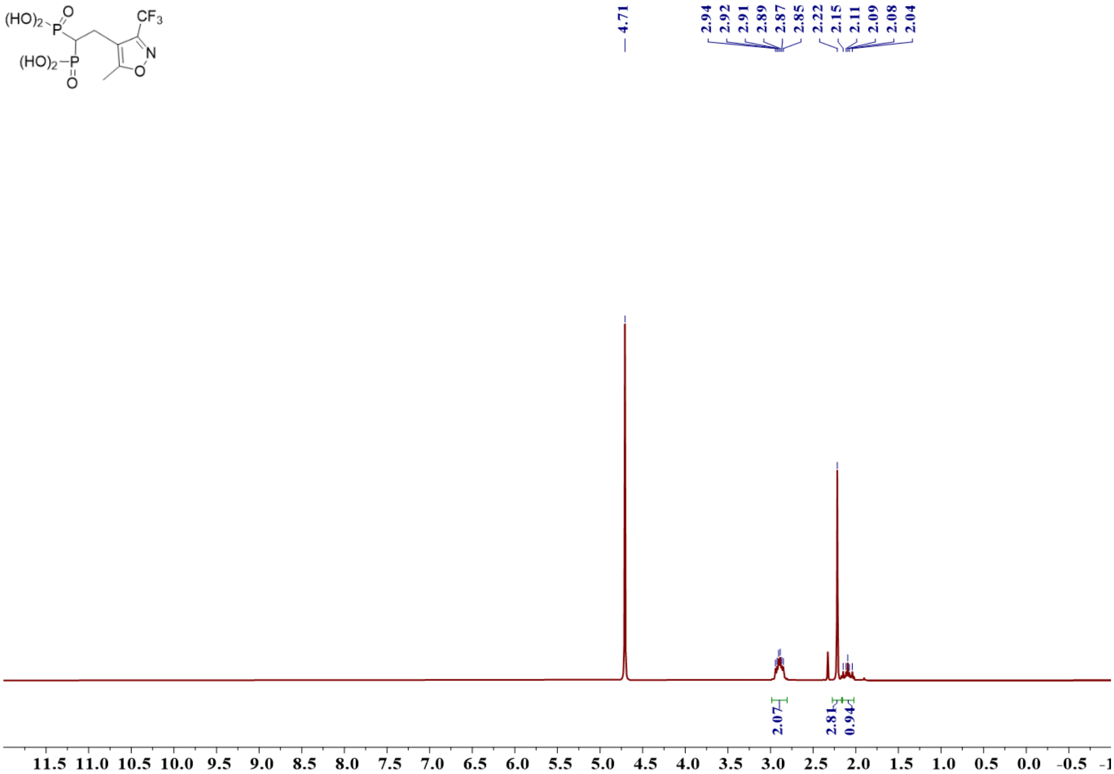

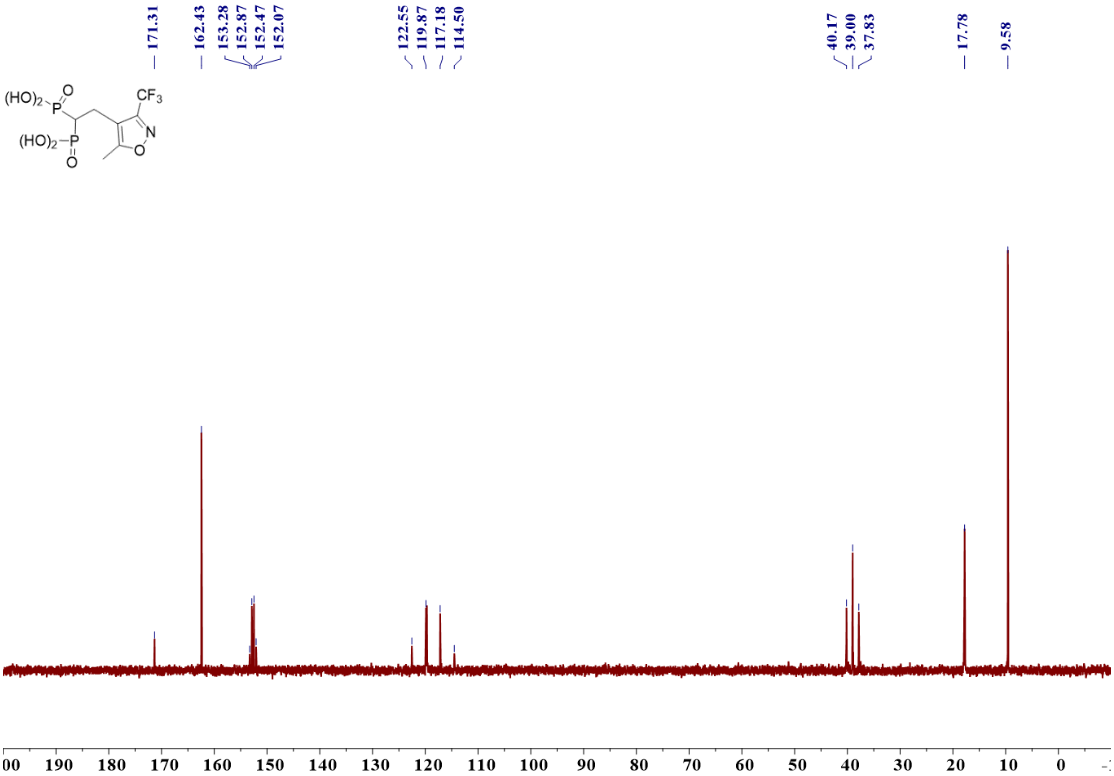

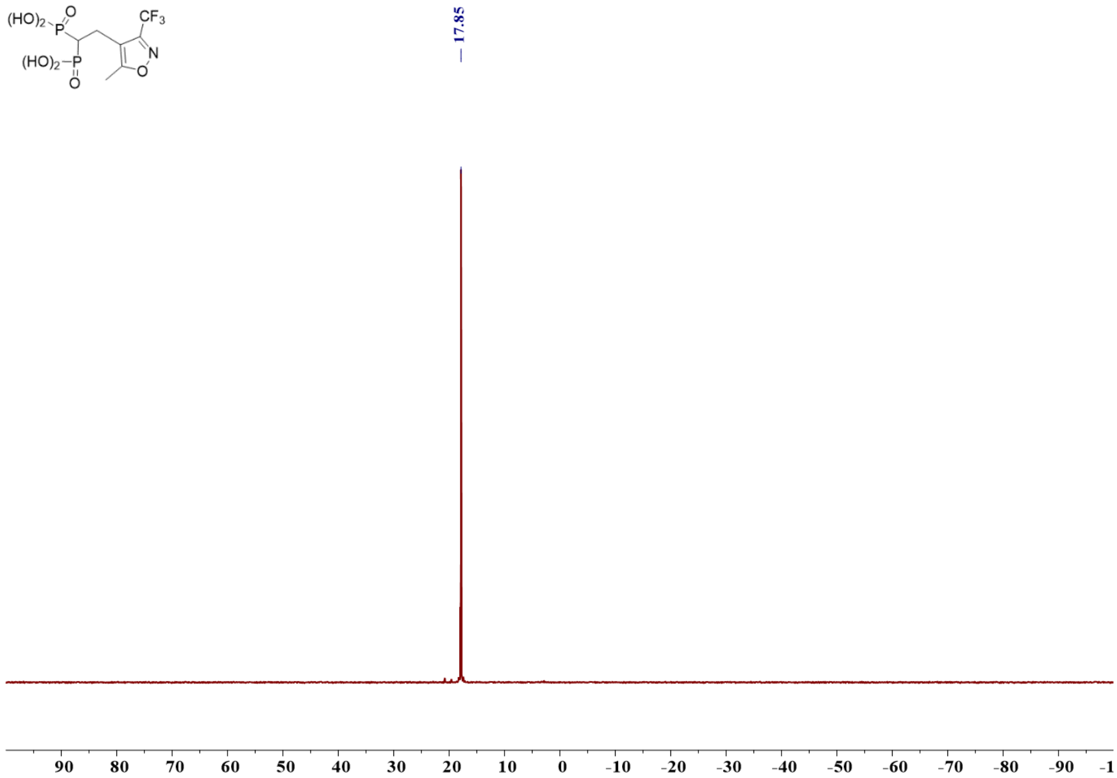
(HO)2
P
O
(HO)2
P
O
CF3
N
O
17.85
90
80
70
60
50
40
30
20
10
0
-10
-20
-30
-40
-50
-60
-70
-80
-90

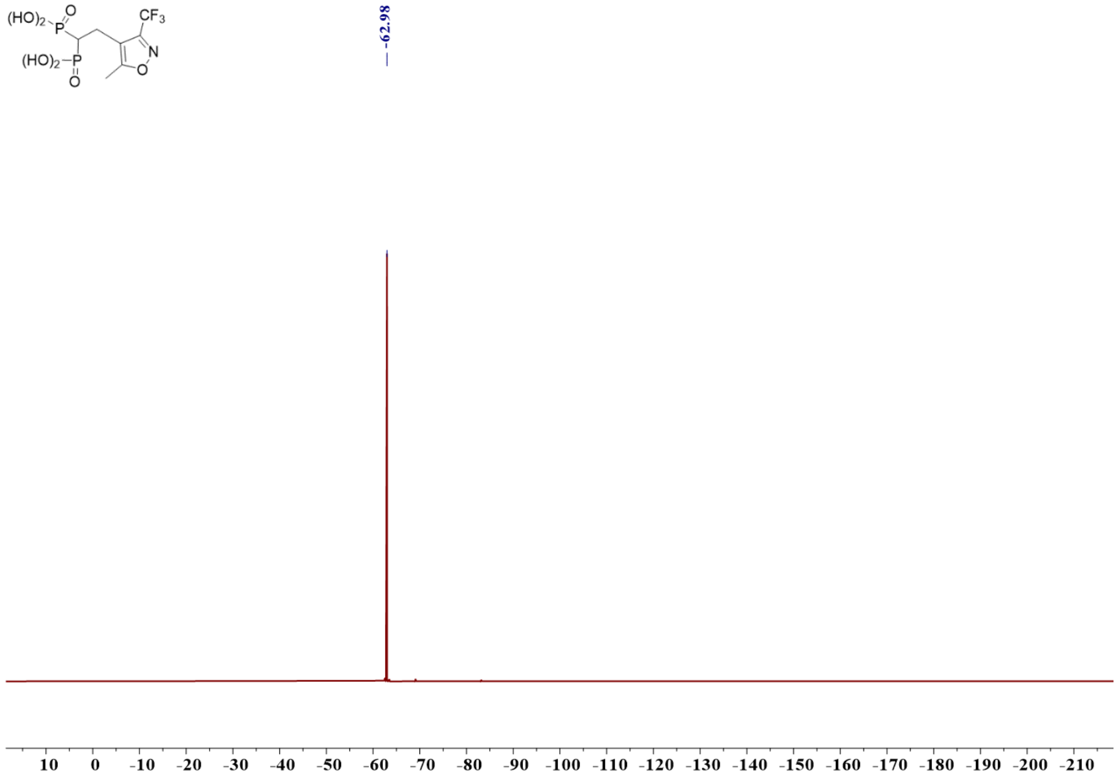
(HO)2
P
O
(HO)2
P
O
CF3
N
O
-62.98
10
0
-10
-20
-30
-40
-50
-60
-70
-80
-90
-100
-110
-120
-130
-140
-150
-160
-170
-180
-190
-200
-210

(3-(4,4-dimethyl-3-oxoisoxazolidin-2-yl)propyl)phosphonic acid (**19**)

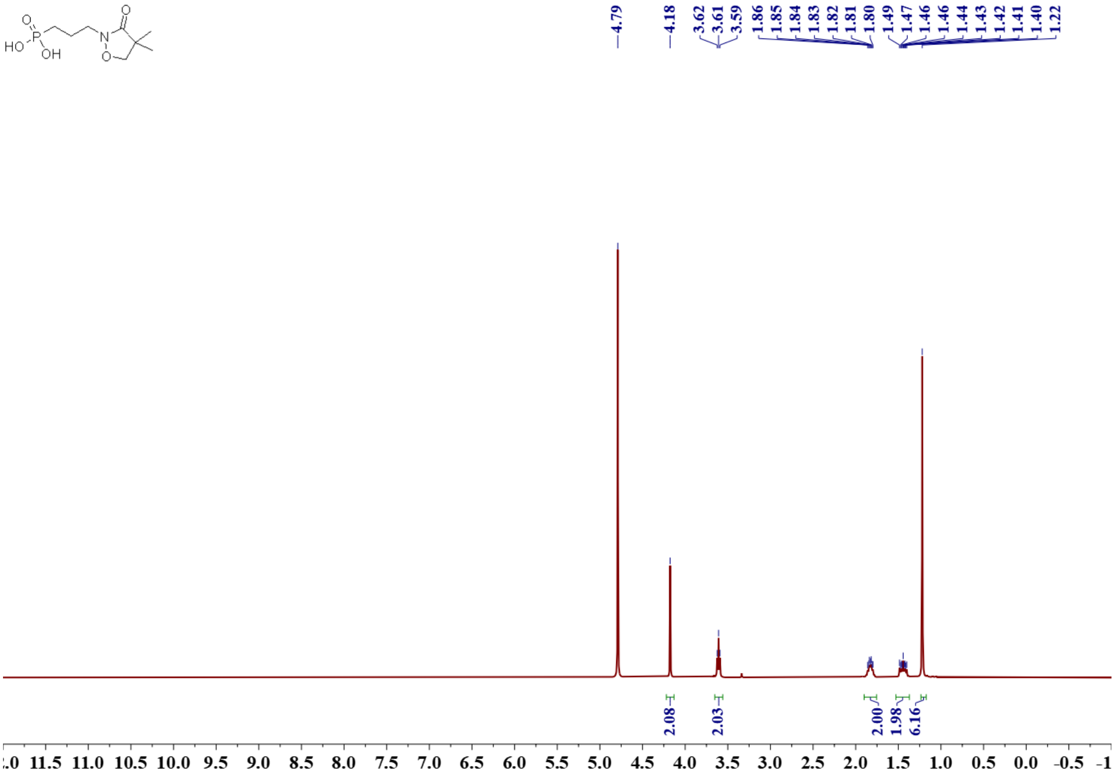

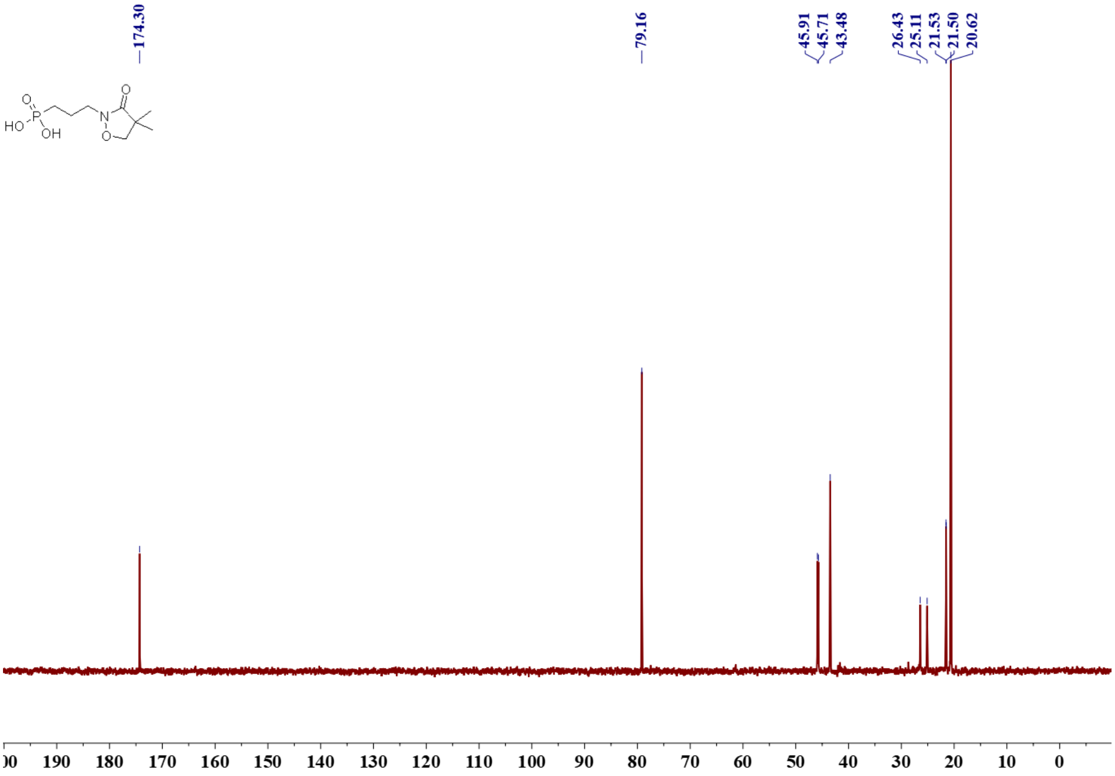

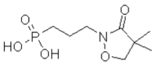

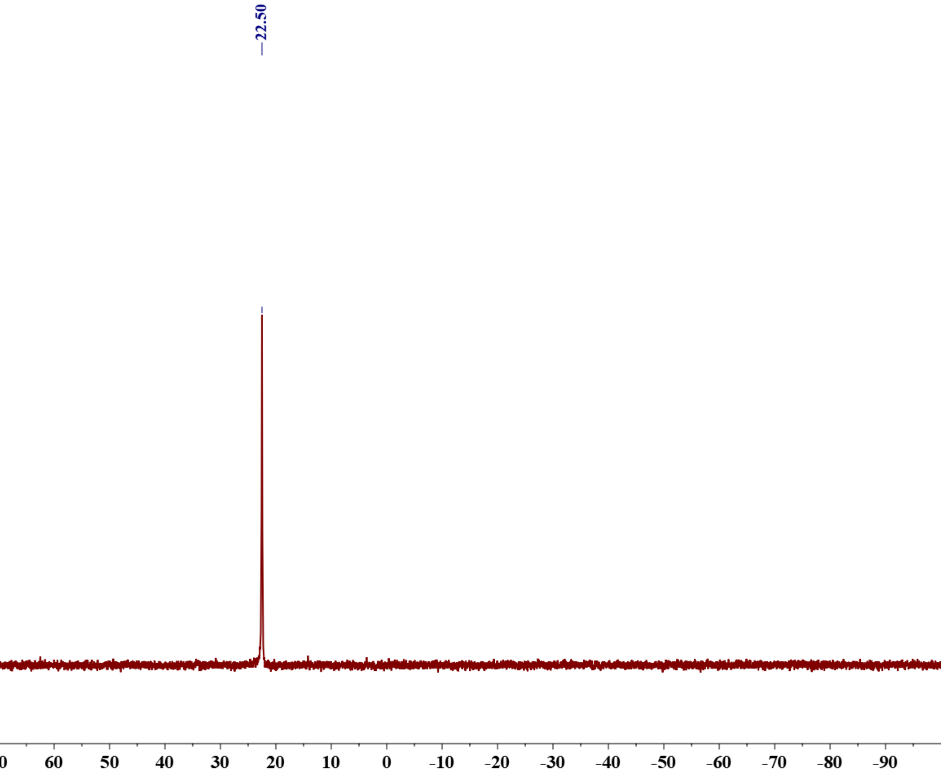

22.50
90
80
70
60
50
40
30
20
10
0
-10
-20
-30
-40
-50
-60
-70
-80
-90

(4-(4,4-dimethyl-3-oxoisoxazolidin-2-yl)butane-1,1-diyl)bis(phosphonic acid) (**20**)

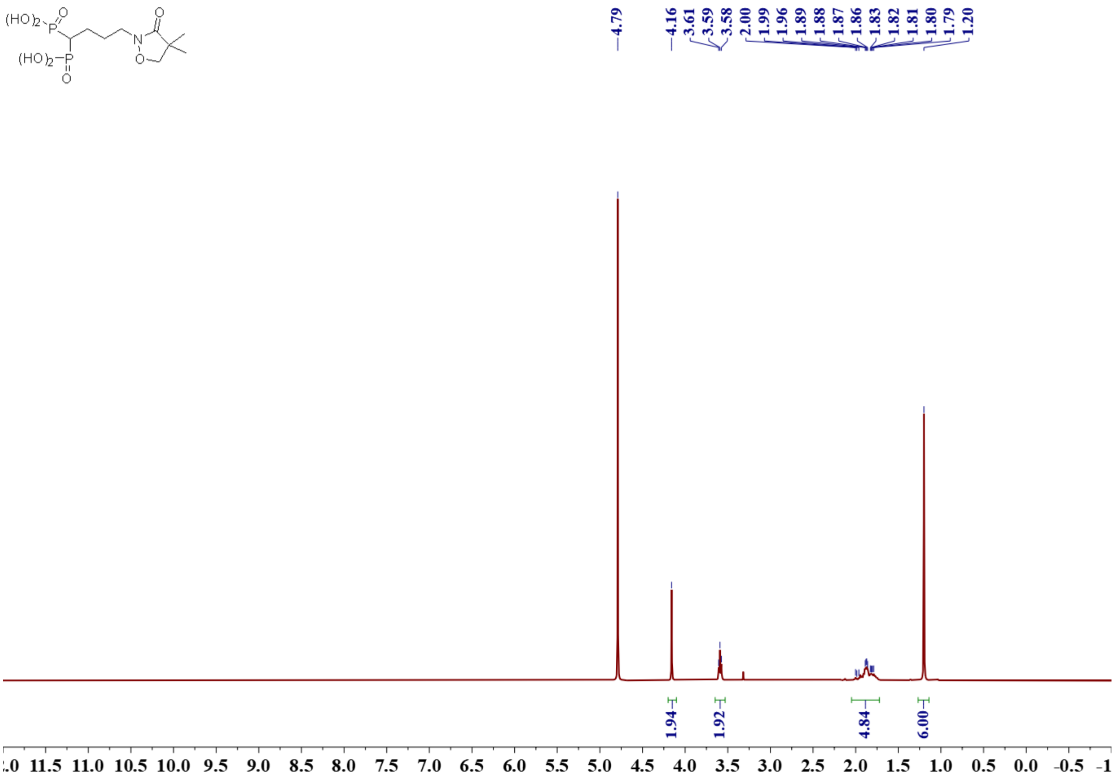

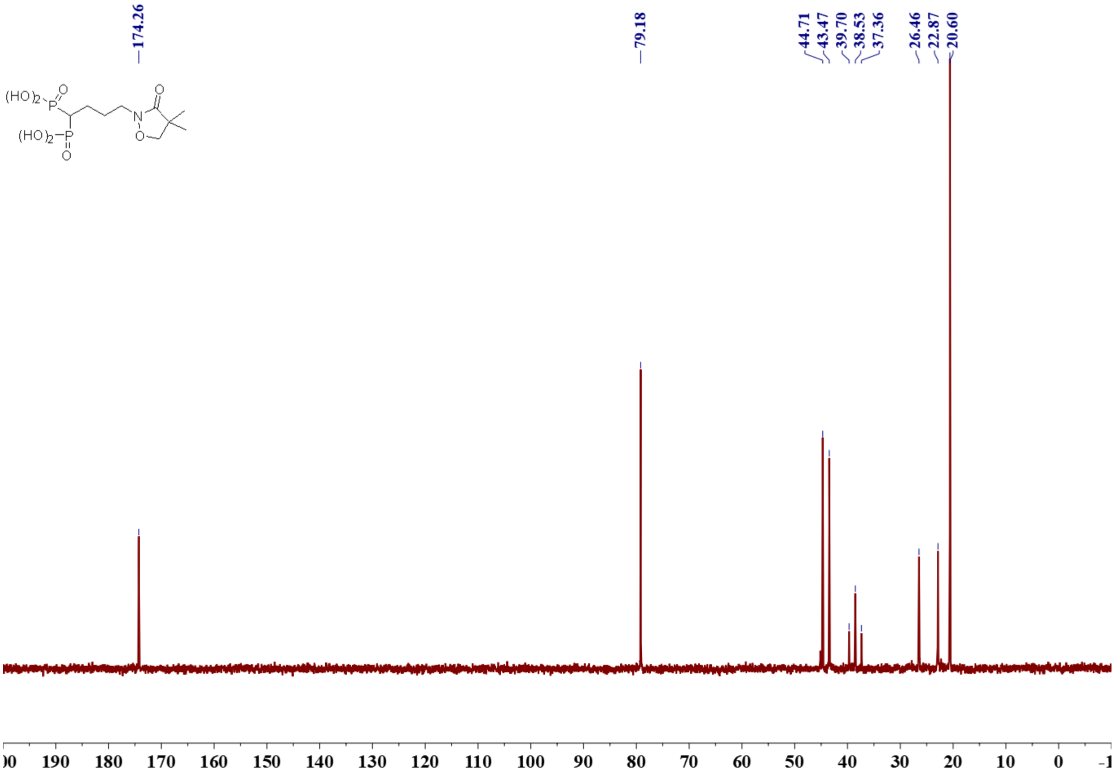

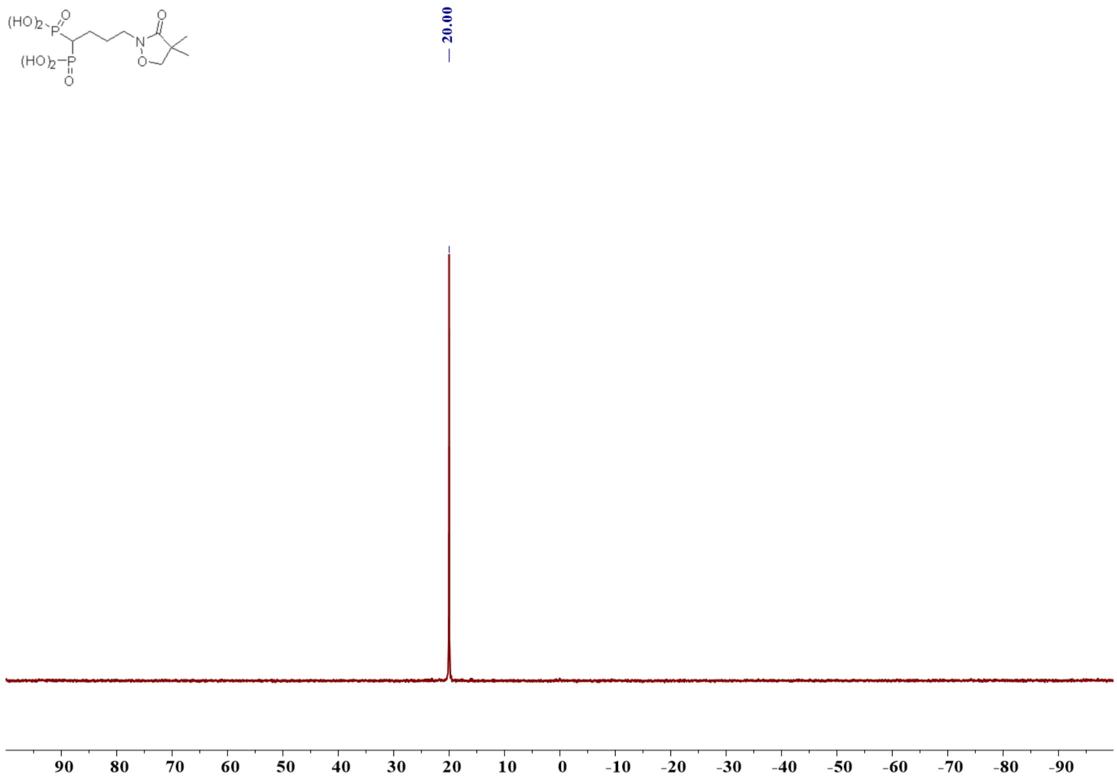
(HO)2
P
O
(HO)2
P
O
N
O
O
20.00
90
80
70
60
50
40
30
20
10
0
-10
-20
-30
-40
-50
-60
-70
-80
-90
